# Awakening the sleeping giant of urban green in times of crisis—coverage, co-creation and practical guidelines for optimizing biodiversity-friendly and health-promoting residential greenery

**DOI:** 10.3389/fpubh.2023.1175605

**Published:** 2023-06-28

**Authors:** Sonja Mohr-Stockinger, Simone J. Sanft, Frederike Büttner, Sylvia Butenschön, Rhea Rennert, Ina Säumel

**Affiliations:** ^1^Kommunen für biologische Vielfalt e.V., Radolfzell, Germany; ^2^Institut für Geographische Wissenschaften, Freie Universität Berlin, Berlin, Germany; ^3^Büro für Bürger:innenbeteiligung des Bezirksamts Charlottenburg-Wilmersdorf, Berlin, Germany; ^4^Department of Urban and Regional Planning, Chair of Urban Conservation and Urban Cultural Heritage, Technische Universität Berlin, Berlin, Germany; ^5^The Integrative Research Institute on Transformation of Human-Environment Systems (IRI THESys), Humboldt-Universität zu Berlin, Berlin, Germany

**Keywords:** co-creation, environmental justice, ecosystem services, green gentrification, green regeneration, nature-based solutions, residential greenery, social cohesion

## Abstract

As multiple crises deepen existing inequalities in urban societies within and between neighborhoods, strategically integrating nature-based solutions into the living environment can help reduce negative impacts and improve public health, social cohesion, and well-being. Compared to public green such as parks, semi-public residential greenery is rarely studied, is regularly overlooked by planners, and often receives step-motherly treatment from architects and housing companies. We approximated the area of residential greenery of modernist multi-story apartment complexes in Berlin, Germany. We surveyed residents’ suggestions for improving their living environments in vulnerable neighborhoods, report on co-creation experiences, and provide a practical guideline for optimizing health-promoting residential green spaces. The semi-public open space on the doorstep of two-thirds of Berlin’s population is highly fragmented and, in total, has a similar area as the public green spaces and a great potential for qualitative development. Just as the suitability of different nature-based solutions to be integrated into the residential greenery depends on building types, resident demands differ between neighborhoods. Residents called for more involvement in design, implementation, and maintenance, frequently proposing that biodiversity-friendly measures be included. As there is no universal solution even for neighborhoods sharing similar structural and socioeconomic parameters, we propose, and have tested, an optimization loop for health-promoting residential greening that involves exploring residents’ needs and co-creating local solutions for urban regeneration processes that can be initiated by different actors using bottom-up and/or top-down approaches in order to unlock this potential for healthy, livable and biodiversity friendly cities.

## 1. Introduction

Existing inequalities in our societies are exacerbated in times of crisis (1, 2). People’s perception of crises depends on whether, and to what extent they have resources available to deal with their impacts (3). There is growing evidence that urban green and blue infrastructure can mitigate crisis impacts and strengthen citizens’ resilience by contributing to public health, social cohesion and overall well-being (4–10) and influencing also real estate market (11). Unfortunately, however, socio-economically vulnerable neighborhoods are often exposed to more environmental stressors such as noise, air or water pollution, and heat stress, but have less access to high-quality urban green and blue structures, even though they need them most. This is conceptualized as environmental (in)justice (12, 13). The need for green regeneration of our cities, and in particular in those neighborhoods that suffer environmental injustice, is already being considered by urban planners, developers and administrators (14–16). Although general strategies for greening cities and promoting urban biodiversity are becoming more common (17), standard implementations beyond demonstration projects remain rare and do not cover the city as a whole (4, 18, 19). For example, the focus of urban planners tends to be on urban parks and public green (7), often underutilizing the greening potential of other spaces. Mainstreaming and practical guidelines facilitating incorporation of nature-based solutions into daily urban design practice of those areas are still largely absent (18, 20).

The residential environment is an urban space where people spend time every day and can host many nature-based solutions such as gardens, green roofs and facades, ponds or pergolas. Such residential greenery, defined as the green (and partially blue) of the immediate surroundings of the residential buildings regularly created in connection with the construction of the respective settlements (21), is rarely studied, is often overlooked by planners and is accorded step-motherly treated by architects and housing companies (21). These green structures undergo continuous changes over time according to the individual developmental pathways of the housing estate including socio-economic ups and downs (22–26). At the same time, due to the compact city approach, it is under strong pressure from urban (re)densification trends (27, 28).

The perspectives on green around housing developments are very diverse. Many studies in the last decade have shown that property value gains from green in their surroundings (29), and can lead to green gentrification (30, 31) and urban green grabbing (when new residential projects are placed adjacent to existing or new green spaces; (32)). For communal housing companies and “non-profit housing associations”, the residential green on their property and its design and maintenance are seen primarily as a cost factor, to be optimized in terms of duration, cost and layout (33). As gardening and maintenance costs are added to the operating costs of the apartments, they are passed on directly to the tenants who, though the greatest beneficiaries of high-quality space, also have an interest in reducing costs. At the same time, due to their public accessibility, such green spaces provide multiple ecosystem services to the neighbors and beyond (34), and, during the pandemic, were of daily benefit to local residents (9). While passive uses (e.g., enjoying the sun and fresh air) outweighed active uses (e.g., meeting neighbors, doing sports; (35)), during lockdowns, residents used these green spaces even more often and more actively as a health promoting resource and also as a space to overcome isolation and to meet neighbors. Thus, the role of residential greenery as a social tissue within the urban fabric increased (9).

Again, although green space is commonly associated with biodiversity (36), we know little about residents’ preferences regarding biodiversity-friendly compared to “just greening” measures. Besides, as neighbors’ preferences on residential green can be quite antagonistic regarding different use or design options (e.g., wild growing vegetation vs. English lawn; (9, 35)), enhancing the welcoming qualities and the motivation to be physically active by implementation of adequate elements is crucial.

Drawing on experiences of testing different governance approaches for co-creation of nature-based solutions under conflicting stakeholder interests to ensure inclusiveness in urban regeneration projects (20, 37, 38), optimization of health-related services of residential green can involve diverse actors and governance constellations for enhanced multifunctionality and to maximize the adaptability to diverse and changing residents’ needs across different cultures and generations (9) in times of crisis and beyond.

Here, firstly, we aim to access the area covered by the semi-public residential greenery of the modernist housing complexes in Berlin, Germany. Secondly, we take a step forward by linking scientific results regarding the status quo and functionality of residential greenery with decision making on the implementation of health promoting nature-based solutions nearby multi-storey housing complexes. We do this by analyzing residents’ suggestions regarding green regeneration, biodiversity-friendliness, quantitative and qualitative development of the residential greenery, by sharing insights from co-creation workshops with local residents, and by proposing practical guidelines to optimize health promoting residential greenery to the benefit of all.

## 2. Materials and methods

### 2.1. Study area

Our study focuses on the residential greenery of modernist housing complexes, similar to those in almost all Central European cities, that are home to two thirds of the Berlin population. We took a closer look at the residential greenery at eight study sites in the most disadvantaged residential neighborhoods of Berlin, Germany, areas with high noise and air pollution, high bio-climatic stress, low social status indexes and low access to green spaces, identified on the basis of the Environmental Justice Map of Berlin (SenStadtUm (39); see details in Battisti et al. (40) and Supplementary Figure S1). The social status index combines indicators covering percentage of beneficiaries of social welfare, inhabitants with migration background, old-age poverty, child poverty, and single-parent households (39). Two of the sites are in East Berlin and can be categorized as real estates with post-socialist heritage (Berlin Mitte and Marzahn).

**Figure 1:**
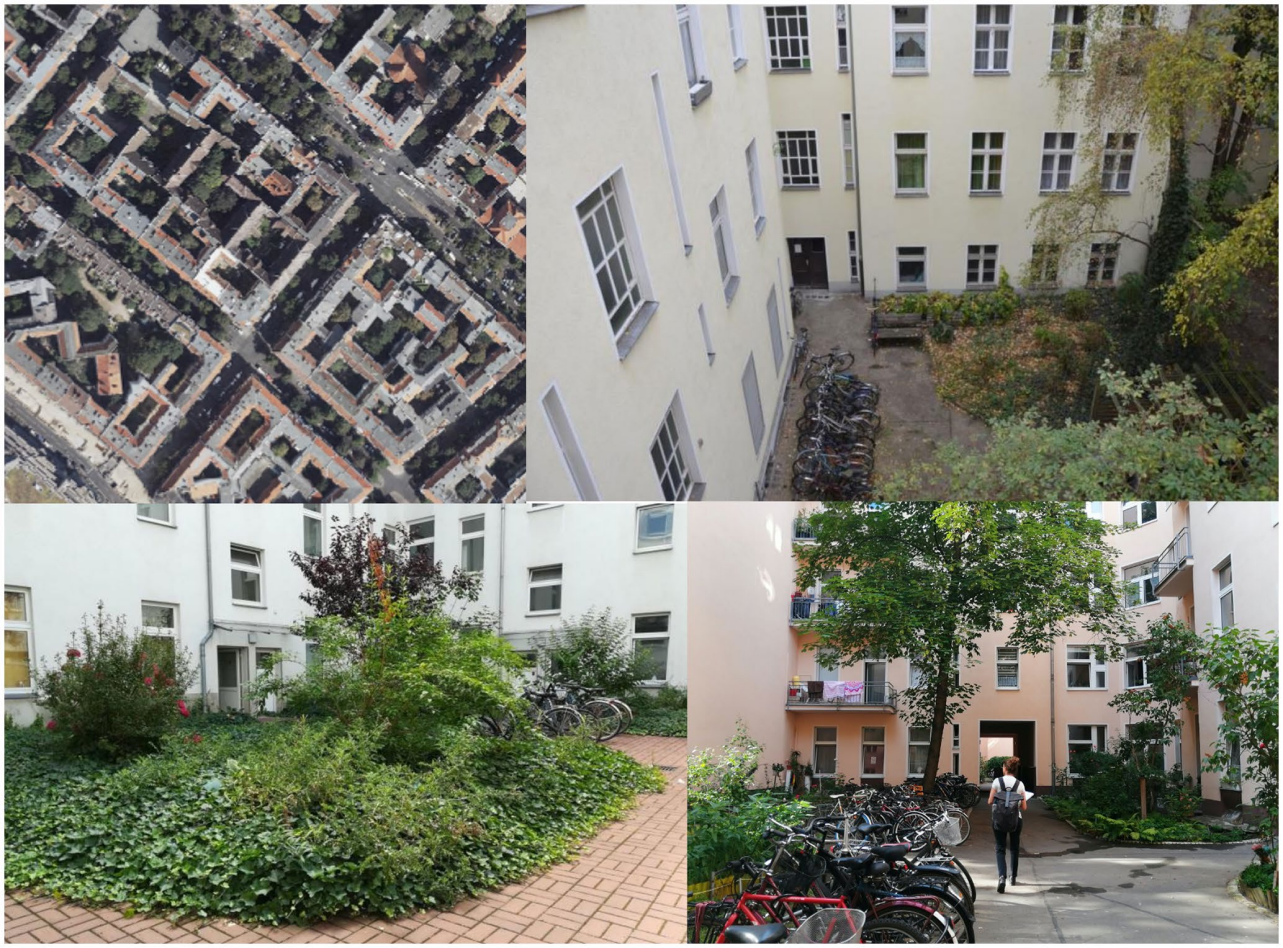
Residential greenery of dense block-edge development of the Wilhelminian era examples from Berlin-Wedding and Neukölln (SenStadtWo, Orthofotos August 2020; Geoportal Berlin; Photos: *HealthyLiving*).

The block developments from the years 1870–1918 are (almost) closed, mostly 4–6 storeys, with a front building, side wings and rear building (Figure 1). Their backyards are dominated by concrete courtyard areas, and some feature isolated flower beds, shrubs or single trees.

**Figure 2:**
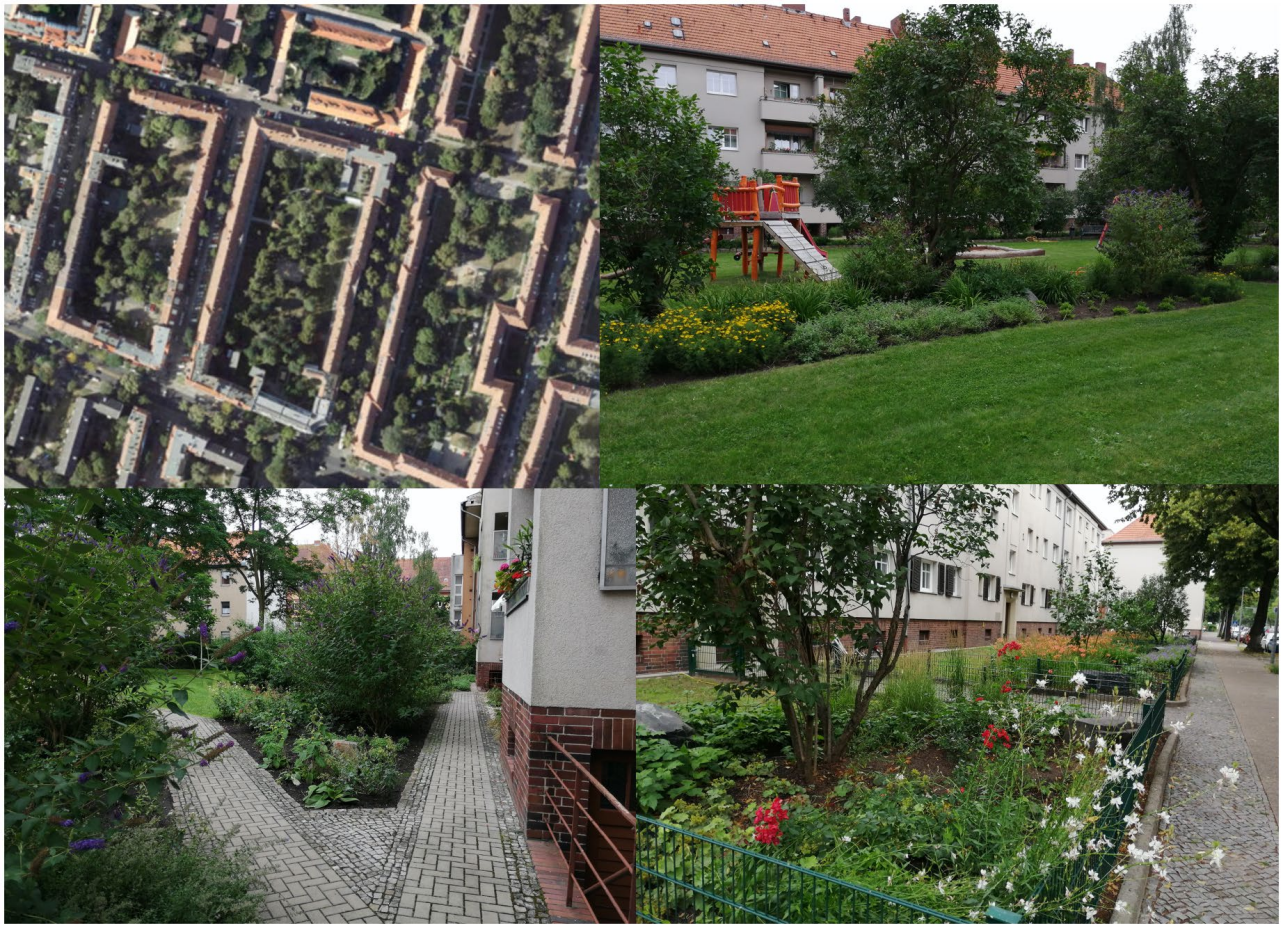
Residential greenery of reform-oriented perimeter block development with courtyards in Berlin-Reinickendorf (SenStadtWo, Orthofotos August 2020; Geoportal Berlin; Photos: *HealthyLiving*).

The reform-oriented perimeter block developments, 3–4 storey (almost) closed structures, were built between the 1920s and the 1940s. They feature larger inner courtyards, usually containing a lawn, sometimes garden plots and a few trees (Figure 2), and also some paved or concrete areas.

**Figure 3:**
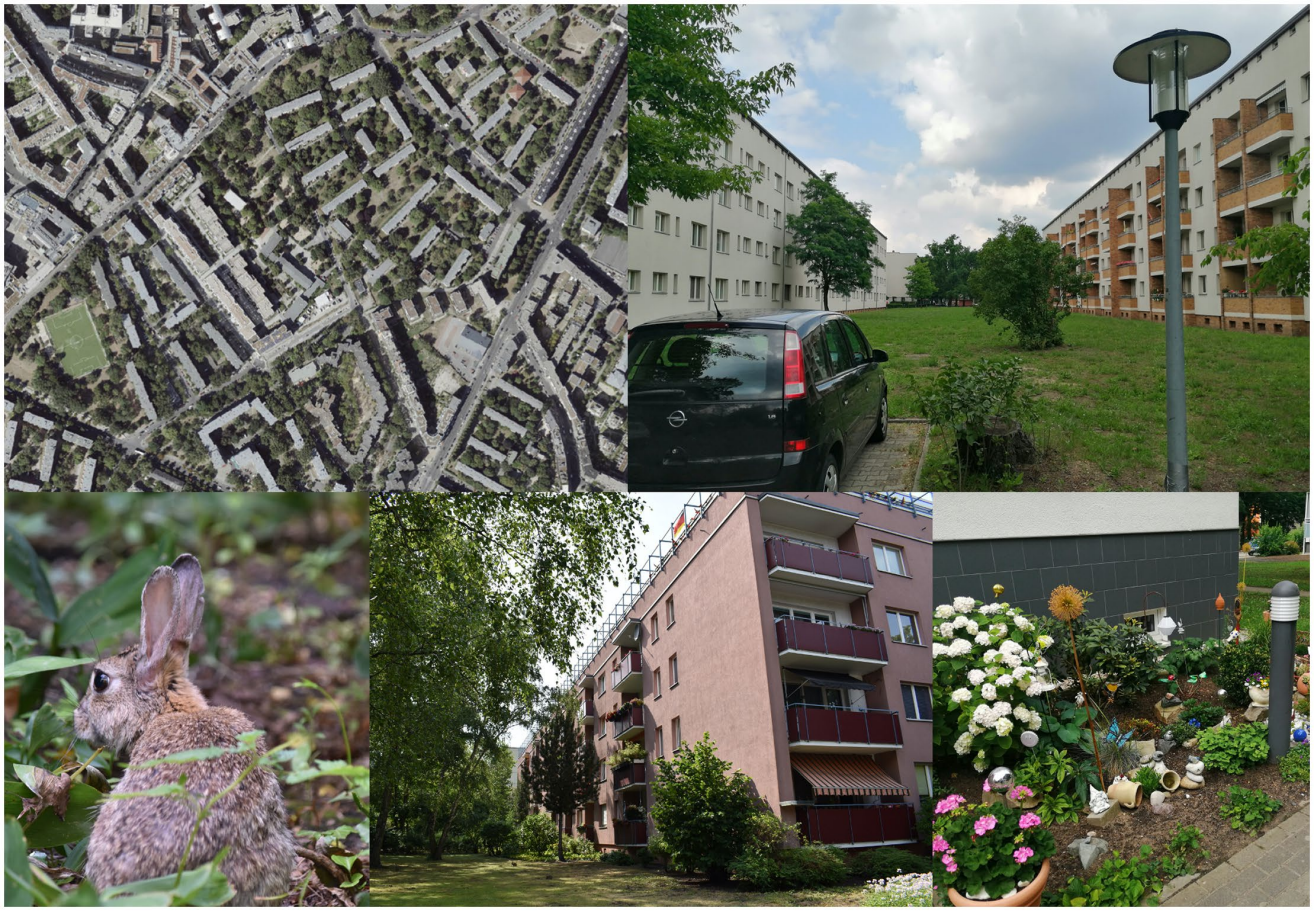
Residential greenery of row development with courtyards, examples from Berlin-Mitte, Charlottenburg and Spandau (SenStadtWo, Orthofotos August 2020; Geoportal Berlin; Photos: *HealthyLiving*).

Row developments from the 1920s to the 1970s consist mostly of 4-storey rows of houses, often in a row, resulting in larger, elongated, interconnected open spaces (Figure 3). These are usually in the form of lawns with selective bushes and trees.

**Figure 4:**
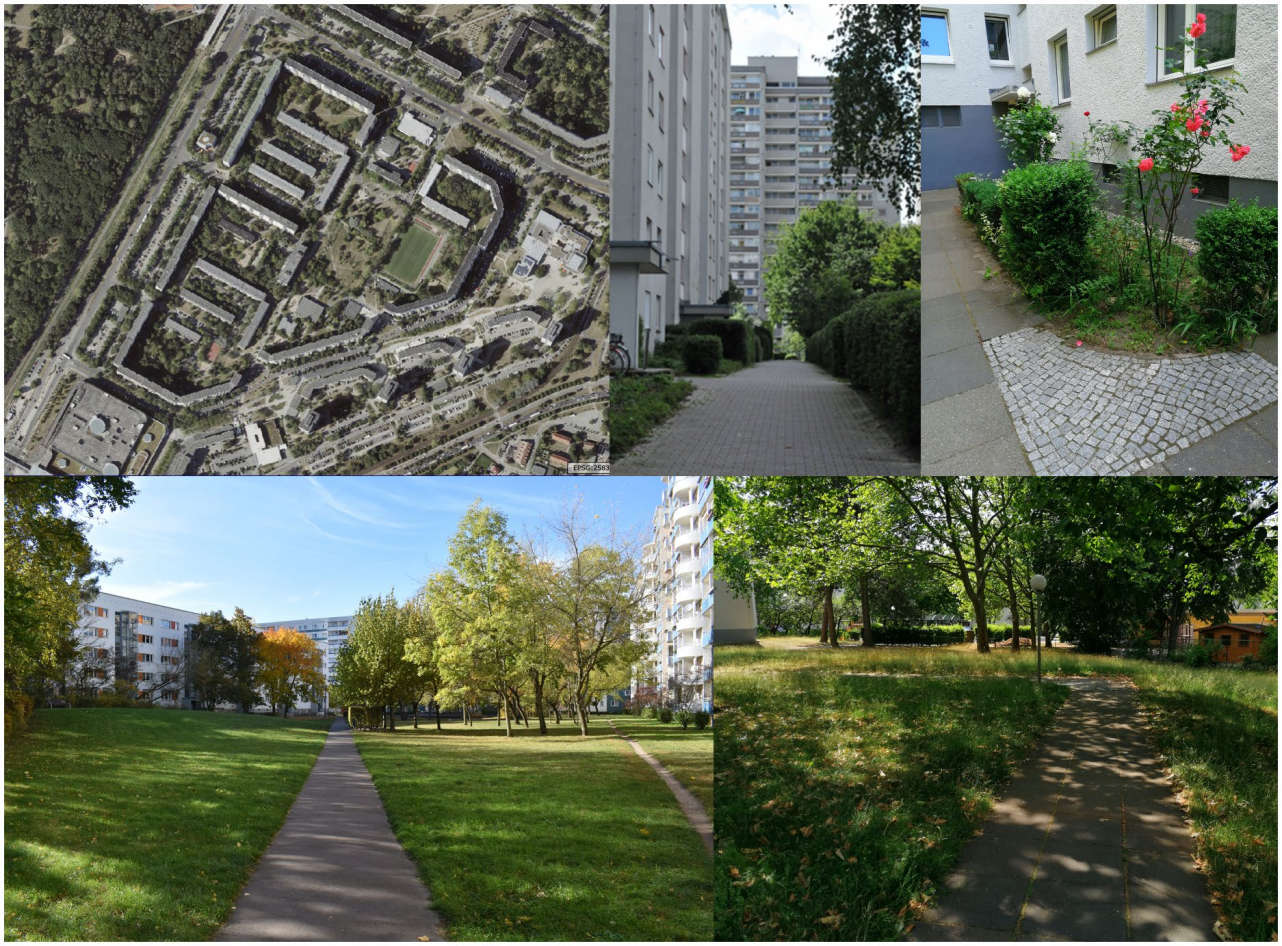
Residential greenery of large housing estates and high-rise buildings in Berlin Marzahn and Gropiusstadt (SenStadtWo, Orthofotos August 2020; Geoportal Berlin; Photos: *HealthyLiving*).

The high-rise buildings/prefabricated buildings of the 1960s to the 1980s are rows or point houses with different block or row constructions, usually over 6 storeys high (Figure 4). Most of the undeveloped areas are covered by lawns with some trees or shrubs and ornamental gardens with access paths, and parts concreted over as parking lots.

Today the residential greenery of multistorey modernist housing complexes originally designed in these different epochs do not share a common mode of landscape architecture. While woody species mapped in the residential greenery have a medium to high air filtration capacity, one to two thirds of the planted species have a high allergenic potential (34). All residential greeneries have common elements that support physical activities and related healthy lifestyles such as bike racks, benches, playgrounds, partially sealed parking lots and spaces for garbage containers. Nature-based solutions like bioswales, facade-attached greenery, atriums, fountains, or ponds are rare (34, 40).

### 2.2. Assessment of semi-public residential greenery of the modernist housing complexes

In order to capture the development potential of residential greenery for the integration of nature-based solutions, the area of settlement green space was calculated from the areas of the respective “urban structure types” of block development from the 1870s to 1940s, row development from the 1920s to 1970s, and high-rise buildings from the 1960s to 1980s that were predominantly prefabricated (i.e., Nos. 1, 2, 10, 72, 9, and 11; (39)), subtracting building forms and sealed surfaces, public green spaces, and playgrounds (41). The geodata were processed in QGIS v3.10.11-A (42).

### 2.3. Interviews on suggestions to improve residential greenery

In summer 2018 and 2021, we conducted 270 face-to-face interviews with residents at the eight study sites that represent the four main building types in Berlin. We collected basic information on demographic data, use and perception in closed questions (9) and, in an open-ended question analyzed using content analysis (43), suggestions to optimize benefits of the residential greenery on health and wellbeing, with a focus on nature-based solutions. Based on the responses, statements were categorized on content and keywords. One category of people referred to (green) nature-based solutions (e.g., green; trees; flowerbeds; lawns; greenspace; green facades; green roofs), another category explicitly mentioned biodiversity related aspects and/or benefits of nature-based solutions (e.g., meadows instead of lawns; a third mentioned concrete plant or animal species, wilderness, biodiversity), a fourth did not mention nature-based solutions (Supplementary Table S1). Each individual keyword was assigned to one or more of the categories and so transformed into a variable. Respondents were first categorised as “Green Supporters”, “Biodiversity Friends” and “Others”, and second as respondents claiming quantitative, and/or qualitative enhancement of residential greenery, and “Others”. We developed categories based on the respondents’ statements on the open question on suggestions with similar content regarding green, biodiversity friendly or other solutions that were mapped to a category (Supplementary Table S1). The survey data were analyzed using R (44) to test cross-tabulated ordinal data for independence with the chi-squared test.

### 2.4. Co-creation workshops on re-design of residential greenery

The co-creation workshops were piloted in the district of Marzahn-Hellersdorf in a neighborhood with prefabricated buildings of the 1980s, where a new housing construction project is also leading to redesign of existing residential greenery. In total about 50 attendants participated in three different discussion rounds, including residents and relevant local stakeholders.

**Figure 5:**
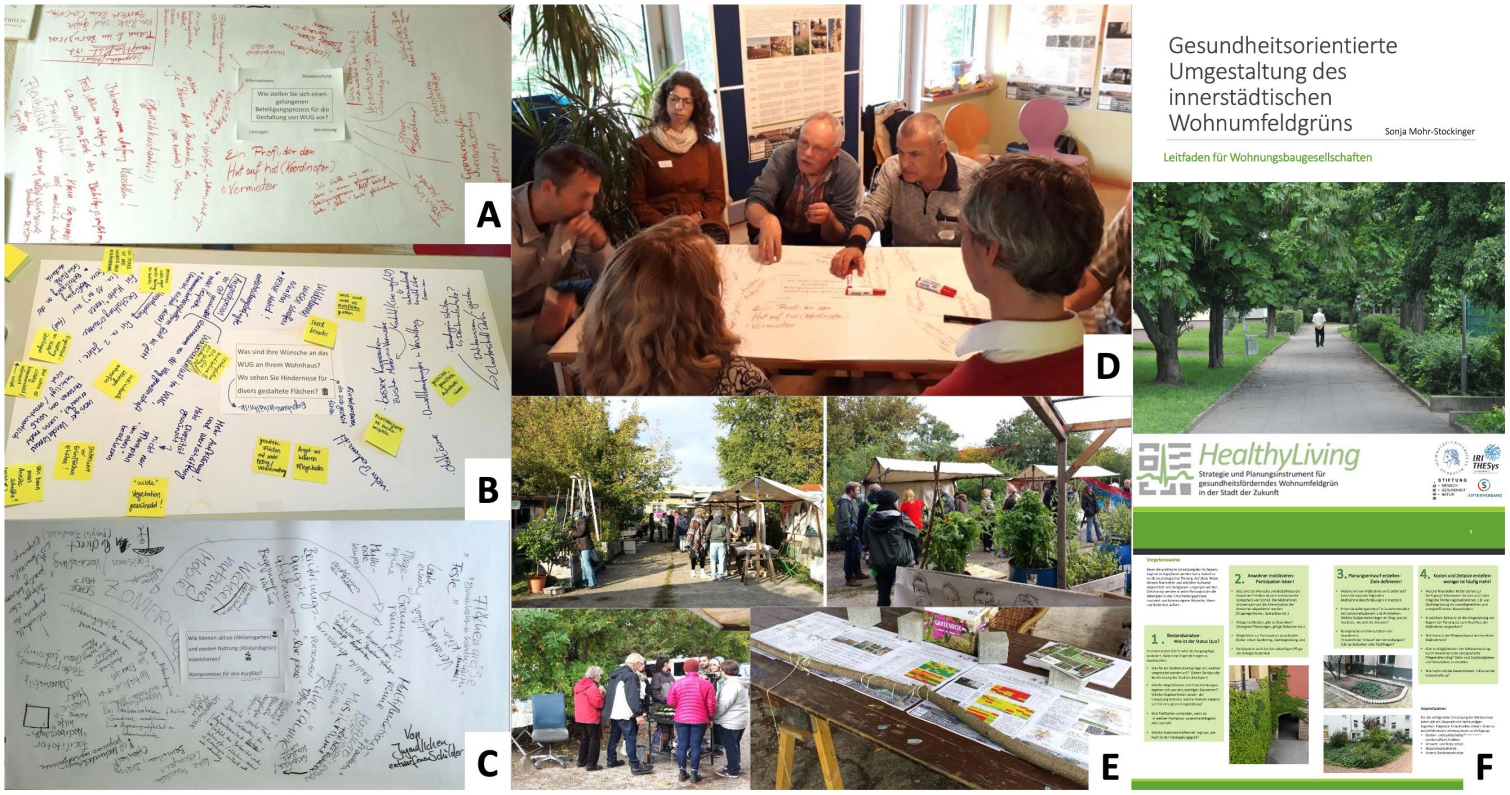
Impressions from the co-creation workshops with residents and relevant actors in Berlin Marzahn-Hellersdorf (**(A–E)**; Photos: Frederike Büttner). Target group specific, simple and user-friendly: Screenshots from the guideline brochure for implementation of nature-based solutions (in German) for object planners, housing companies and residents (**(F)**; see Guidelines in the Supplementary material).

A first workshop was set up in May 2019 (Figures 5A–D), bringing together the target groups of residents, the housing company, a local NGO, gardeners of a local community garden, employees of the municipality administration and scientific researchers. After a short introduction to the background (including previous survey results; (35)), a world cafe format was used to discuss necessities and obstacles of redesigning the residential greenery and to establish different interests and needs of the participants. The three main topics were: (A) Envision a successful participation process for redesigning residential greenery; (B) What does your residential greenery ideally look like?; (C) Dealing with contrasting interests. The participants had about 20 min per discussion round. Ideas, suggestions and critical comments were collected on blank posters. The answers were then categorized in six different clusters (communication and information, coordination, design suggestions and needs, responsibility, biodiversity, and concerns; see Supplementary Table S1).

A second workshop was held on October 3rd, 2019—a public holiday—integrated into the garden autumn celebration of the local community garden (Figure 5E) in order to address, in particular, the local residents in a more informal atmosphere than in the previous workshop. Presenting the results from the first participation event was used to get the resident’s attention, to stimulate and deepen the discussion in personal conversations, and to lower the barrier for residents to make contact and to discuss possible improvements for the residential greenery.

### 2.5. Developing a practical guideline

For the drafting of our practical guideline, we adapted the following steps proposed by De Montis et al. (45): (i) analysis of status quo of residential greenery, (ii) context specific SWOT analysis to identify needs and define objectives for actions, (iii) consistency check with other guidelines and strategies in place, (iv) drafting of guidelines tailored to the specific geographical and institutional context, (v) presentation of the draft to acquire views and comments from interested parties, and (vi) verification on the final contents of the GI guidelines with representatives of housing companies, landscape architects and other potential users. Results of the steps i–iii have been published in several papers [e.g., (21, 34, 35, 40, 46, 47)], and the results of step v are presented here and in Mohr-Stockinger (46). Step vi is ongoing.

## 3. Results

**Figure 6:**
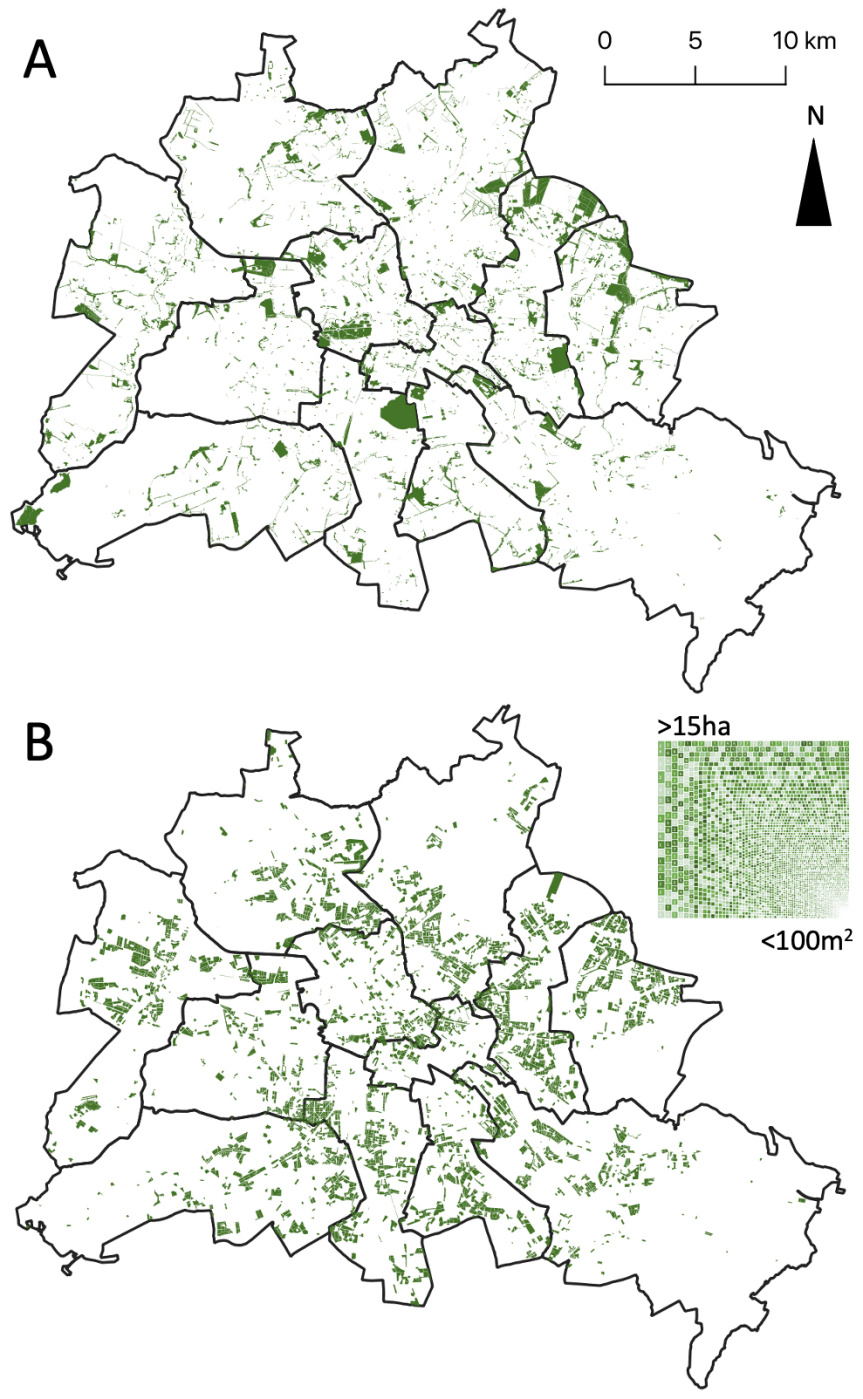
Public green space and playgrounds **(A)** and semi-public residential greenery of modernist housing complexes consisting of diverse patch sizes **(B)** of Berlin, Germany. District borders of Berlin are given.

**Figure 7:**
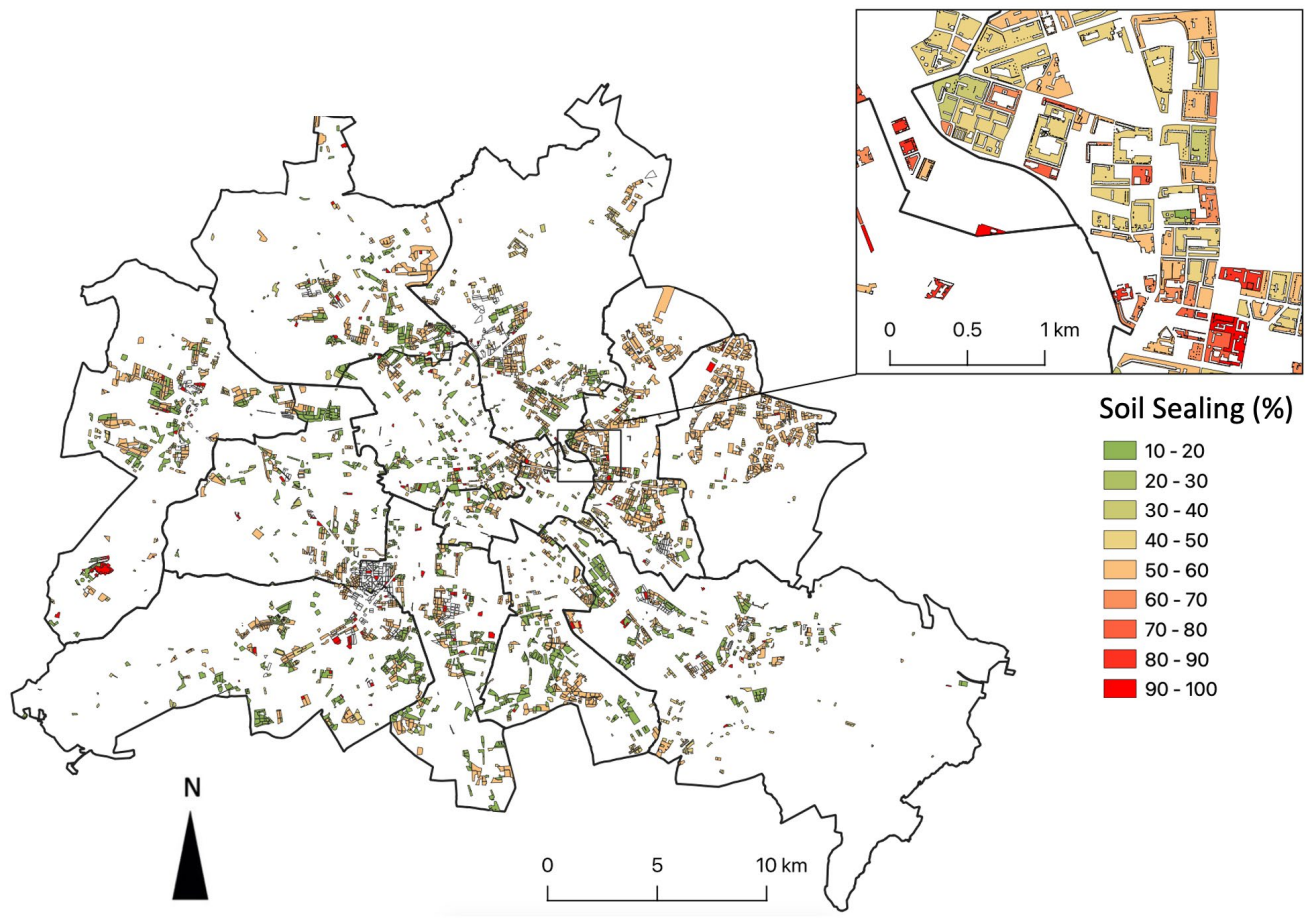
Soil sealing in the semi-public residential greenery of modernist housing complexes of Berlin, Germany. District borders are given.

While public green covers about 53.8 million m^2^, of which 31.6 million m^2^ are public parks (Figure 6A; Geoportal Berlin), residential green covers an undeveloped area of 43.6 million m^2^, with area sizes varying from areas of less than a 100 m^2^ between buildings to several hectares of green space with scattered buildings, with a median of about 7,400 m^2^ (Figure 6B). Soil is partially sealed through passages, parking lots and paved areas for garbage containers and ranges between 10% and 60% (Figure 7).

### 3.1. Green or biodiverse, more or better?

In order to define residents’ demands, we asked a total of 270 people for suggestions to optimize health related benefits of their residential greenery. Of the respondents, 43% were male and 57% female, half were between 31 and 60 years old, and had, on average, lived 17 years in their respective neighborhood (see details in Säumel and Sanft (9)). Overall, residents were generally very satisfied with the greening of their living environment and, regardless of the neighborhoods’ structural differences, half did not suggest any improvements regarding nature-based solutions within the residential green space (Table 1), while 40% expressed the need for enhanced greening, and about 16% mentioned measures to enhance biodiversity friendliness. In addition, around 20% suggested spatial or numerical expansion of green elements such as more trees or larger green areas, and nearly 40% demanded a qualitative improvement of green structures. The need for measures to qualitatively enhance residential greenery was more often expressed in the dense and closed block-edge developments of the Wilheminian era. Residents of the large housing estates with towers and high-rise buildings from the 1960s to 1980s most often asked for more green, while residents of the parallel and free row development within landscaped residential greeneries of the 1920s–1970s asked more often for biodiversity friendly measures.

**Table 1:** Number and percentage of residents suggesting an enhancement of residential greenery regarding greener and/or biodiverse residential greenery (A) or regarding quantitative and/or qualitative development (B).

		A: Green or biodiverse?			B: More or better?		
	All	Green supporters	Biodiversity friends	Others	Quantitative	Qualitative	Others
*N*	270	106	43	158	56	106	142
%	100	39	16	58	21	39	53
Comparison between structural types							
*χ*^2^		2.1	0.9	0.6	0.6	1.4	0.8
df		2	2	2	2	2	2
*p*		0.341	0.627	0.740	0.736	0.442	0.665
Comparison between neighborhoods							
*χ*^2^		22.1	27.9	9.8	33.2	20.8	13.0
df		7	7	7	7	7	7
*p*		0.002	<0.001	0.201	<0.001	0.004	0.072

Some respondents are classified in both categories on the basis of the keywords (see Supplementary Table S1). “Others” did not mention any keywords. Comparison between block edge development (Block), row development (Row) and high-rise buildings (High). Chi-squared test, df and *p*-values are given. See full table in Supplementary Table S2.

There were also differences between neighborhoods, independent of the general building structures or the location of a neighborhood along an urban–rural gradient. For example, residents of the inner city Ideal-passage in Berlin/Neukölln and Alte Jacob Str. in Berlin/Mitte more often suggested greening measures, and in the latter neighborhood the need for biodiversity friendly measures were also more often mentioned compared to other inner city block edge or row developments (Table 1 and Supplementary Table S2). Quantitative development of the residential greenery was especially suggested in the dense block edge developments and in the neighborhoods of East Berlin (Alte Jacob Str. and Marzahn), whereas qualitative enhancements were mentioned most in the Sprengelkiez in Berlin/Wedding and Alte Jacob Str. in Berlin/Mitte.

### 3.2. Co-creating healthy residential greenery

Although three different topics were discussed in the workshops (Supplementary Table S3), similar aspects arose in all discussion rounds. These are summarized next.

#### 3.2.1. Envisioning a successful participation process

Nearly half of the comments mentioned enhanced communication measures to inform neighbors about the process. One third wanted to take more responsibility for the residential greenery, including joint maintenance, training for residents, and its integration into the rental contracts. The main goal of co-creation is to build up a stronger community within the neighborhood, so networking and communication are essential. The residential greenery can then be designed and divided into smaller sections, facilitating neighbors taking responsibility for plot maintenance. Neighbor groups, consist of a housing unit, could meet, discuss problems and solutions and distribute tasks and do voluntary work. Neighbors are willing to coordinate design and management themselves. Time capacity and flexibility of neighbors need to be considered. Some neighbors mentioned “Subbotniks” that were common when the neighborhood was built in times of the GDR.^1^ The attendees believe such self-growing structures can also function today, starting from smaller core groups and facilitated by the housing companies. A column in the housing newsletter could motivate and mobilize the residents to participate. To ensure the equal participation of all willing to get involved, the co-creation process and communication between neighbors and housing companies should be moderated by a coordinator. To get from the ideas and brainstorming to a successful redesign of the residential greenery, communication needs to be clear and honest from the beginning, including doubts and problems. Conflicts of interests can be discussed in an open dialog within the housing units to find a solution where everybody feels involved, including the quieter ones. The attendees proposed that design workshops could help better understanding and to illustrate usage possibilities to support the decisions made. The redesigned areas are designed, managed and maintained by the residents, with reliable support from the housing company providing technical support and water supply. Semi-annual seminars, by experts on gardening topics, could help deepen residents’ knowledge and increase their confidence to maintain a tenants’ yard.

#### 3.2.2. Your ideal residential greenery

Attendees evaluated the current design of the residential greenery as very simple, and the participation by the local residents as low. Many clearly expressed their appreciation for the residential greenery and the motivation to help in redesigning the area. They proposed higher appreciation of the residential greenery by the housing companies and a focus on their personal needs as well as more education on the significance of the green and its unused potential. A third of the comments demonstrate willingness to actively take responsibility, asking for tenant plots and more individual designs. Small scale pilots could provide first practice examples. Having a direct contact person and generally lowering the barriers to interact with the housing company was also often mentioned. The residents ask for more structured areas with sections such as protected, cozy lounges and meditative places with elements of tranquility that invite lingering, as well as space for gardening and their own designs. Neighbors want more individual solutions for the different locations, not “an easy-care planting plan” without accessing the areas and consulting the local residents. Every fifth comment was related to biodiversity aspects (e.g., a “wilder vegetation” instead of plain lawns; implementation of wildflower meadows, more individual planting plans, fruit trees and rooftop gardens). The residents miss opportunities to garden and expressed special interest in taking responsibility for small plots of 10 m^2^, which they would plant and design independently and are willing to maintain. Better participation promotes the identification of the residents with their place of living and less vandalism is expected if many people feel responsible and associated. Still, the housing companies, as the owner of the sites, need to keep in contact and should be easily and informally accessible in case of questions and problems. The attendees recommended a person in charge, who attends the tenants’ meetings occasionally.

#### 3.2.3. How can contrasting demands of neighbors coexist?

The majority suggested measures like zoning, alternating use and multifunctionality. Individual solutions for the different areas and sections of the residential greenery provide opportunities to take the diverse needs into account. Enhanced coordination and exchange yard celebration and community spaces help to reduce anonymity, prevent conflicts and improve the communication within the neighbors and with the housing companies, and so build trust. In this round, residents also asked to be involved in the design of their residential greenery. With smaller, self-maintained plots, alternating usage is also possible. The residents wish to make decisions themselves about the seedlings, plant selection and to design the plots provided. Additional co-operation with neighbor’s green spaces, for example watering during holiday season, will lower burdens. The attendees requested that housing companies provide facilities for gardening activities such as a water supply in the yards. Building greening, structural elements and different colors can redirect the gaze away from the lawn as well. They argued that greenery as a spacing element will then slowly lose its relevance. A smart design shaping small, individual spaces and zoning enables the coexistence of active and passive use side by side and allows adjustments to the different needs. Barrier-free access to the area should be ensured. The participants envisioned a separation of areas with active and passive usage and suggested linking the apartment size and the design of the greenery in new building projects, which enable flexible floor plans and possible adaptation to changing living conditions.

### 3.3. Practical guideline and portfolio of NBS for different building structure types

The guideline to optimize health promoting residential greenery (Figures 5G,H; in German and English translation in the Supplementary material) informs housing companies and their tenants by including a portfolio of different nature-based solutions suitable for the four most common building types in Central European cities and a decision matrix. Each measure is described and its health-related potentials highlighted (Tables 2, 3; see details in the Supplementary material).

**Table 2:** List of recommendations for implementation of various greening measures in relation to building structures block development (A) and reform-oriented perimeter block development (B) (see detailed descriptions health-relevant ecosystem services provided by these measures in the Supplementary material).

NBS	Block development	Reform-oriented perimeter block development
Green roof (GR)	Roof pitch and statics of the building often do not allow GR. Pot planting on existing terraces is preferable.^*^	
Façade greening (FG)	FG is highly recommended, due to a high degree of sealing and the small proportion of open spaces close to the ground. Give preference to climbing aids for ground-based systems. If the structure is porous, take care of the root and tip growth of the tendril and cut it back regularly.^*^	
Meadows & wild shrubs (MS)	Paved or concrete inner courtyards often do not allow for lawns. The height of the building and the size of the courtyard determine the incidence of light. Wild perennials can be grown in tubs or raised beds and can be integrated into every yard.	Depending on the size of the yard and the degree of sealing, MS can be realized. Note the incidence of light and plant wild perennials. If there is not enough free space, place raised beds or tubs in sun-exposed areas.
Open space (OS)	The OS design depends on the size of the yard and the demands of the residents. If there are children, sandboxes and swings can be installed in the yard, regardless of the degree of sealing. Raised beds invite to do gardening, while benches and tables lure residents outside. Even in a small space, create an opportunity for residents to stay and spend their free time together.	
Bodies of water (BW)	Although BW are difficult to integrate and take up a lot of space, they are indispensable for city animals. Provide bird baths.	In larger courtyards, small BW can be easily integrated. If not possible, create other water sources for city animals in the form of bird baths.
Trees, shrubs, hedges (TSH)	TSH requires an area of at least 5 m^2^ that is free of sealing, but 6 m^2^ is better according to DIN 18916. Note the incidence of light and possible shading from low-lying apartments during the growing season. Information on soil quality should inform species selection. With partially sealed floors, shrubs are an alternative to large trees. Structure the yard by lining borders and parking spaces. If there is a lack of space, plant shrubs in larger containers.	A larger open space means TS can be easily integrated into perimeter block developments. Pay attention to possible shading of the apartments below and a minimum distance to the building. Line parking spaces, flower beds and verges with shrubs to increase the amount of greenery.

^*^Note monument protection regulations that might be in place.

**Table 3:** List of recommendations for implementation of various greening measures in relation to building structures row settlement (C) and large housing estates (D) (see detailed descriptions health-relevant ecosystem services provided by these measures in the Supplementary material).

NBS	Row settlement	Large housing estates
Green roof (GR)	If static requirements are met, intensive or extensive roof greening measures are suitable. If there are flat roofs, raised beds can be added.^*^	Most of the buildings of these structures are best suited for green roof measures. Supplemented by raised beds, they become the new ecological focal point of the residential unit for residents.^*^
Facade greening (FG)	The same applies: facade greening is a simple option for integrating greenery into the living environment and should always be considered. Self-climbing plants and plants with climbing aids make rows of buildings green. If the building structure allows it, wall-mounted systems can be integrated.^*^	Due to the better building fabric, more modern buildings are well suited for wall-mounted systems. If this cannot be implemented for financial or planning reasons, ground-based systems are highly recommended.^*^
Meadows & wild shrubs (MS)	Green spaces between the rows of buildings ensure good incidence of light and are suitable for wild flowers and perennials. Create large or small wildflower beds and complement them with raised beds.	Larger open spaces enable the creation of a diverse wildflower meadow. Ensure that this is step-protected under certain circumstances by releasing other sub-areas.
Open space design (OSD)	Since row developments often include larger units, the diversity of residents is particularly high. Multi-generation parks are particularly recommended here.	The population density and the associated diversity can be served by a multifaceted range of design elements. Create social spaces through seating, multi-generation parks and the joint management of, e.g., vegetable beds.
Bodies of water (BW)	If there is enough space between the rows of buildings, several smaller bodies of water can be integrated in the form of fountains or ponds.	Bodies of water create an ecological and social hotspot on larger open spaces and should therefore be preferred.
Trees, shrubs, hedges (TSH)	Low trees can be planted several times between the rows of houses and can be supplemented by large trees. Fruit trees are pollinator friendly and provide residents with a variety of choices over the long term. Shrubs and hedges create structure between the rows of buildings and can frame paths and parking spaces. The combination of different species is of ecological advantage here.	Both large and small trees can be accommodated on larger systems. Plant a variety of fruit trees that residents of all ages will enjoy, and optionally incorporate maintenance into an urban gardening project. Integrate shrubs and natural hedges as structuring elements. Planting different species creates diversity.

^*^Note monument protection regulations that might be in place.

## 4. Discussion

Even if the saying that crises always represent an opportunity has been overused in recent years, times of crisis do facilitate social innovation and the revision of what is owned and what is needed. Crisis-driven new discoveries, claims and encounters within urban green have been reported for the Covid-19 crisis (9, 48, 49, 50). Our results underline that the provision of cultural and health relevant ecosystems services by urban green was increasingly appreciated, especially for the green of our living surroundings, which is known as residential greenery. In both interviews and co-creation workshops, residents called for multifunctional and structurally rich green spaces near their homes (Table 1). Views out of the apartments’ window on residential green are of great importance especially during lockdowns and for less mobile people (47). Balconies became a green oasis (51) and a space to communicate with neighbors during lockdowns (52, 53). The crisis fostered appropriation of residential greenery for uses such as meeting neighbors or doing sports, and the spectrum of residents’ requirements expanded significantly to active use settings (9) as the green on the doorstep became a crucial refugia for neighbors.

Half of the respondents asked for qualitative and/or quantitative enhancement, with biodiversity-friendliness a crucial feature in their “ideal” residential greenery (Table 1). This suggests growing awareness and nature connectedness, as these topics have not been mentioned often in previous studies (35). However, the interrelatedness of actual or perceived biodiversity of urban green and influencing parameters like species literacy, recreational and health benefits, visitations rates and nature connectedness is still poorly understood, and research findings are inconsistent (54, 55). Biodiversity management within urban green and blue needs to consider multiple scales, negotiated among various actors and accounting for a myriad of influencing factors (56). Biodiversity becomes a topic also for communal housing companies at least in pilots and when it is supported by external funding (57). Planners and landscape gardeners are learning biodiversity-friendly management techniques, dealing with water scarcity or other aesthetics beyond the English lawn. Our results show that residents can be allies and are key actors to be involved in biodiversity-friendly (re)design and management. However, green washing and green branding using nature-based solutions is a widespread phenomenon, especially when developers refer to ecological values and greenery in marketing campaigns (58, 59).

Moreover, our results from Marzahn-Hellersdorf support findings that large housing estates from the socialist era represent relative social stability and offer affordable housing as it has reported for other post-socialist cities (24). In addition, the Berlin housing market is currently under great strain due to a combination of a rising population, a lack of new residential construction and a growing real estate speculation. Rising rents for new rental contracts in the last decade tie tenants to existing rental agreements. Thus, we overserved long residential times in the studied neighborhoods in average of about 17 years (9). In addition to great investments of the housing companies to change the stigmatized neighborhood images, apartments in formerly not so attractive housing estates (also those constructed in socialist times) became attractive for the middle class.

**Figure 8:**
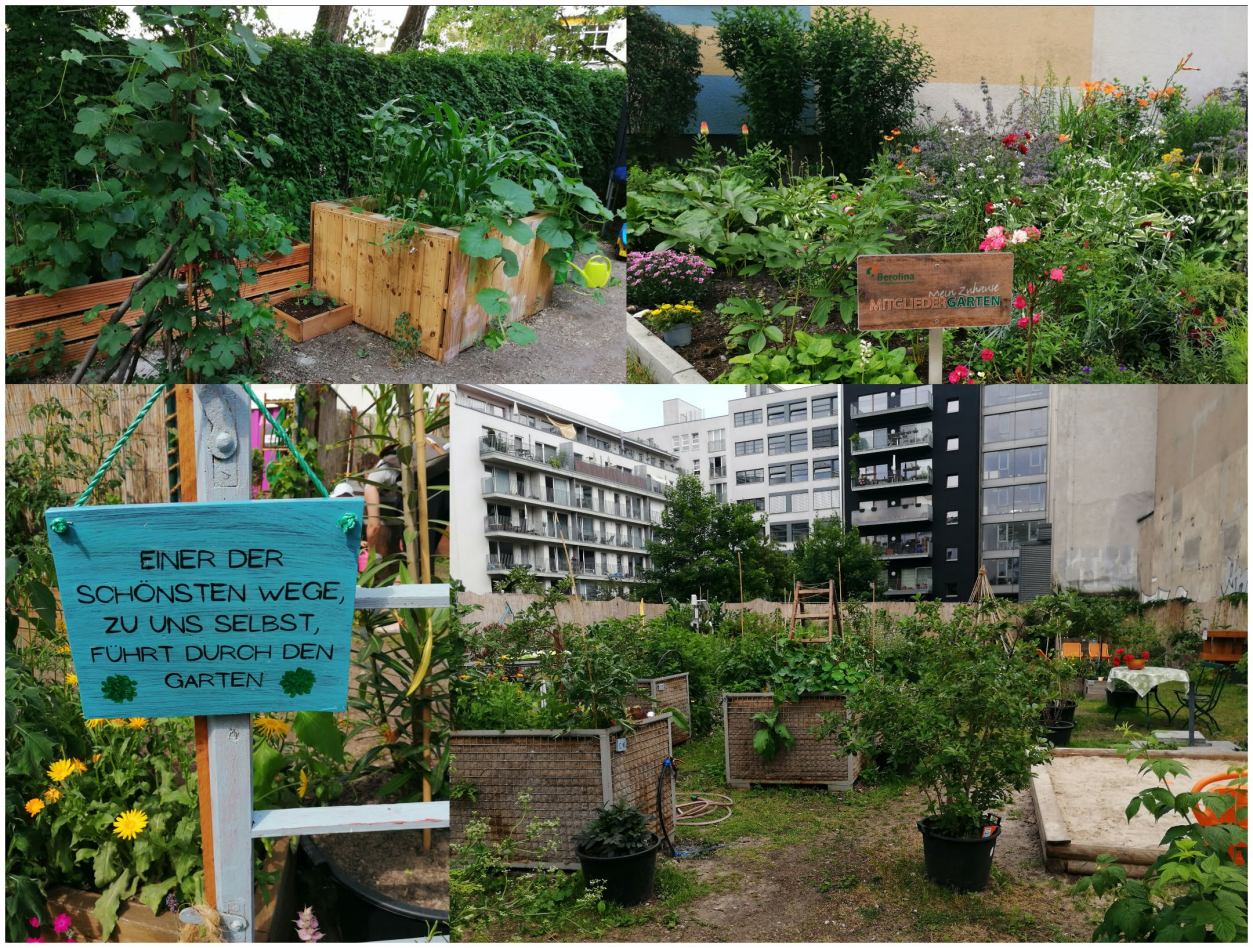
One of the most beautiful paths to ourselves leads through the garden. Tenants and community gardening as crucial element to enhance social cohesion, health and wellbeing in the residential greenery in different building types of Berlin, Germany (Photos: *HealthyLiving*).

Although, inclusive and empowering participation schemes such as co-creation have been proposed to prevent conflicts and counteract green gentrification (60), they are still rare. Moreover, interventions aiming to reduce environmental injustices in fact have also deepened existing ones, failing to effectively and meaningfully involve the affected people in an inclusive manner (61). The urban farming and edible city movement demonstrated that edible nature-based solutions such as vegetable gardens on our doorstep (Figure 8) foster the development of socially inclusive, biodiversity friendly, resilient and healthy cities (62). Thus, community gardens became heterotopias and multilayered places in the post-socialist Zagreb satisfying diverse needs of the residents (63). Community gardens have been also used as strategic tools for neighborhood management by administrations in different Hungarian cities (64) or emerged from bottom-up movements fostering urban commons and food citizenship, e.g., in Cologne (65), Rotterdam (66) or in Berlin (67). Nature-based solutions are implemented using diverse governance arrangements from administration-led to citizens-led modes (38, 68) and extending networks from single productive gardens within a neighborhood to regional scales (35). We provide evidence that the residents of neighborhoods classified as suffering environmental injustice are ready to set aside being “eternal complainers”^2^ about their living environment and become more involved in the design, implementation, and maintenance (Table 1). Consequently, urban planners, neighborhood managers, housing companies and, last but not least, neighbors should join forces to unlock the potential of residential green as an effective measure of preventive medicine (69). While our evaluation of the semi-public open and green spaces attached to multi-story housing complexes (Figures 6, 7) is only a first approximation that needs to be explored in more detail, it demonstrates the enormous development potential that lies on our doorstep and that can be optimized for the benefit of all.

**Figure 9:**
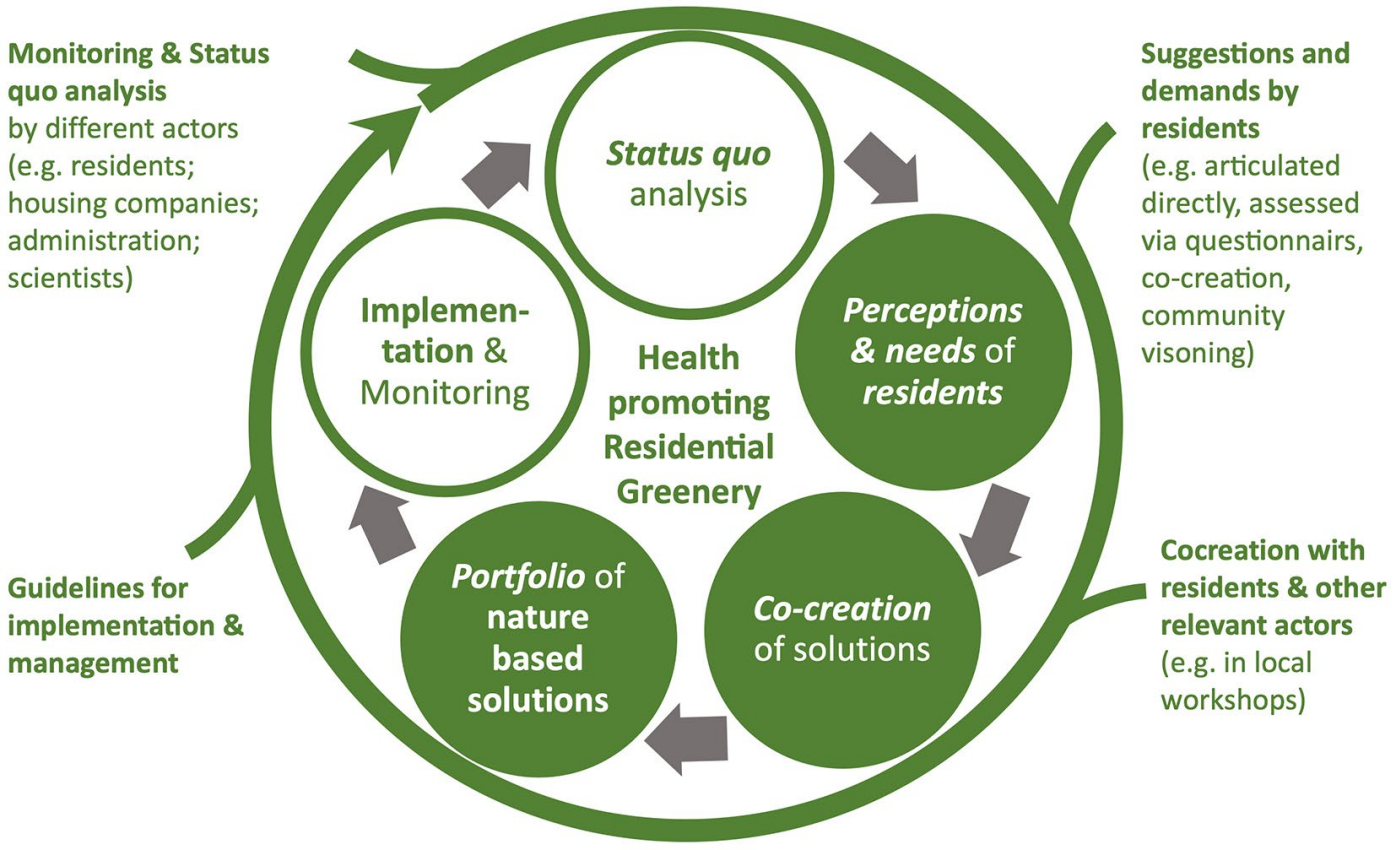
Optimization loop toward health promoting residential greenery.

We have developed a guideline (46), proposed and tested an optimization loop to strategically foster health promoting ecosystem services of residential greenery (Figure 9). The process has different starting points and can be initiated by different actors, e.g., residents can articulate their demands (see Section 2.3) and initiate co-creation processes in a bottom-up manner, especially to discuss conflicting demands and agree on solutions. Guidelines for implementation of nature-based solutions can provide examples and best practices to inspire and inform the process (see Sections 2.4. and 3.3). Our guideline highlights that some interventions are more suitable for certain building structures. As with residents’ suggestions, however, there is no universal solution, even for neighborhoods with similar structural or socioeconomic parameters, because the surrounding urban fabric and its dynamics determine and alter the environmental and social impacts of neighborhoods on different resident groups, and thus on their needs. Neighborhoods and even parts of them, such as blocks or backyards of housing developments, need tailor-made solutions and social-ecological innovations to successfully foster stakeholder engagement, local stewardship and inclusiveness (68, 70, 71). The optimization process can also be initiated by housing companies and local administrations in a top-down manner as a response of identified problems and challenges based on public databases on environmental justice, health or social indicators [e.g., (34, 40)] or in self-research by residents in a bottom-up process.

## 5. Conclusion

Residential greenery is an important and, to date, an under-exploited health resource in the context of the diverse dimensions of individual and public health and wellbeing. Its multifunctionality, accessibility and immediate use by neighbors in everyday life allows it to directly and effectively address hard-to-reach target groups. Our results show that planners and administrators are preaching to the choir that neighbors are already highly motivated to actively participate in the creation of locally adapted solutions and to take responsibility in health-promoting optimization of residential green spaces. Furthermore, there is evidence that biodiversity-friendly interventions are increasingly in demand, supporting planetary health. Designing an inclusive and actively usable “green living room” will not only narrow the gap in times of pandemics and reconnect neighbors as the “social fabric” of our neighborhoods, but also unlock the potential of residential greenery as a “sleeping giant of urban green” that can catalyze biodiversity-friendly urban renewal in quality and quantity. We emphasize the critical role of residential green space in addressing inequities in urban habitat and the need to preserve, restore, and redesign residential green space to improve the health and resilience of our cities.

## Data availability statement

The original contributions presented in the study are included in the article/Supplementary material, further inquiries can be directed to the corresponding author.

## Ethics statement

Ethical review and approval was not required for the study on human participants in accordance with the local legislation and institutional requirements. Written informed consent for participation was not required for this study in accordance with the national legislation and the institutional requirements.

## Author contributions

SM-S developed the guideline. FB developed and analyzed the co-creation workshops. SS and IS performed and analyzed the survey. RR created the maps. IS drafted the first version of the manuscript and received the funding. All authors contributed to the article and approved the submitted version.

## Funding

This research was mainly funded by the Fritz and Hildegard Berg Stiftung in the Deutscher Stifterverband (Germany) in the project “HealthyLiving – Strategie und Planungsinstrument für gesundheitsförderndes Wohnumfeldgrün in der Stadt der Zukunft.” The community garden in Marzahn-Hellersdorf where we develop co-creation experiences is supported as a Living Lab by the European Commission via the Horizon 2020 EdiCitNet project (grant agreement no. 77666). We also thank the Department of Urban and Regional Planning of the Technische Universität for financial support.

## Conflict of interest

The authors declare that the research was conducted in the absence of any commercial or financial relationships that could be construed as a potential conflict of interest.

## Publisher’s note

All claims expressed in this article are solely those of the authors and do not necessarily represent those of their affiliated organizations, or those of the publisher, the editors and the reviewers. Any product that may be evaluated in this article, or claim that may be made by its manufacturer, is not guaranteed or endorsed by the publisher.

## Footnotes

^1^GDR: German Democratic Republic (1949–1990). “Subbotnik” meant doing something for the community on a Saturday (Russian: Subbota). Voluntary work in the housing communities included gardening, care and maintenance of residential greenery. Particularly in the spring, active neighbors worked voluntarily to enhance their residential greenery, sports facilities, playgrounds for schools (e.g., removing garbage, planting trees, shrubs and flowers, painted facades, or renovated club rooms). The housing administration provided materials (e.g., plants, tools, and paint), and people met for a barbecue after work. There was no obligation to participate but it was attractive for neighbors to enhance their social network participating in these activities that at the same time also enhanced the attractivity of the living environment on their doorstep.

^2^The phrase about needing to stop being “perpetual complainers” was often used in the workshops by local stakeholders, including residents, in a very positive sense of being active participants in shaping their neighborhoods rather than remaining in a passive role.
