# Supplementary material for: Awakening the sleeping giant of urban green in times of crisis—coverage, co-creation and practical guidelines for optimizing biodiversity-friendly and health-promoting residential greenery
Source: Front Public Health. 2023 Jun 28;11:1175605. doi: 10.3389/fpubh.2023.1175605 (PMC10345840; doi:10.3389/fpubh.2023.1175605)
Supplement: Supplementary file 1 [file Table_1.DOCX]

***Supplementary Material***

***Awakening the sleeping giant of urban green in times of crisis - Coverage, co-creation and practical guidelines for optimizing biodiversity-friendly and health-promoting residential greenery***

***Sonja Mohr-Stockinger, Simone Sanft, Frederike Büttner, Sylvia Butenschön, Rhea Rennert and Ina Säumel****

**** Correspondence:*** *ina.saeumel@hu-berlin.de*

***
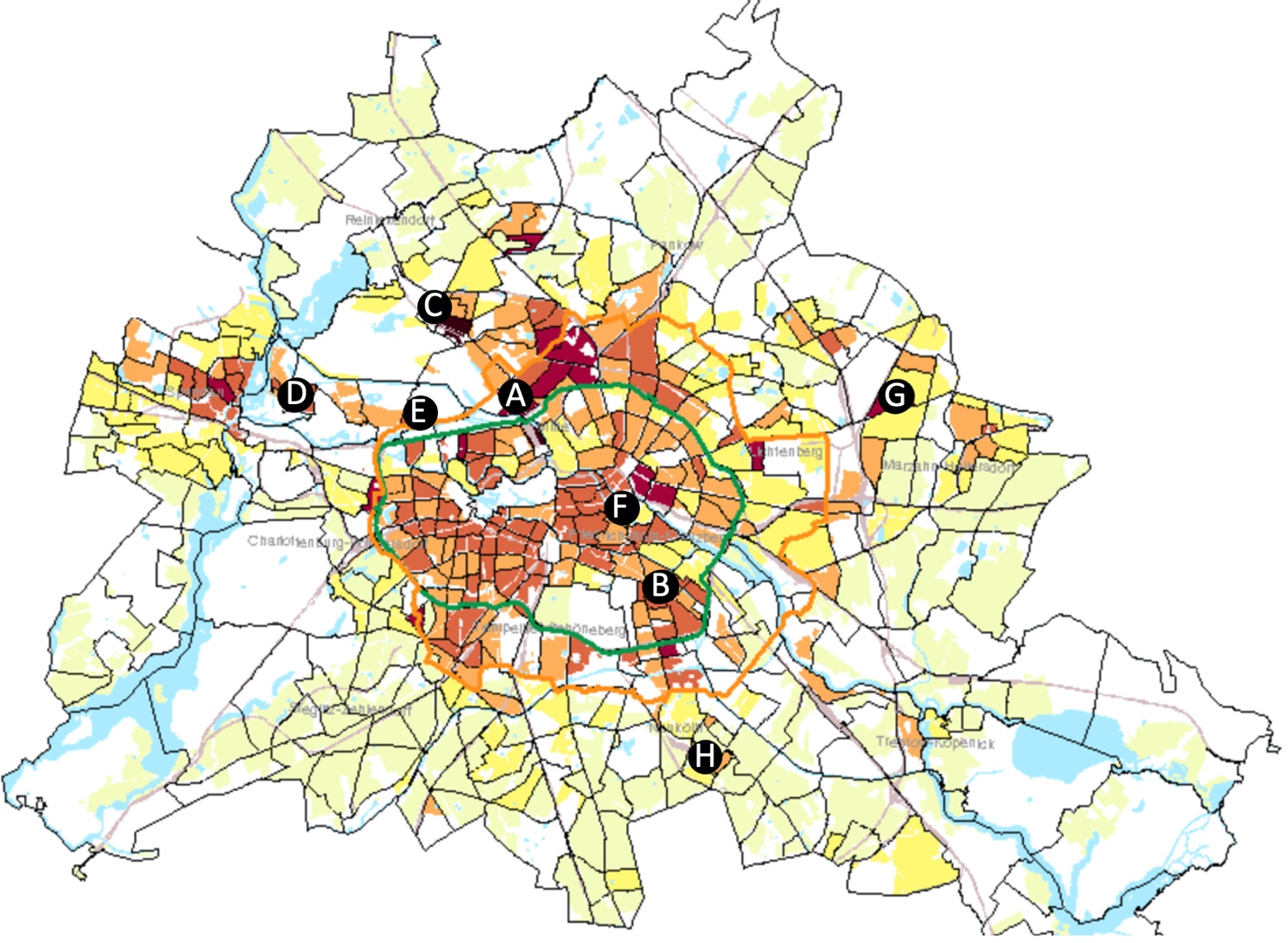
Fig. S1*** *Location of study sites based on Berlin’s map of environmental justice (SenStadtUm 2015): Dense and closed block-edge development from 1870s to 1920s (A: Sprengelkiez, Wedding; B: Ideal-Passage, Neukölln); block-edge development with large green backyards from 1920s to 1940s (C: General Barby Siedlung, Reinickendorf) parallel and free row development with a landscaped residential greenery from 1920s to 1970s (D: Haselhorst, Spandau; E Paul-Hertz-Siedlung, Charlottenburg; F: Alte-Jakobstrasse, Mitte); large housing estates with towers and high rise buildings from 1960s to 1980s (G: Marzahn; H: Gropiusstadt, Neukölln). The map shows multiple exposure to the five core indicators determining environmental (in)-justice (i.e. high noise and air pollution, high bio-climatic stress, low social status indexes and low access to green spaces; see Material and Methods). The darker the color code, the higher the exposure: light green with no exposure to dark brown with exposure to all of the five stressors. Adapted from Fisbroker/Umweltatlas Maps of Berlin (SenStadtUm 2015).*

***Table A.*** *Keywords for classification of different groups among residents regarding suggestions for enhancement of residential greenery*

| *Group* | *Keywords* |
| --- | --- |
| *A: Nature based solutions* | |
| *Green supporters* | *residents that mentioned green nature-based solutions: greening; green (spaces); trees; grass; flowerbeds, lawns; green facades; green roofs; bioswales* |
| *Biodiversity friends* | *residents that mentioned biodiversity aspects of nature based solutions: concrete species and species groups (e.g., bees, june beetles, hedgehogs, insects, conifers, buzzards, bats); biodiversity rich habitats (e.g., meadows, hedges, gardens, dead wood, wilderness); measures to enhance biodiversity friendliness (e.g., protected areas for species, meadows, don't rake, unfold nature, dead wood, insect friendly, biodiversity, biotope, let it grow, bee friendly, nature sounds, ecological education, origin, phenotype)* |
| *Others* | *residents that did not mentioned any needs to enhance residential greenery regarding nature-based solutions* |
| *B: Enhancement of residential greenery* | |
| *Quantitative* | *residents that mentioned quantitative aspects (e.g., more, expand, larger areas, replant, replace trees, don't downsize)* |
| *Qualitative* | *residents that mentioned qualitative aspects (e.g., mowing, maintenance, weeding, watering, too/not drying, drying out, watering, more thoughtfully designed, gardening, caring, designing, gardening, more beautiful)* |
| *Others* | *residents that did not mentioned any needs to enhance residential greenery regarding nature-based solutions* |

***Table B.*** *Number and percentage* *of residents suggesting an enhancement of residential greenery regarding greener and/or biodiverse residential greenery (A) or regarding quantitative and/or qualitative development (B). Some respondents are classified in both categories following the keywords (see Table A). ‘Others’ did not mention any keywords. Comparison between block edge development (Block: Sprengel (A: Sprengelkiez, Wedding), Ideal (B: Ideal-Passage, Neukölln) and Barby (C: General Barby Siedlung, Reinickendorf)), row development (Row: Hasel (D: Haselhorst, Spandau), Hertz (E Paul-Hertz-Siedlung, Charlottenburg) and Jacob (F: Alte-Jakobstrasse, Mitte)) and high-rise buildings (High: Marzahn (G: Marzahn) and Gropius (H: Gropiusstadt, Neukölln)). Chi-squared test, df and p-values are given. For location of study areas see Fig.S1.*

|  |  | *A: Green or biodiverse?* | | | *B: More or better?* | | |
| --- | --- | --- | --- | --- | --- | --- | --- |
|  | *N* | *Green supporters* | *Biodiversity friends* | *Others* | *Quantitative* | *Qualitative* | *Others* |
| *All (N)* | *270* | *106* | *43* | *158* | *56* | *106* | *142* |
| *All (%)* |  | *39* | *16* | *58* | *21* | *39* | *53* |
|  | *N* | *%* | | | | | |
| *Block* | *91* | *34* | *14* | *59* | *21* | *43* | *47* |
| *Row* | *119* | *39* | *18* | *61* | *19* | *39* | *55* |
| *High* | *60* | *47* | *13* | *53* | *23* | *32* | *55* |
| *χ^2^* |  | *2.1* | *0.9* | *0.6* | *0.6* | *1.4* | *0.8* |
| *df* |  | *2* | *2* | *2* | *2* | *2* | *2* |
| *p* |  | *0.341* | *0.627* | *0.740* | *0.736* | *0.442* | *0.665* |
| *Sprengel* | *30* | *33* | *5* | *57* | *27* | *57* | *40* |
| *Ideal* | *30* | *47* | *5* | *53* | *23* | *33* | *47* |
| *Barby* | *30* | *23* | *2* | *70* | *13* | *37* | *57* |
| *Hertz* | *30* | *30* | *1* | *67* | *13* | *37* | *63* |
| *Hasel* | *50* | *32* | *6* | *68* | *10* | *32* | *64* |
| *Jakob* | *40* | *55* | *16* | *45* | *35* | *55* | *38* |
| *Marzahn* | *30* | *47* | *3* | *53* | *33* | *27* | *53* |
| *Gropius* | *30* | *47* | *5* | *53* | *13* | *37* | *57* |
| *χ^2^* |  | *22.1* | *27.9* | *9.8* | *33.2* | *20.8* | *13.0* |
| *df* |  | *7* | *7* | *7* | *7* | *7* | *7* |
| *p* |  | *0.002* | *<0.001* | *0.201* | *<0.001* | *0.004* | *0.072* |

***Table C.*** *Clustered notes of the co-creation workshops regarding the different topics (A) Envision a successful participation process for redesigning residential greenery; (B) What does your residential greenery ideally look like?; (C) How can contrasting demands coexist? The given answers were clustered into six different categories (see below). The table shows the absolute number of allocations per cluster and the percentage rate in brackets.*

| *Clusters* | *A* | *B* | *C* |
| --- | --- | --- | --- |
| *(1) Communication and Information (e.g., mentioning measures to inform and dialogue with neighbors such as newsletter, notice board, flyer, meetings, workshops; support by expert knowledge)* | *10 (45)* | *0 (0)* | *5 (15)* |
| *(2) Coordination of the process and contact person (e.g., support in moderation and facilitation of the participation process and design; technical support; contact person)* | *4 (18)* | *6 (19)* | *8 (25)* |
| *(3) Demands on the residential greenery and needs (e.g., different functions and needs, garden plots, play grounds, benches)* | *0 (0)* | *6 (19)* | *12 (36)* |
| *(4) Responsibilities (e.g., volunteering, workshops, Subbotniks, participation, integration in rental contracts)* | *7 (32)* | *9 (28)* | *6 (18)* |
| *(5) Biodiversity (e.g., wildflowers, nature near vegetation, meadows, pollinators, fruit trees)* | *0 (0)* | *7 (21)* | *0 (0)* |
| *(6) Concerns (e.g., conflicts of interests, open dialogue, involvement, barriers, threats, anonymity)* | *1 (5)* | *4 (13)* | *2 (6)* |
| *Total number of answers* | *22* | *32* | *33* |

**Evidence for health-relevant ecosystem services of nature-based solutions (NBS) for residential greenery**

*Green roofs and façade greening.* Both façade and roof greening (Fig.S2) are building-attached or integrated nature-based solutions that provide a wide range of health relevant ecosystem services such as climate regulation (Francis and Lorimer 2011; Oberndorfer et al. 2007; Francis 2011) or enhancing air quality by lowering particular matter loads (Rowe 2011; Viecco et al. 2021). Façade greening can reduce urban heating effects especially in street canyons where people stay and walk (Matzinger et al. 2017) and, depending on the construction, can also reduce heating costs in winter (Tudiwer and Korjenic 2017). As a result, the buildings are exposed to lower temperature fluctuations, which are particularly difficult for city dwellers. Particularly in densely populated areas, where little free space is available, roofs become attractive as unused space, as a simple green roof or as an intensive roof garden. Even simple elements can create green oases from small areas such as shed or garage roofs. In general, as a pleasant window view on nature can promote health and wellbeing (Raanaas et al. 2012; Schmid and Säumel 2021; Løkken Nordrum 2022), visibility of building attached green is crucial for health and wellbeing. Where a complete roof cannot be planted with greenery, raised beds can be installed and planted according to the wishes of the residents. In addition, benches, tables and individual play elements create a private recreation area above the roofs of the big city. Depending on the initial situation, individual raised beds can also become roof gardens, leading to less load for the roofs and offering the inhabitants more opportunities to implement their own design ideas. Adding biochar makes green roof substrates lighter, improves plant water supply and stormwater retention (Cao et al. 2014). In façade greening, distinction is made between ground-based and wall-based systems. Ground-based systems have the advantage of requiring less construction and, depending on the type of planting, require less maintenance. As they require little space, they can also be used well in small open spaces. Ground-based systems are irrigated exclusively via the root system in the ground. Wall-bound systems require higher technical demands because the plants are not rooted on the ground but in wall constructions, so continuous artificial irrigation must be guaranteed. Due to the construction and maintenance costs for wall-mounted systems, the costs of facade greening are significantly higher. In contrast to ground-based systems, the selection of plants is more diverse, and the insulating/ cooling effect increases through evaporation. In addition, green roofs and façade greening reduce urban noise pollution (van Renterghem and Botteldooren 2009; Rowe 2011). In street canyons in particular, traffic noise penetrates the interior of the building. There, depending on leaf thickness, leaf size and leaf position, soft vegetation layer can create a buffering effect. Reduced pollution caused by fine dust in buildings exposed to the street is also worth mentioning. In areas of low air flow, façade and roof greening are expected to restrain up to 40% of air pollutants (Pugh et al. 2012).


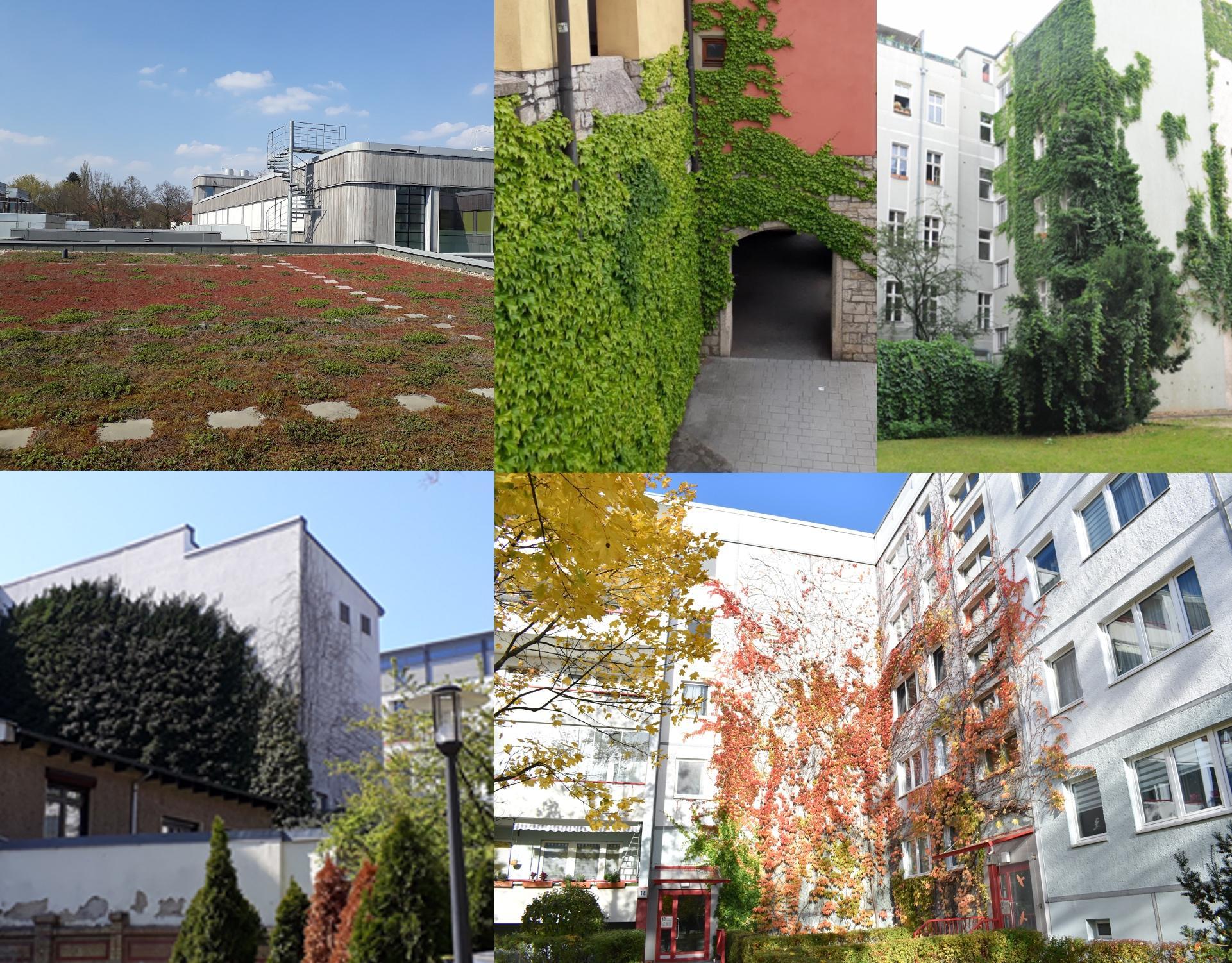


**Fig.S2** Green façades and green roof as an element of residential greenery in different building types of Berlin, Germany (Photos: *HealthyLiving*)

*Meadows, wild shrubs and lawns***.** Both active and passive usage of green elements benefits human mental and physiological health (Jennings et al. 2016). Green spaces such as lawns enhance physical activity and contribute to socio-cultural gathering (Zhou and Rana 2012). In particular, mobility-impaired groups, such as elderly, disabled or sick residents, who spend most of the daytime in the residential area, benefit from moderate physical activity such as walks in the residential green spaces (Takano et al. 2002). Visible plantation of perennial wildflower meadows enhances the aesthetic value and contribute to cultural services (White and Gatersleben 2011). In small spaces, wild perennials such as yarrow, meadow sage or lavender can easily be planted in raised beds or tubs and placed in sun-exposed areas of a courtyard (Fig.S3). This alternative is extremely space-saving and an open floor is not a requirement. In sealed courtyards, they help to upgrade the existing space. Spontaneous vegetation beyond street trees provides health relevant ecosystem services such as immobilization of particular matter (Weber et al. 2014b; Säumel et al. 2016) and are appreciated by urban dwellers (Weber et al. 2014a). Playgrounds in natural surroundings are of particular importance in the inner-city center. Interacting with nature supports intellectual development and improves affect (Bratman et al. 2015). Areas close to nature in particular offer space for imaginative design of the environment, fulfilling children's urge to discover, and reducing symptoms such as concentration difficulties, aggression, and impulsive behavior (Kuo and Taylor, 2004).


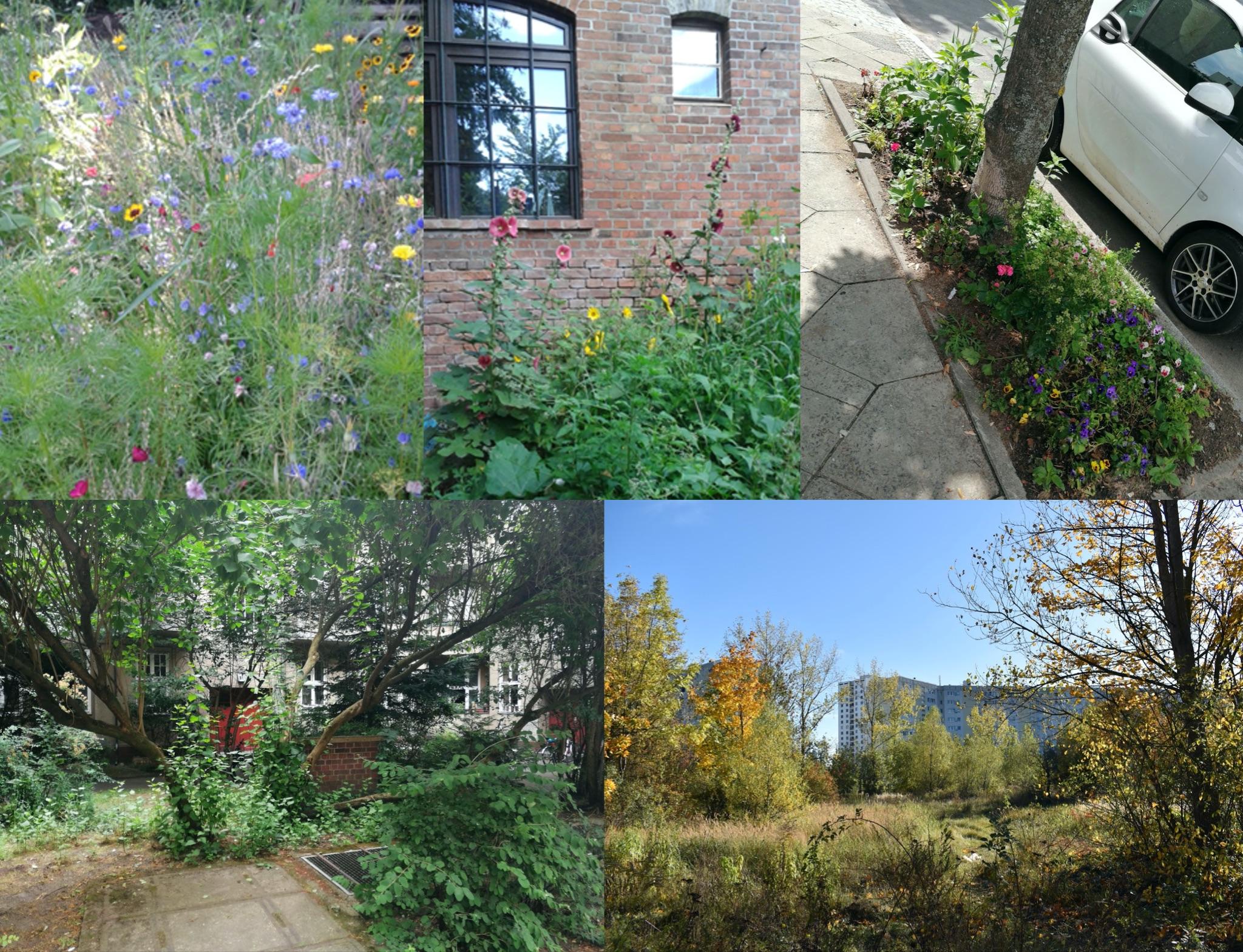


**Fig.S3** Wilderness elements of residential greenery in different building types of Berlin, Germany (Photos: *HealthyLiving*)

*Bodies of water.* While a pond is one of the most complex measures for redesigning the living environment, it also quickly becomes the undisputed social and ecological focal point of any garden area (Pille and Säumel 2021). Passive cooling of urban residential areas can be enhanced through ponds and wells due to evapotranspiration (Manteghi et al. 2015). If parts of the pond are framed with perennials and shrubs, retreats and corridors are created for small animals. Seating allows observation by residents. Alternatively, a simple water source in the form of larger bird baths can develop a similar attraction for residents and animals, and are vital for birds, insects and small mammals, especially in inner-city areas, where the degree of sealing is high and freely accessible bodies of water are rare.


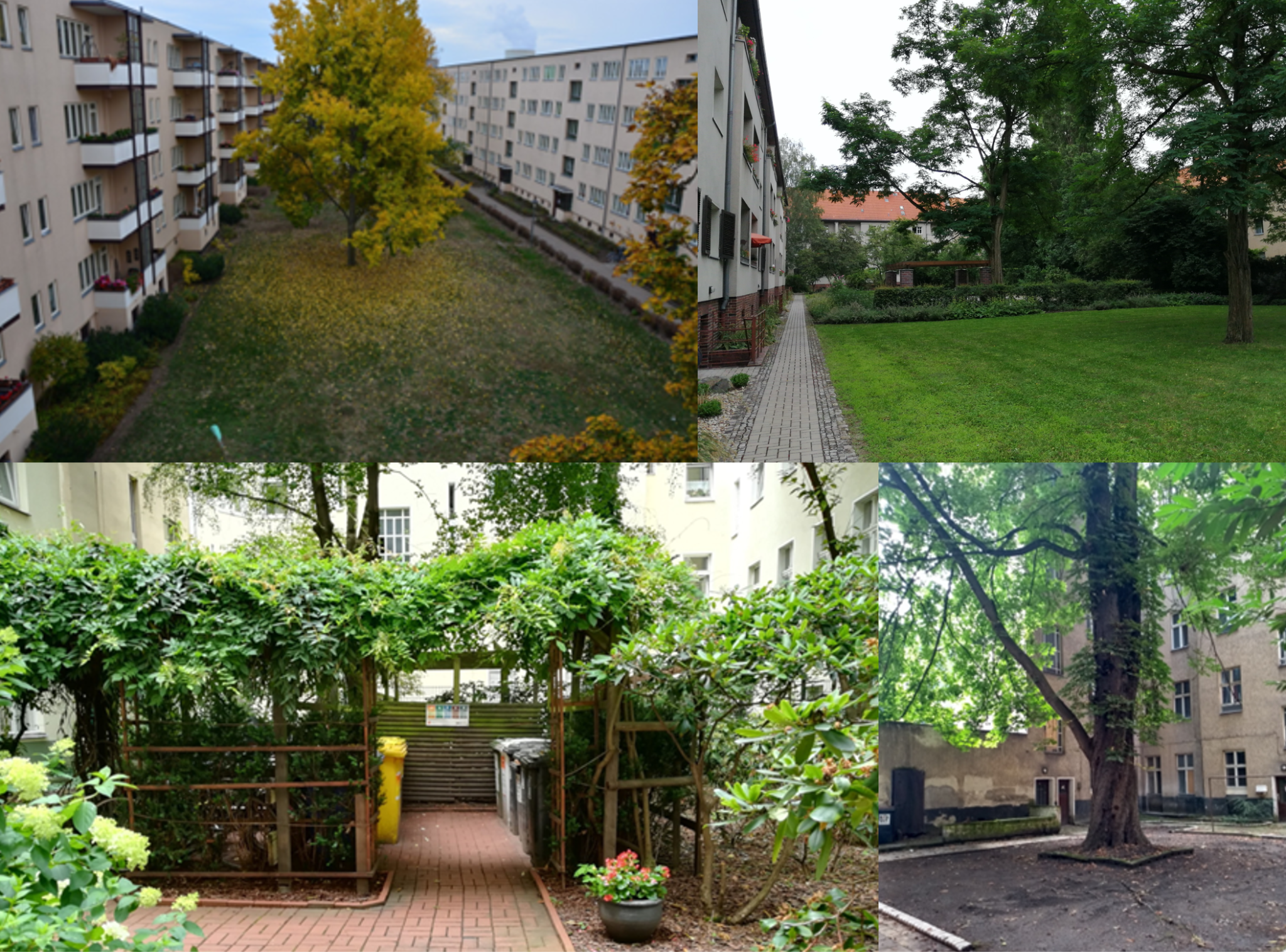


**Fig.S4** Woody species as elements of the residential greenery including trees, shrubs and hedges in different building structure types of Berlin, Germany (Photos: *HealthyLiving*).

*Woody plantations, shrubs and hedges.* Deciduous and coniferous trees (Fig.S4) can significantly contribute to offsetting thermal stress. Shading through tree pits reduces the surface temperature in inner courtyards up to 10 degrees Celsius, while simultaneously reducing energy use for cooling (Akbari et al. 2001). Foliage in the growing season reduces the amount of solar radiation reaching sealed ground and buildings and the associated heating of the environment. At the same time, the air is cooled by transpiration. Moreover, homes located near busy roads also benefit from a slight noise reduction, as well as improved air quality through filtration of air pollutants (Weber et al. 2014b; Salmond et al. 2016). Shrubs and hedges smaller shrubs can be planted in tubs and beautify an inner courtyard or peripheral areas as a substitute for trees. In contrast to trees, the shading rate is low, which means that even in small spaces and close to buildings, plants can be planted without the foliage casting too much shadow on the adjacent apartments during the growing season. Shrubs can be used as structuring elements in open spaces.

*Common principles of green regeneration.* Some common ecological design principles can be derived for green regeneration across the different building structures (Pickett and Cadenasso 2008; Pille and Säumel 2021; Mestre et al. 2021): 1) *Unseal urban soil.* Urban soils show an extreme degree of sealing (Fig.7 in the main text). Since the soil, as a storage and buffer both for water and to compensate for temperature fluctuations, can only fulfill its natural function if it is not sealed, the top priority of the green transformation is its unsealing. Open ground provides the basis for all design measures, creating space for plants and animals to settle and significantly improving the local microclimate (Tobias et al. 2018). 2) *Choosing native plant species.* When designing near-natural areas, a decision must first be made as to whether this is a temporary or permanent measure. When choosing plants, native flora, so a large selection of plants most adapted to climatic conditions, should be preferred. This will also enhance the livelihood of many native insects and animal species, making ecological communities possible (de Carvalho et al. 2022). 3) *Ecological and structural diversity.* Diversity of plants and structures create an equally large variety of habitats, which are essential for stable ecosystems (Hunter 2011; Pille and Säumel 2021). Overgrown areas offer retreats for small animals such as hedgehogs, lizards and birds and improve health and wellbeing via nature relatedness (Dean et al. 2018). 4) *Building materials.* There is growing evidence for a healthy, safe and sustainable housing (D’Alessandro et al. 2020; Foster et al. 2022) covering also building materials and layout including biomaterials and biodesign in architecture (Andréen and Goidea, 2022) and multi-species approaches (de Wilde and Bleil de Souza, 2022, Selvan et al. 2023). New elements in the residential environment consist primarily of wood, natural stone or gravel, biological engineering construction methods (Li et al. 2016) and healthy materials (Petrović et al. 2017).

*Literature:*

Akbari, H., Pomerantz, M., and Taha, H. (2001). Cool surfaces and shade trees to reduce energy use and improve air quality in urban areas. Sol Energy 70(3):295–310. doi:10.1016/S0038-092X(00)00089-X.

Andréen, D., and Goidea, A. (2022). Principles of biological design as a model for biodesign and biofabrication in architecture. Archit. Struct. Constr. 2:481–491. doi:10.1007/s44150-022-00049-6.

Bratman, G. N., Daily, G. C., Levy, B. J., and Gross, J.J. (2015). The benefits of nature experience: Improved affect and cognition. Landsc. Urban Plan. 138:41–50. doi:10.1016/j.landurbplan.2015.02.005.

Cao, C.T.N., Farrell, C., Kristiansen, P.E., and Rayner, J.P. (2014). Biochar makes green roof substrates lighter and improves water supply to plants. Ecol. Eng. 71:368-374. doi: 10.1016/j.ecoleng.2014.06.017.

D'Alessandro, D., Gola, M., Appolloni, L., Dettori, M., Fara, G.M., Rebecchi, A., Settimo, G., and Capolongo, S. (2020). COVID-19 and Living space challenge. Well-being and Public Health recommendations for a healthy, safe, and sustainable housing. Acta Biomed. 91(9-S):61-75. doi: 10.23750/abm.v91i9-S.10115.

de Carvalho, C.A., Raposo, M., Pinto-Gomes, C., and Matos, R. (2022). Native or Exotic: A Bibliographical Review of the Debate on Ecological Science Methodologies: Valuable Lessons for Urban Green Space Design. Land 11:1201. https:// doi.org/10.3390/land11081201.

de Wilde, P., and Bleil de Souza, C. (2022). Interactions between buildings, building stakeholders and animals: A scoping review. J. Clean. Prod. 367: 133055. doi: 10.1016/j.jclepro.2022.133055.

Dean, J.H., Shanahan, D.F., Bush, R., Gaston, K.J., Lin, B.B., Barber, E., Franco, L., and Fuller, R.A. (2018). Is Nature Relatedness Associated with Better Mental and Physical Health? Int J Environ Res Public Health. 15(7):1371. doi: 10.3390/ijerph15071371.

Foster, S., Hooper, P., Turrell, G., Maitland, C., Giles-Corti, B., and Kleeman, A. (2022). Grand designs for design policy: Associations between apartment policy standards, perceptions of good design and mental wellbeing. SSM Popul Health. 20:101301. doi: 10.1016/j.ssmph.2022.101301.

Francis, R.A. (2011). Wall ecology: a frontier for urban biodiversity and ecological engineering. Prog Phys Geogr. 35(1):43-63. https://doi.org/10.1177/0309133310385166.

Francis, R.A., and Lorimer, J. (2011). Urban reconciliation ecology: the potential of living roofs and walls. J. Environ. Manage. 92(6):1429-1437. doi: 10.1016/j.jenvman.2011.01.012.

Hunter M.C. (2011). Using Ecological Theory to Guide Urban Planting Design. Landsc. J. 30(2):173-193 doi: 10.3368/lj.30.2.173.

Jennings, V., Larson, L., and Yun, J. (2016). Advancing Sustainability through Urban Green Space: Cultural Ecosystem Services, Equity, and Social Determinants of Health. Int. J. Environ. Res. Public Health. 13(2):196. doi:10.3390/ijerph13020196.

Kuo, F. E., and Taylor, A. F. (2004). A potential natural treatment for attention-deficit/hyperactivity disorder: evidence from a national study. Am. J. Public Health 94(9):1580–1586. doi:10.2105/ajph.94.9.1580.

Li, Y., Wang, Y., Ma, C., Zhang, H., Wang, Y., Song, S., and Zhu, J. (2016). Influence of the spatial layout of plant roots on slope stability. Ecol. Eng. 91: 477-486. doi: 10.1016/j.ecoleng.2016.02.026.

Løkken Nordrum, O. (2022). Health care with a view. Lancet Planet. Health. doi: 10.1016/S2542-5196(22)00170-X.

Manteghi, G., Limit, H. B., and Remaz, D. (2015) Water Bodies an Urban Microclimate: A Review. MAS 9(6). doi:10.5539/mas.v9n6p1.

Matzinger, A., Riechel, M., Remy, C., Schwarzmüller, H., Rouault, P., Schmidt, M., Offermann, M., Strehl, C., Nickel, D., Sieker, H., Pallasch, M., Köhler, M., Kaiser, D., Möller, C., Büter, B., Leßmann, D., von Tils, R., Säumel, I., Pille, L., Winkler, A., Bartel, H., Heise, S., Heinzmann, B.; Joswig, K., Rehfeld-Klein, M., and Reichmann, B. (2017). Zielorientierte Planung von Maßnahmen der Regenwasserbewirtschaftung - Ergebnisse des Projektes KURAS. Kompetenzzentrum Wasser Berlin gGmbH. Available at: http://kuras-projekt.de/downloads/erzeugnisse-regenwasserbewirtschaftung/

Mestre, N., Roig, E., and Almestar, M. (2021). Beyond Nature-Based Rhetorics: A Prospect on the Potentials of Redundancy in Ecology-Oriented Design. Sustainability 13: 13293. doi: 10.3390/su132313293.

Oberndorfer, E., Lundholm, J., Bass, B., Coffman, R.R. Doshi, H. Dunnett N., Gaffin, S., Köhler, M., Liu, K.K.Y., and Rowe, B. (2007). Green roofs as urban ecosystems: ecological structures, functions, and services. Bioscience 57(10):823-833. doi: [10.1641/B571005](https://doi.org/10.1641/B571005).

Petrović, E.K., Vale, B., and Zari, M.P. (2017). Materials for a Healthy, Ecological and Sustainable Built Environment: Principles for Evaluation. Woodhead: Elsevier.

Pickett S., and Cadenasso, M. (2008). Linking ecological and built components of urban mosaics: an open cycle of ecological design. J. Ecol. 96:8-12. doi: 10.1111/j.1365-2745.2007.01310.x.

Pille, L., and Säumel, I. (2021). The water-sensitive city meets biodiversity: habitat services of rain water management measures in highly urbanized landscapes. Ecol. Soc. 26(2):23. doi: 10.5751/ES-12386-260223.

Pugh, T.A.M., MacKenzie, A.R., Whyatt, J.D., and Hewitt, C.N. (2012). Effectiveness of green infrastructure for improvement of air quality in urban street canyons. Environ. Sci. Technol. 46 (14):7692–7699. doi:10.1021/es300826w.

Raanaas, R.K., Grindal Patil, G., and Hartig, T. (2012). Health benefits of a view of nature through the window: a quasi-experimental study of patients in a residential rehabilitation center. Clin. Rehabil. 26(1):21-32. doi: 10.1177/0269215511412800.

Rowe, D.B. (2011). Green roofs as a means of pollution abatement. Environ. Pollut. 159(8–9): 2100-2110. doi: h10.1016/j.envpol.2010.10.029.

Salmond, J.A., Tadaki, M., Vardoulakis, S., Arbuthnott, K., Coutts, A., Demuzere, M., Dirks, K.N., Heaviside, C., Lim, S., Macintyre, H., McInnes, R.N., and Wheeler, B.W. (2016). Health and climate related ecosystem services provided by street trees in the urban environment. Environ Health. 8,15 (1):36. doi: 10.1186/s12940-016-0103-6.

Säumel, I., Weber, F., and Kowarik, I. (2016). Toward livable and healthy urban streets: roadside vegetation provides ecosystem services where people live and move. Environmental Science and Policy. doi: 10.1016/j.envsci.2015.11.012.

Schmid, H.L., and Säumel, I. (2021). Outlook and Insights: Perception of residential greenery in multi storey housing estates in Berlin, Germany. Urban For. Urban Green. 63:127231. doi: 10.1016/j.ufug.2021.127231.

Selvan, S.A., Saroglou, S.T., Joschinski, J., Calbi, M., Vogler, V., Barath, S., and Grobman, Y.J. (2023). Toward multi-species building envelopes: A critical literature review of multi-criteria decision-making for design support. Build. Environ. 231:110006, doi: 10.1016/j.buildenv.2023.110006.

Takano, T., Nakamura, K., and Watanabe, M. (2002). Urban residential environments and senior citizens’ longevity in megacity areas: the importance of walkable green spaces. J. Epidemiology Community Health 56(12):913–918. doi: 10.1136/jech.56.12.913.

Tobias, S., Conen, F., Duss, A., Wenzel, L.M., Buser, C., and Alewell, C. (2018). Soil sealing and unsealing: State of the art and examples. Land Degrad. Dev. 29: 2015-2024. doi: 10.1002/ldr.2919.

Tudiwer, D., and Korjenic, A. (2017). The effect of living wall systems on the thermal resistance of the façade. Energ. Buildings 135:10–19. doi: 10.1016/j.enbuild.2016.11.023.

Van Renterghem, T., and Botteldooren, D. (2009). Reducing the acoustical façade load from road traffic with green roofs. Build. Environ. 44 (5):1081–1087. doi: 10.1016/j.buildenv.2008.07.013.

Viecco, M. Jorquera, H., Sharma, A., Bustamante, W., Fernando, H.J., and Vera, S. (2021). Green roofs and green walls layouts for improved urban air quality by mitigating particulate matter. Build. Environ. 204108120. doi: 10.1016/j.buildenv.2021.108120.

Weber, F., Kowarik, I., and Säumel, I. (2014a). A walk on the wild side: perceptions of roadside vegetation beyond trees. Urban For. Urban Green. 13: 205-212. doi: 10.1016/j.ufug.2013.10.010.

Weber, F., Kowarik, I., and Säumel, I. (2014b). Herbaceous plants as filters: Immobilization of particulates along urban street corridors. Environ. Pollut. 186: 234–240. doi: 10.1016/j.envpol.2013.12.011.

White, E. V., and Gatersleben, B. (2011). Greenery on residential buildings: Does it affect preferences and perceptions of beauty? J. Environ. Psychol. 31 (1):89–98. doi: 10.1016/j.jenvp.2010.11.002.

Zhou, X., and Rana, Md. M. P. (2012). Social benefits of urban green space. Manag. Environ. Qual. 23(2):173–189. doi: 10.1108/14777831211204921.
