# Supplementary material for: Awakening the sleeping giant of urban green in times of crisis—coverage, co-creation and practical guidelines for optimizing biodiversity-friendly and health-promoting residential greenery
Source: Front Public Health. 2023 Jun 28;11:1175605. doi: 10.3389/fpubh.2023.1175605 (PMC10345840; doi:10.3389/fpubh.2023.1175605)
Supplement: Supplementary file 2 [file Data_Sheet_1.PDF]

Health-oriented redesign of the inner-city residential greenery

Sonja Mohr-Stockinger

Guideline for Housing Cooperatives and Companies

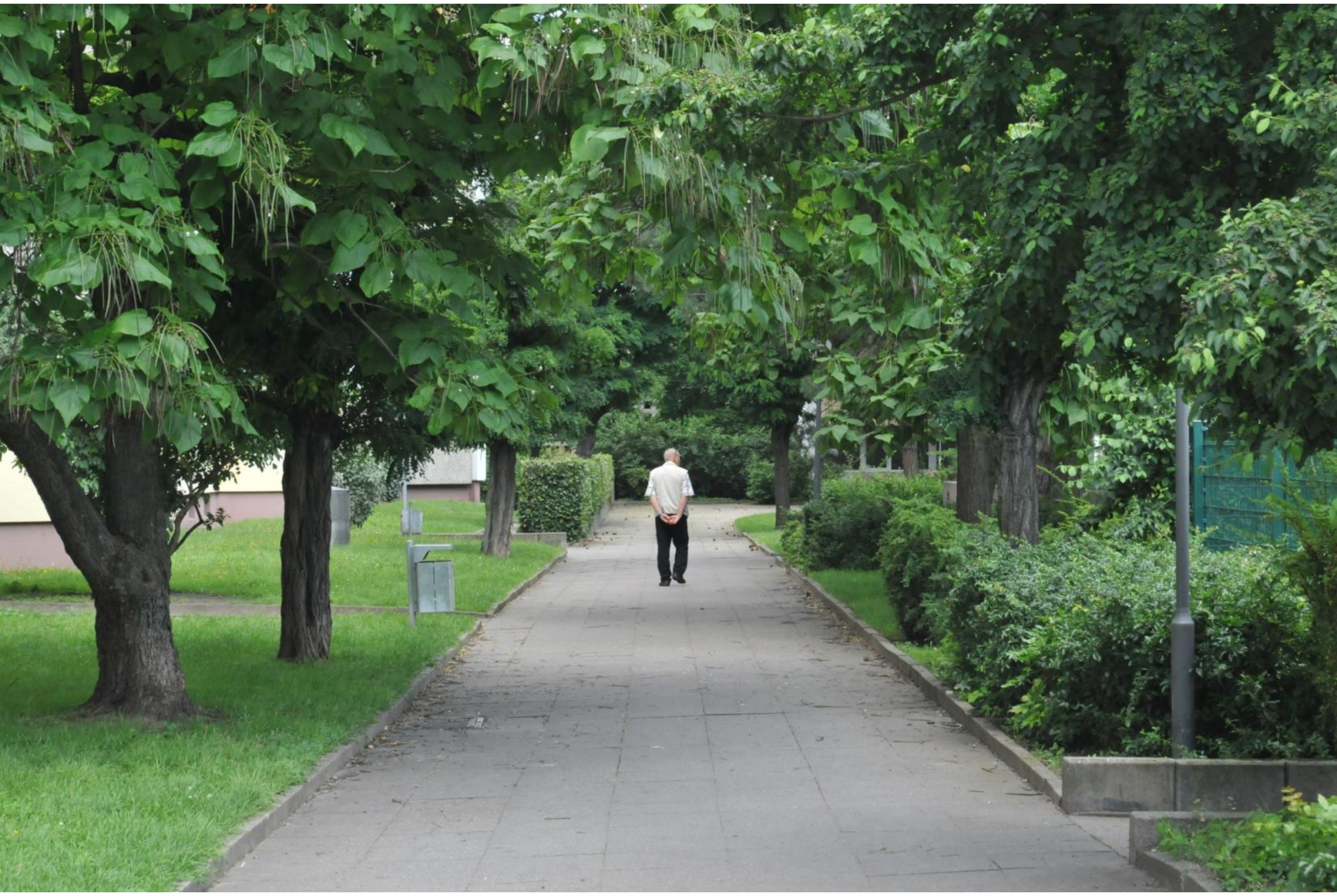

HealthyLiving
Strategie und Planungsinstrument für gesundheitsförderndes Wohnumfeldgrün in der Stadt der Zukunft

IRI THESys

Humboldt-Universität zu Berlin

Technische Universität Berlin

Foreword

The guide “Health-oriented redesign of the inner-city residential greenery” was created as part of the *HealthyLiving* project at the Technical University of Berlin. It is part of a master's thesis in the "Urban Ecology" course.
HealthyLiving deals with strategies of sustainable urban planning, considering the balancing and relief effect of inner-city green and blue structures on the quality of life and health of the city dwellers. *HealthyLiving* is funded by the Fritz and Hildegard Berg Foundation in the Stifterverband für die Deutsche Wissenschaft and the framework program FONA (Research for Sustainable Development) of the BMFB.

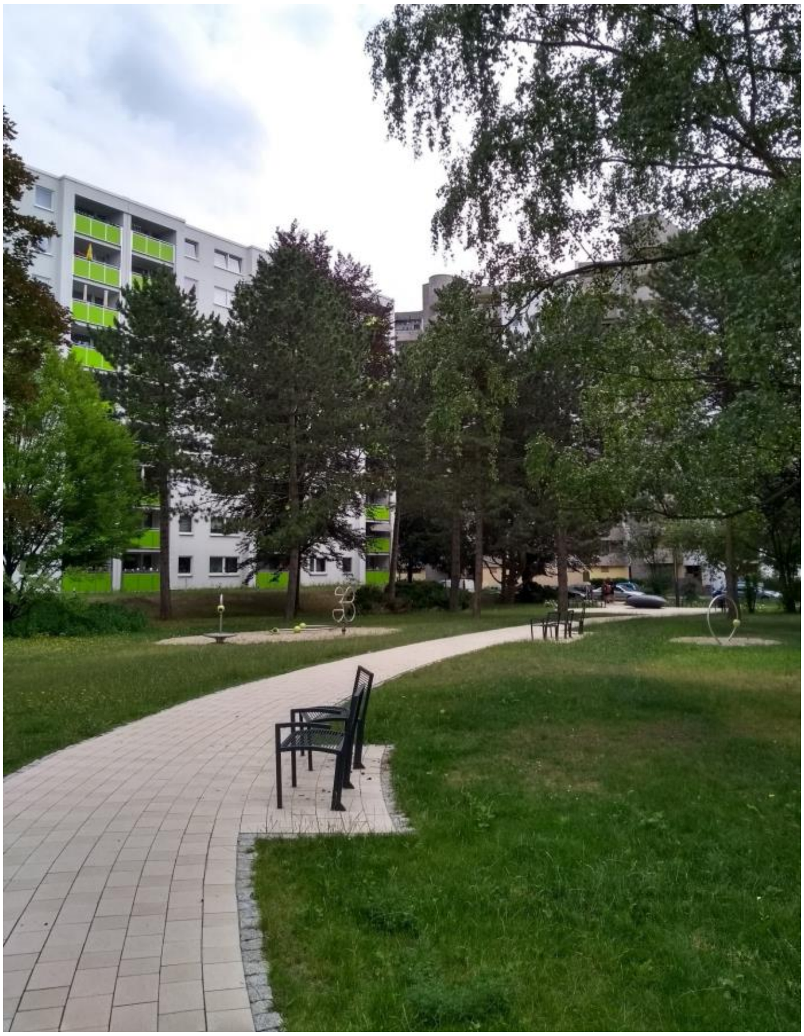

Table of Contents

1 Urban nature
the city and the urban climate
urban loads
urban green and health
HealthyLiving project

2 Green transformation
method
city structure types
red thread of the green transformation

3 Practical implementation
roof greening
facade greening
meadows and wild shrubs
actively design laws
bodies of water
woody plantations
shrubs and hedges
paths, parking lots and peripheral areas

4 Imprint

5 Decision matrix

1 Urban nature

The city and its climate

City and nature are two terms that describe a conflict in the minds of many people:
Where the city develops, nature has no place.
From today's perspective, this supposed contradiction is outdated and a relic from the time when many stretches of land and semi-natural terrain had to give way to built structures. The need for living space close to work increased in times of industrialization, and thus cities increasingly became the new place of residence for working society.
Cities around the world have always been growing: according to the World Health Organization (WHO), more than 50% of the world's population currently lives in cities - a trend that is unstoppable.
The increasing population density presents cities with completely new challenges: new living space must be created and at the same time designed in such a way that the quality of life of the city dwellers is not impaired.
In contrast to the near-natural environment, the urban habitat has properties that can limit the health of the urban population.

In structurally weak areas in particular, the socially disadvantaged population is currently still exposed to multiple burdens, which has led to inequality in terms of environmental justice (see: Environmental Atlas Berlin).

How can future-oriented cities emerge that focus on climate adaptation and the health of its people? How can a city become more environmentally friendly and improve the quality of life for all citizens?

Within the framework of this guide, the urban climate and its characteristics are presented and measures that deliver health-oriented ecosystem services are explained.

The aim is to give a practice-oriented overview of the complex issue of urban nature and its influence on human well-being.

Urban loads

Cities have a high proportion of built structures. Stone, steel and concrete cool down in winter, but heat up drastically in summer. This sometimes leads to large temperature differences between the city center and the surrounding area. Sealed floors also impede natural heat regulation due to the lack of evaporation. In particular, the lack of cooling at night has turned cities into so-called heat islands. Heat islands are a major burden for the human organism. In summer heat waves, mortality increases primarily in cities.

If one considers the rising average temperatures due to climate change, it becomes clear that the urban climate urgently needs to be adapted to protect the urban population.

However, not only structural structures have an influence on the local climate. Traffic and industry lead to increased particulate matter pollution and poor air quality. While the finest particles in the air settle in plant structures and are filtered, these particles linger in high concentrations between dense buildings and busy roads if there is no vegetation.

Another branch of research is currently dealing with the effects of artificial lighting. The street scene at night has changed significantly, particularly using LEDs as a climate-friendly alternative to light bulbs. Today the light pollution "sky glow" is recognized as an important factor of human health.

1 Urban nature

Urban green and health

In order to redesign a city in a future-oriented manner, it is worth taking a look at nature in order to recognize and use its health potential for reducing various urban influences such as noise and air quality.

It is not only the stresses mentioned above that lead to a reduction in well-being, it is often also the living situation in the city that is significantly involved in the state of health of the city dwellers.
The positive contributions that urban greenery offers are presented in the concept of ecosystem services.

Ecosystem services are generally defined as the indirect and direct contributions that nature provides to human well-being. The contributions that are of importance in the urban context can be classified primarily into three categories of regulatory, cultural and supply services.
Regulatory services lead to a reduction in pollution of the urban climate, the air and the water.
Supply services include the drinking water supply and the provision of raw materials such as wood and food from urban cultivation.
Cultural services, on the other hand, describe the recreational and educational benefits that society draws from urban nature.

If ecological functions of urban nature are of use to a society, they receive the title of ecosystem service.
In this context, it is important to mention that the use of ecosystem services can vary depending on cultural understanding and understanding of values and can be used differently in different societies or groups.

The ecosystem services can be read in detail in the "TEEB Natural Capital Germany".

For a successful implementation of urban green in existing structures, it is necessary to name the possibilities and limits. Before the individual greening measures are described below, the building structure is analyzed. The basis is provided by the data from the Berlin Senate Administration, on which the categorization of the *HealthyLiving* project is based.

This results in roughly 4 categories into which the majority of Berlin apartment buildings can be classified.
In order to make it easier to decide on one of the greening measures described, the measures and building structure types are shown graphically in a matrix in order to identify potential and to be able to propose and evaluate alternatives.

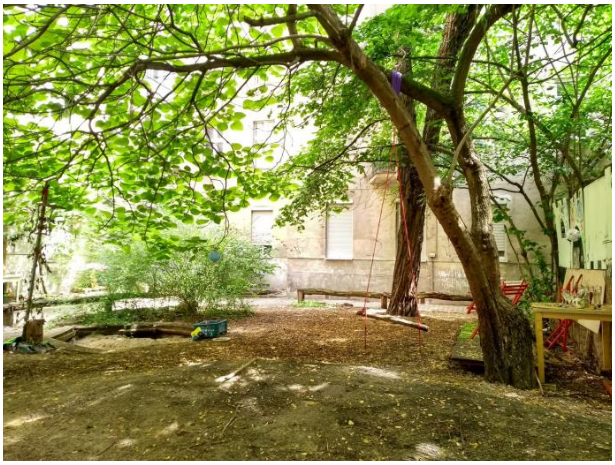

2 Green transformation

Method

Before the practical implementation of the project can begin and planting can start, strategic planning is required in advance. In this way, the financial and time expenditure can be estimated, and dead ends avoided. At the same time, all those involved are involved in the decision-making process at every planning stage and can express their own wishes, ideas and concerns.

1. Inventory analysis - What is the status quo?

In a first step, the initial situation is analyzed. The following questions must be answered:

- What type of urban structure is there that needs to be redesigned? (See the description of the city structure types)
- What possibilities and limitations arise from the respective structures? Which circumstances limit the implementation, what are the advantages for a green transformation?
- Are there open spaces, if yes: in which formation - contiguous or partial?
- What is the soil condition, how high is the degree of sealing?

2. Mobilize residents - live participation!

- What are the wishes and needs of local residents? Intercultural competence is an advantage here. The measures must also be tailored to the age structure of the residents (seating, playgrounds, etc.)
- Reveal deficiencies, are there concerns? (Allergenic plantings, poisonous shrubs, etc.)
- Exploit opportunities for participation. (See: urban gardening, green roofs, etc.)
- Also consider participation in the future maintenance of the facilities.

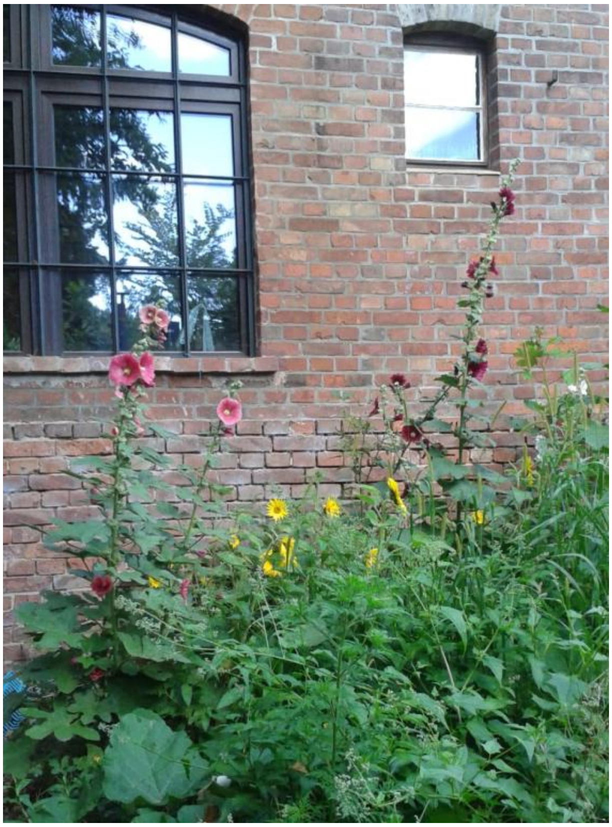

3. Create planning draft - define goals!

- What type of measure is preferred? Please read the following description of measures in Chapter 3.
- First design draft in cooperation with landscape planners and architects. What stumbling blocks are in the way: what is feasible, where are the limits?
- Consultation with local residents. Does the design meet the expectations? Do you have any concerns or questions?

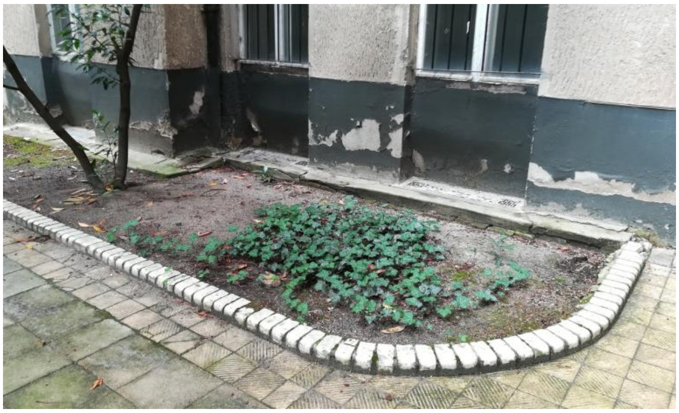

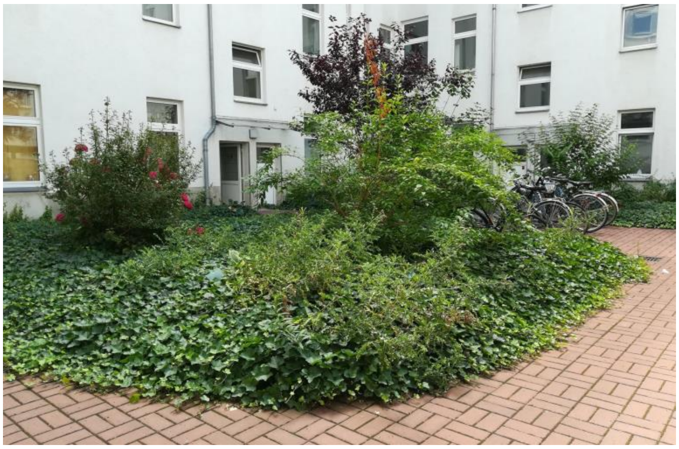

4. Create costs and scedule - less is often more!

- What financial resources are available? Find out about possible funding measures, e.g., green roofs as an environmentally friendly and energy-efficient building project.
- In what period of time is the redesign planned from the start of planning to the completion of the measure?
- How high is the maintenance effort for the individual measures?
- Are there opportunities for self-government by local residents or are trained nursing staff required? Responsibilities and schedules must be drawn up for this.
- How high are the total costs including maintenance?

Contact person:

A discussion with competent experts is worthwhile for the successful implementation of the measures. The following contact points are available to you for further information:

• Garden and landscape planners and landscape architects
• Environmental and nature conservation associations
• Conservation Agency/ Lower monument protection authority

2 Green transformation

The most common urban structure types in major German cities are explained below.
The decision matrix at the end of the guide goes into the individual structures in connection with redesign measures and names the respective suitability, gives implementation advice and points out possible stumbling blocks.
It serves as a specific orientation and decision-making aid.

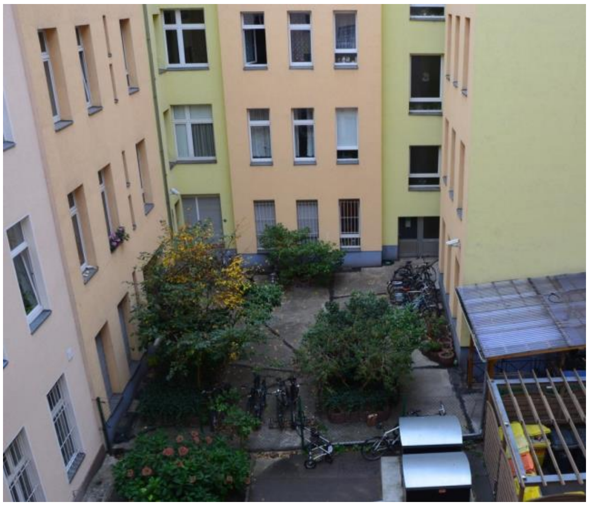

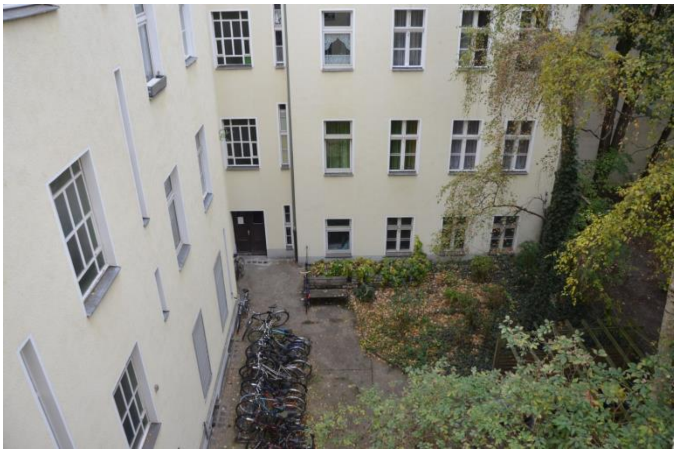

A: Block development

The building structure from the years 1870-1918 has an (almost) closed, mostly 4-6 storey development. The plots are built on with a front building, side wings and rear building. This results in the typical backyards, mostly with concrete courtyard areas, and there are also isolated flower beds.

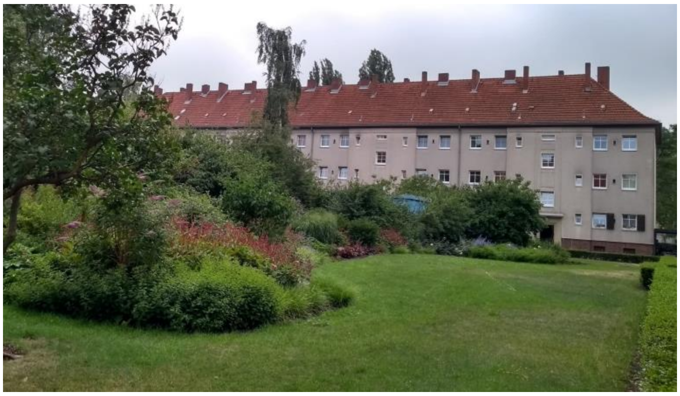

B: block perimeter development

Since the 1920s, perimeter block development with its 3-4 storey, (almost) closed structures has been the main focus. This building structure has a square perimeter development with mostly green inner courtyards, which usually contain a lawn and a few trees. In addition to the green inner courtyards, there are also paved or concreted areas.

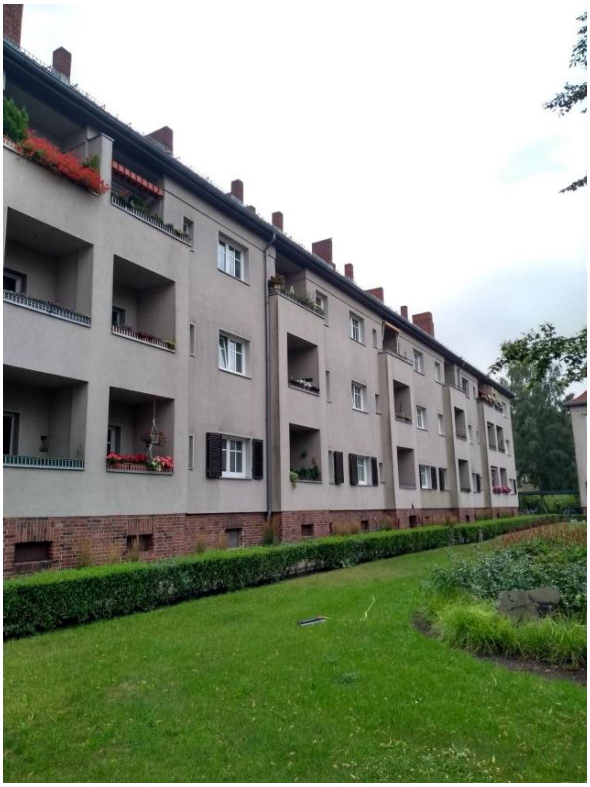

2 Green transformation

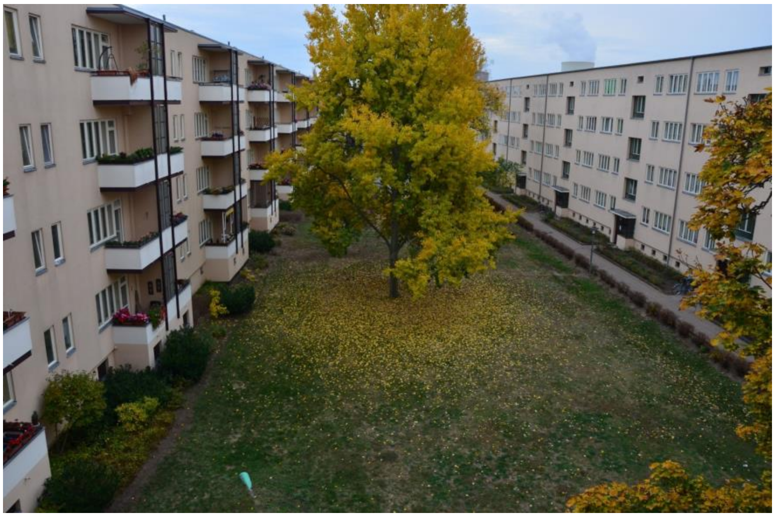

C: Row development

The mostly 4-storey rows of houses often run in a row, resulting in larger, elongated open spaces that are connected to each other. These are usually in the form of lawns with selective bushes and trees.

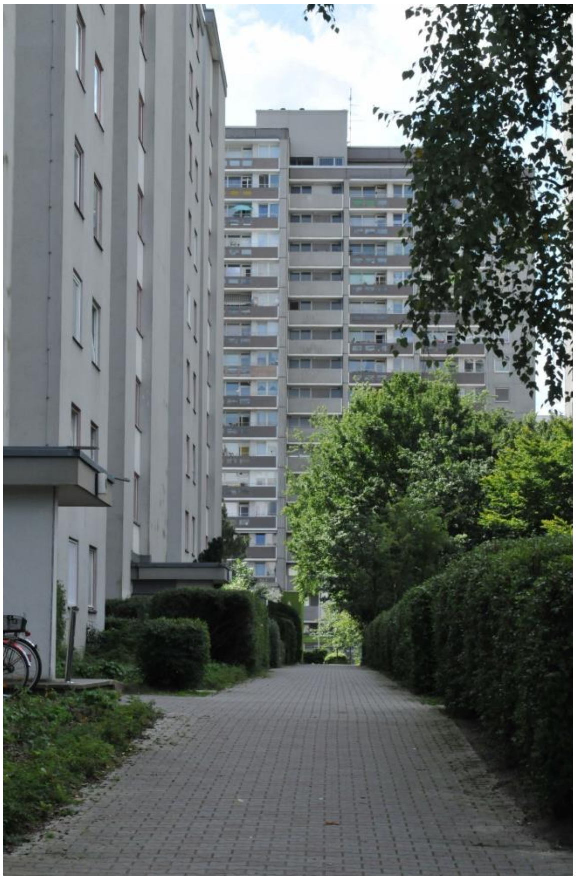

D: High-rise buildings / prefabricated buildings

The rows or point houses with different block or row constructions usually have more than 6 floors. The large part of the undeveloped area is mostly concreted as a parking lot or with ornamental gardens, through which access paths run.

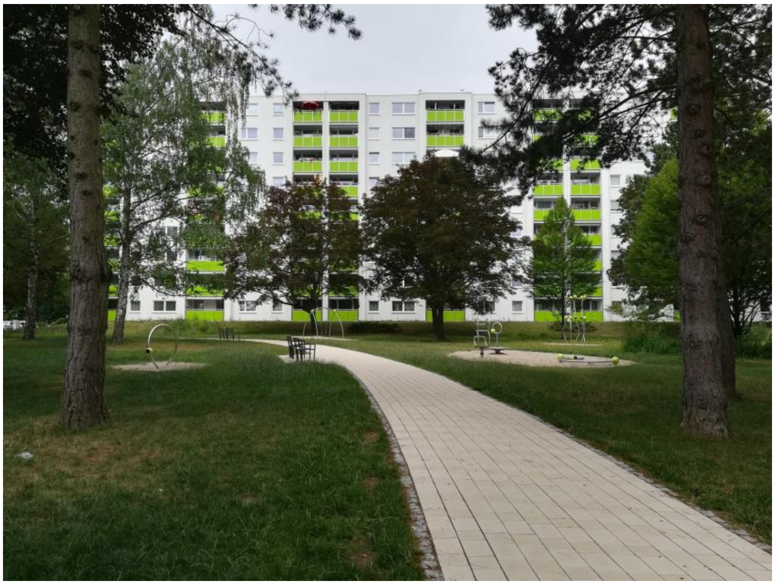

The classification of the structure types is based on the template of the *HealthyLiving* Project based on the Berlin Environmental Atlas.

See also:
www.stadtentwicklung.berlin.de/umwelt/environmental atlas.de/da607_03.htm2

2 Green transformation

Red thread of the green transformation

From the measures listed in Chapter 3, some principles can be derived that are applicable to all design ideas.

Unseal urban soil

Urban soils show an extreme degree of sealing. The soil as a storage and buffer, both for water and to compensate for temperature fluctuations, can only fulfill its natural function if it is not sealed. The top priority of the green transformation is therefore the unsealing of the soil.

The open ground provides the basis for all design measures, it creates space for plants and animals to settle and to significantly improve the local microclimate.

Plant choice

When designing near-natural areas, a decision must first be made as to whether this is a temporary or permanent measure.
When choosing plants, our native nature should be given preference. It contains a large selection of plants that are most adapted to our climatic conditions in their life cycle. In addition, the livelihood of many native insects and animal species is strengthened and ecological communities are made possible.

Ecological and structural diversity

Diversity of plants and structures create an equally large variety of habitats, which are essential for a stable ecosystem. Overgrown areas offer retreats for small animals such as hedgehogs, lizards and birds.
Nevertheless, a certain care of the areas is essential to avoid pest colonization.

Building materials

Structural elements for the construction or delimitation of new constructions consist primarily of wood, natural stone or gravel. Asphalt or concrete paving, while easy to clean, seals the floor and is impervious to water.

A biological engineering construction method can be used for areas that require protection against erosion or settlement. This is an advantage on sloping surfaces and sandy soils. Dead materials such as stone, rubble or dead wood are combined with living material, with the root power of the plants protecting the soil from erosion. Among other things, willow rods or sticks that reproduce vegetatively are suitable.

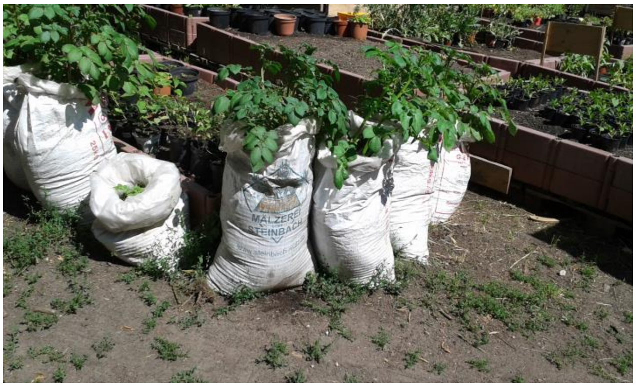

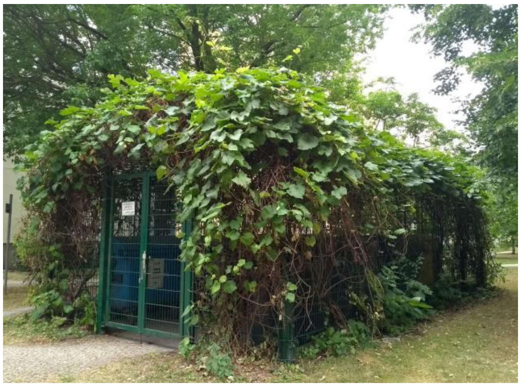

3 Green roofs

Especially in densely populated areas, where little free space is available, roofs become attractive as unused space. Roofs can be harnessed using a variety of approaches. Regardless of whether it is a simple green roof or an intensive roof garden - even simple elements can create green oases from small areas such as shed or garage roofs.
Depending on the system, green roofs create an improved bioclimate by having both thermal insulation and cooling properties.
As a result, the buildings are exposed to lower temperature fluctuations, which is particularly difficult for city dwellers. In particular, the resulting energy savings for residents is worth mentioning, which depends on the thickness of the substrate.

Where a complete roof cannot be planted with greenery, raised beds can be created and planted according to the wishes of the residents. In addition, benches, tables and individual play elements create a private recreation area above the roofs of the big city.

Extensive and intensive green roofs:

When it comes to green roof measures, a distinction is made between extensive and intensive green roofs. Both differ mainly in their substrate density and the associated maintenance effort, which is particularly important in the statics of the building. A green roof is built up in several layers, which in ascending order includes the protective layer, drainage layer, filter layer, substrate layer and vegetation layer. In single-layer constructions, the drain layer is integrated with its function in the substrate layer.
Depending on the substrate density, herbs, grasses, mosses and sedum species in particular can be planted on extensive green roofs, while perennials and shrubs can also be planted on intensive green roofs. Roofs with a pitch of 0-15 degrees are most suitable for green roofs, since they do not require additional technical safeguards, which makes their implementation easier.

When calculating the superimposed load, the entire structure with vegetation in a water-saturated state is taken into account. The roof structure is also considered: is it a single-layer thermal roof or a double-layer cold roof?

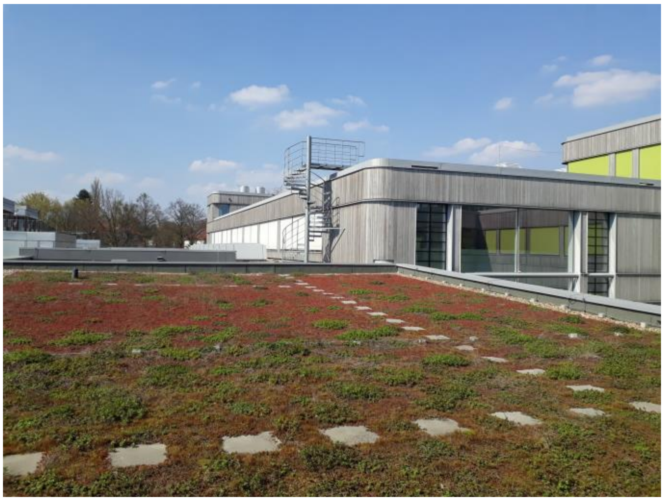

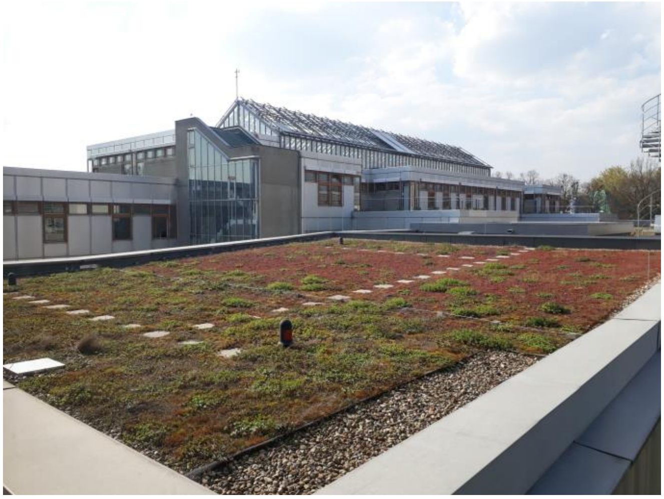

3 Green roofs

Alternative roof use:

Depending on the initial situation, individual raised beds can alternatively become your own roof garden. They have the advantage that they mean less load for the roofs and offer the inhabitants more possibilities to implement their own design ideas. Both the joy of ornamental plants and your own vegetable garden can be lived out here on an equal footing. Benches and tables invite you to linger and allow a piece of nature in the immediate vicinity of your own home.

What is to be considered?

- Is our roof waterproofing root-resistant?
- Are our statics suitable for the ballast of the green roof (extensive / intensive)?
- Is fall protection planned? (terrain construction)
- Is our project in conflict with monument protection? (Information from the lower monument authority)

...this and much more can be found in the FLL Green Roof Guidelines

Extensive roof greening	Intensive green roof
**Plant communities:** mosses, grasses, herbs and sedum species<br>**Cost and care:** low, watering only at sowing<br>**Layer thickness:** 6 - 15 cm<br>**Weight:** 60 - 180 km/m²	**Plant communities:** as seen left including shrubs, perennials and shallow-rooted trees<br>**Cost and care:** high, regular watering<br>**Layer thickness:** 15 - 100 cm<br>**Weight:** 180 - 1500 kg/m²
A high biological value is ensured above all by a varied terrain.<br>Even with extensive greening, smaller hills can be created, wildflower meadows and grasses planted, which primarily benefit insects and contribute to biodiversity.<br><br>When choosing plants, pay attention to plant communities of mosses, herbs and grasses that are adapted to the dry and sunny habitat.	There are more design options for intensive green roofs, even if this means more maintenance.<br>Shrubs and bushes on roofs are justified, especially if there is no ground-level open space available. Examples of trees and shrubs can be:<br><br>Dogwood, cotoneaster, potato rose, summer lilac, currant, boxwood and ornamental quince as well as lavender, sage and bluebells.

3 Facade greening

Just like green roofs, green facades also have properties that can improve the urban climate.
Their function consists less in active use like a roof garden, which invites you to garden and linger, but more in improving the urban climate.
Temperature differences of up to 3 degrees can be measured on a facade with ivy plants due to evaporation and shading. In addition, green facades can have a positive effect on noise pollution on busy streets.
Especially in street canyons, traffic noise penetrates the interior of the building. Depending on leaf thickness, leaf size and leaf position, the soft vegetation layer can create a buffering effect. In addition to the sound-insulating effect, the reduced pollution caused by fine dust in buildings exposed to the street is also worth mentioning.

With facade greening, a distinction is made between ground-based and wall-based systems.

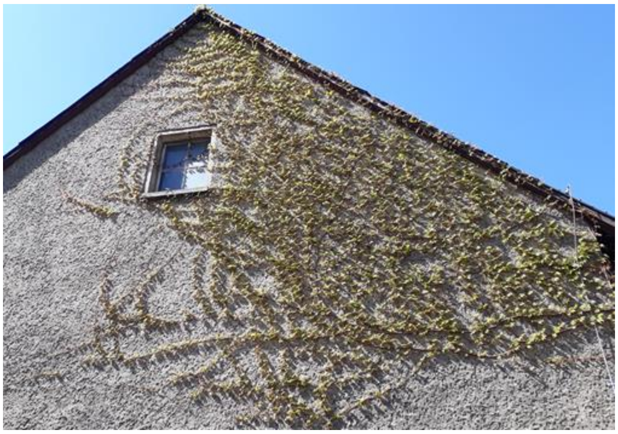

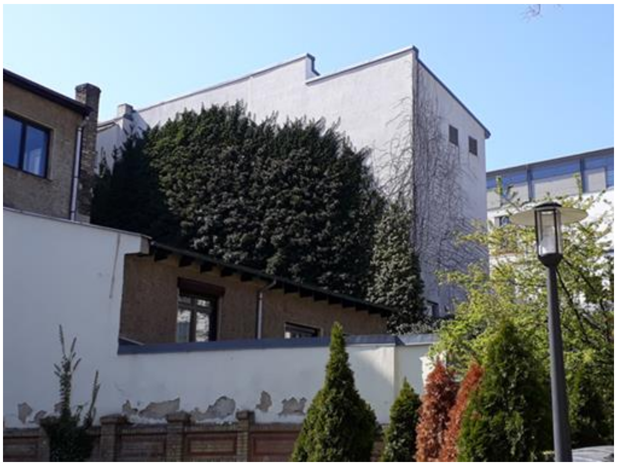

Advantages and disadvantages:

Ground-based systems have the advantage of requiring less construction and, depending on the type of planting, require less maintenance.

Furthermore, they require little space and can therefore also be used well in small open spaces. Ground-based systems are irrigated exclusively via the root system in the ground.

Depending on the choice of plants, they can climb the house wall independently or with the help of scaffolding.
With all the advantages that climbing plants offer, the disadvantages cannot be neglected. Depending on the growth characteristics, insufficient care can lead to damage to the building. Self-climbers (e.g. ivy) climb the facade with the help of adhesive organs. Rough and porous facades can be damaged by the adhesive elements when they are removed.

In the case of old buildings, it is advisable not to use self-climbing plants or to switch to scaffold climbing plants. Strongly adhering ivy is not a problem on smooth or painted facades, although the Virginia creeper penetrates less deeply into the plaster.
Plants that flee the light can penetrate crevices and cracks with their shoot tips and cause damage there. Entwined parts of the building can be subjected to loads and deform, even break, particularly if the thickness increases significantly. It is therefore advisable to prevent plants from getting under the shingles and to refrain from these species on porous facades.

Wall-bound systems require higher technical demands because the plants are not rooted on the ground but in wall constructions.

3 Facade greening

These structures can be roughly divided into

- Planters anchored horizontally to the wall
- modular systems, where planting modules are attached to the facade like tiles and the plants root vertically
- flat constructions, whereby the vertical planting does not take place in modules but forms a vertical substrate surface.

Since the plants are rooted on the facade and not on the ground, continuous, artificial irrigation must be guaranteed. Due to the construction and maintenance costs for wall-mounted systems, the costs of facade greening are significantly higher.

In contrast to ground-based systems, the selection of plants is all the more diverse, and the insulating / cooling effect is increased through evaporation.

In new buildings, wall-mounted systems can be easily integrated into the planning, or parts of large facade areas can be greened with them.

What do I have to consider when choosing plants?

Across the system, the choice of plants is based on various climatic factors, including the sun exposure of the facade (the orientation of the sky, hours of sunshine and shadows from neighboring buildings must be taken into account!), the structural quality of the facade and the wind flow.
In general, the same applies to facade greening as to green roofs: before any measure is taken, the building fabric, the associated choice of plants and conflicts with monument protection must be considered.

Which plant species are suitable for what?

self climbers:

Common ivy (*Hedera helix*)
Tricuspid vine (*Parthenocissus tricuspidata*)
Climbing Hydrangea (*Hydrangea petiolaris*)

trellis climbers:

Knotweed (*Fallopia baldschuanica*)
Clematis (*Clematis vitalba*)
Wisteria (*Wisteria sinensis*)
True hop (*Humulus lupulus*)

The following types are worth mentioning for wall-mounted systems:

Balkan cranesbill (*Geranium macrorrhizum*)
Purple bells (*Heuchera micrantha*)
Sedges (*Carex spec.*)
Bergenia (*Bergenia cordifolia*)

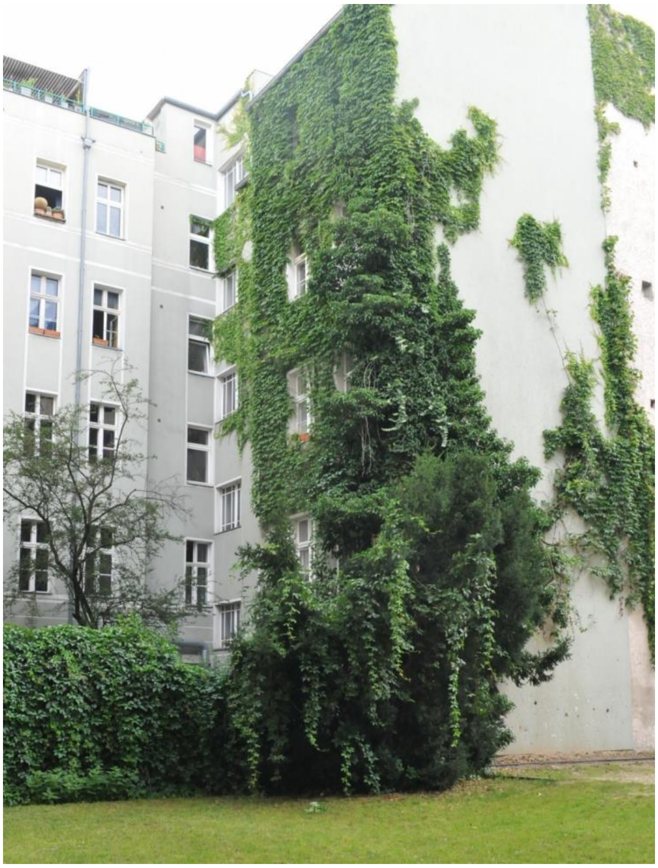

3 Meadows and wild shrubs

Almost everyone who owns their own garden has it - the lawn.
From perfectionism to moss-covered open spaces: when it comes to the idea of the perfect lawn, opinions differ.
The fact is, however, that a bright green lawn with dense vegetation and without "weeds" can only be obtained with a lot of effort. If you want a perfect green carpet, you have to invest a lot: a lot of water, fertilizer, care and time. This no longer has much to do with near-natural design and biodiversity.

However, it is not necessary to do without a usable open space. Open spaces covered with herbaceous vegetation do not have a significant cooling effect like trees and shrubs but serve as rainwater infiltration areas. In addition, meadows have a primarily cultural ecosystem service.

At least since the acting Federal Environment Minister Saskia Schulze presented her action plan for insect protection, the ecological relevance of pollinators in Germany has been well known. After a few years ago the death of bees caused a changed perspective on our agricultural landscape, with the current decline in the diversity of our insects, the call for action has also reached mainstream society.

Wildflower mixtures and manuals for the correct installation of a so-called "bee pasture" are more in demand than ever.

The wildflower meadow also has a special place in this guide.
In contrast to the other measures, the focus here - in addition to improving the living climate - is in particular on preserving biodiversity at the place of residence.

Conversely, this does not mean that flowering mixed plantings have a negligible effect on human well-being.

However, a meadow is not just a meadow. The colorful wildflower meadows in particular have a shelf life of up to three years, depending on the seed. The flowering area changes over time and will appear differently in the following years than before. Good preparation of the soil (specifically: harrows) and up to two mowings per year are necessary for the success of the measure. For the correct installation and care of your wildflower meadow, use information from landscape conservation sources.

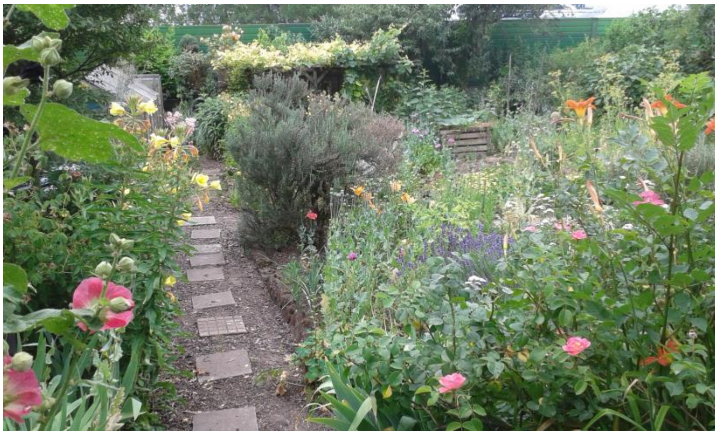

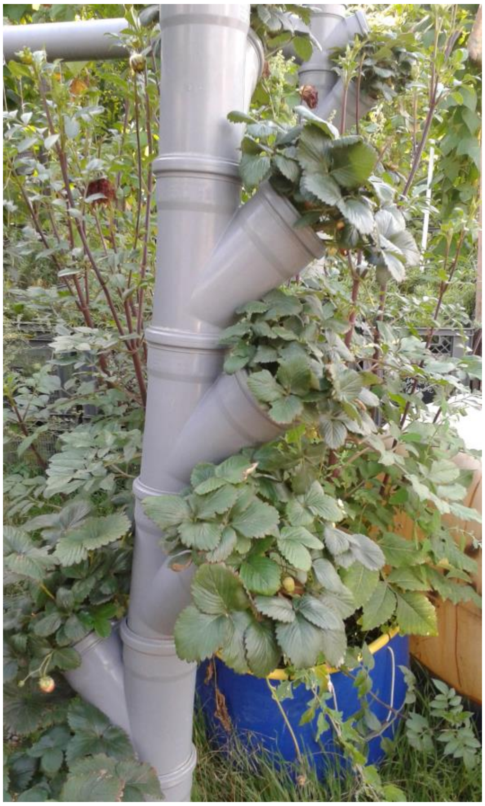

3 Meadows and wild shrubs

While perennial wildflower meadows focus on bee pasture and their aesthetics, perennial meadows also benefit non-pollinating insects and can make significant contributions to biodiversity. With the right system and care, such meadows can exist almost indefinitely. Their disadvantage, however, lies in the more complex system. Thus, the soil composition may have to be changed in order to form a basis for the desired vegetation. Added to this is the fact that existing vegetation must be completely removed from the ground in order to avoid repopulation.

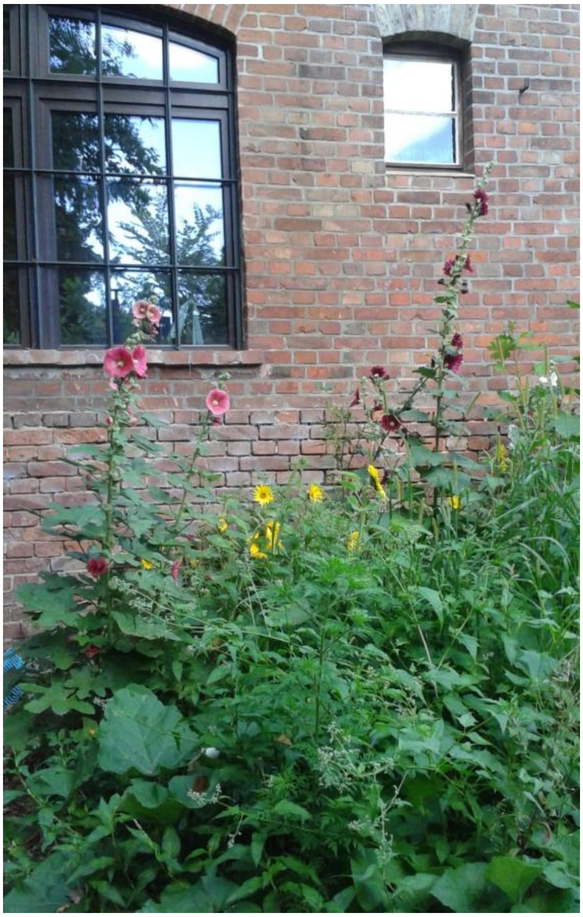

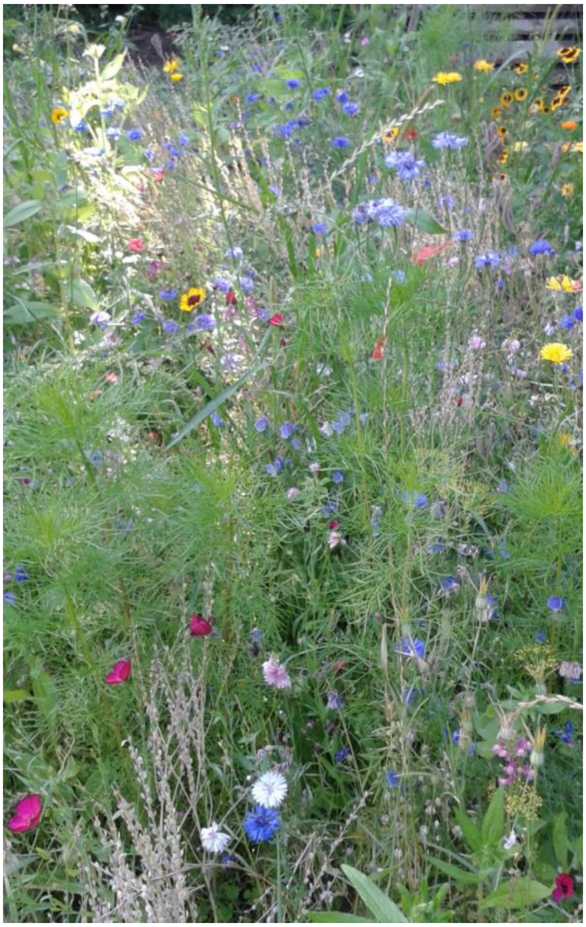

In order not to disturb the ecological balance of the meadow, it is also not suitable as a play area and should only be walked on rarely or not at all. Existing allergies to grass can also lead to health problems in the residents - this must be taken into account when planning.

3 Meadows and wild shrubs

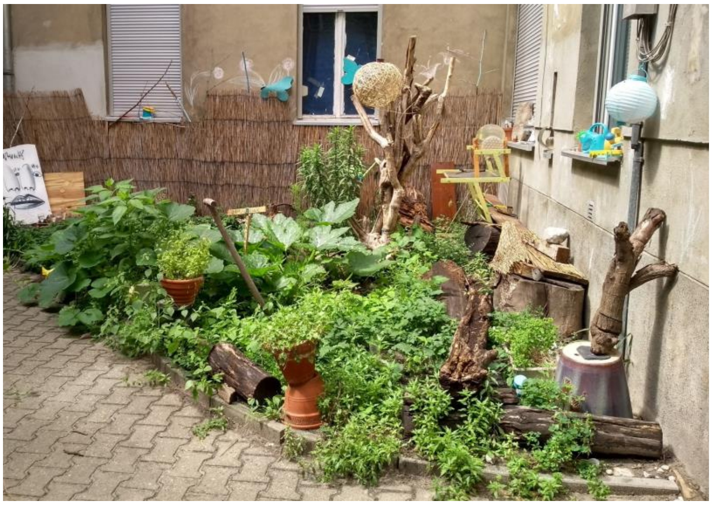

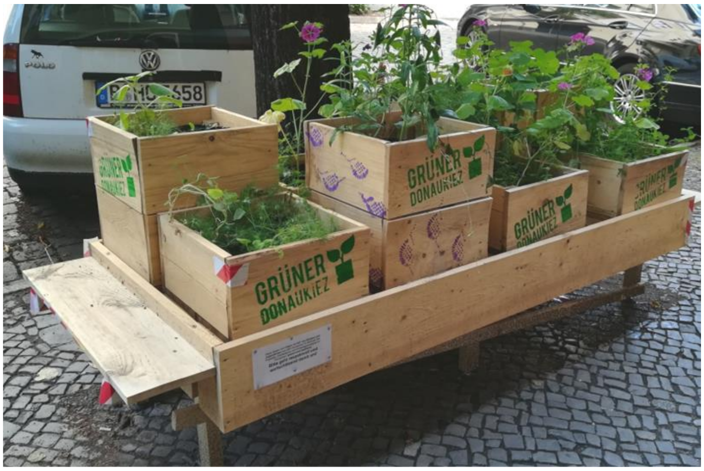

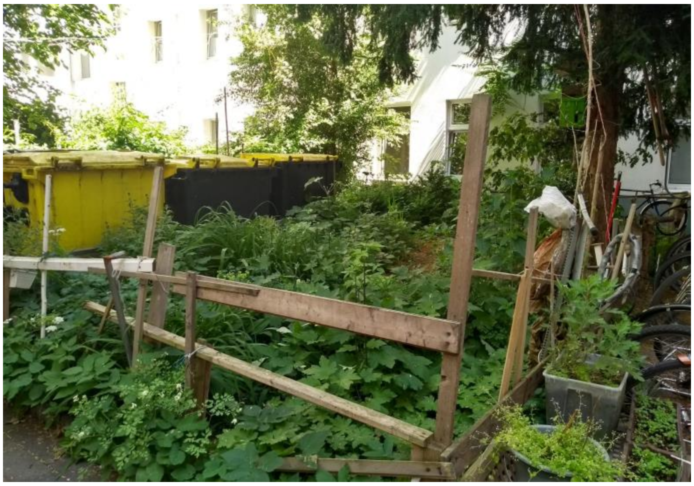

Wild perennials as an alternative when there is a lack of space

In the case of small green areas in the inner-city center, such systems are only recommended to a limited extent. If there is not enough space for a perennial wildflower meadow, alternatives such as flowering hedges (see: Shrubs, hedges and bushes) and borders, as well as perennials are conceivable. The advantages of this are obvious:

Wild perennials such as yarrow, meadow sage or lavender can easily be planted in raised beds or tubs and placed in sun-exposed areas of a courtyard. This alternative is extremely space-saving and an open floor is not a requirement. In sealed courtyards in particular, they help to upgrade the existing space. When selecting the species, note the root depth and maximum growth height, as well as the duration of direct sunlight.

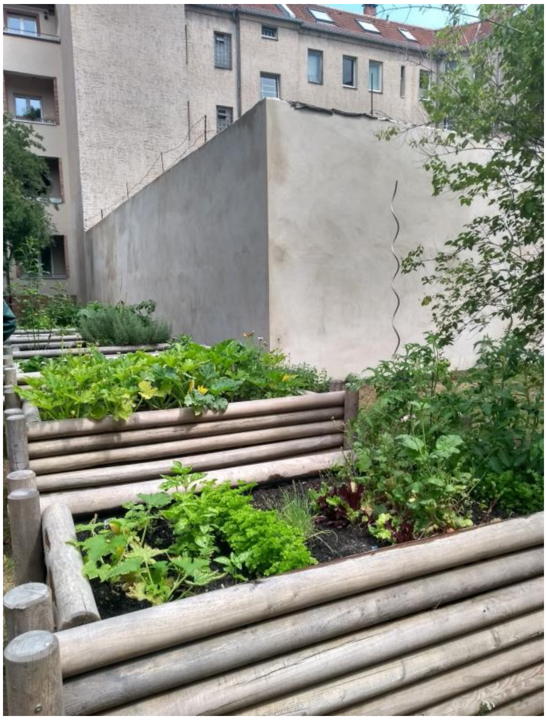

3 Actively design lawns

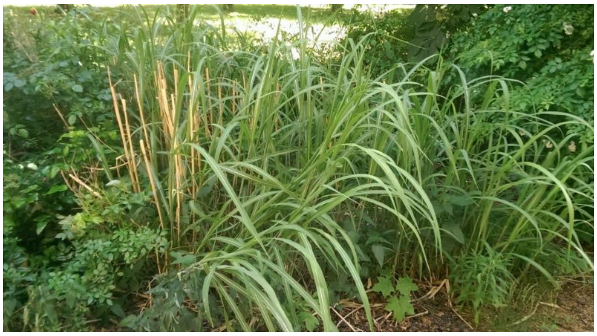

The great importance of (urban) nature for children and their development can be found in detail in the TEEB Natural Capital Germany - "Green in the City"; as well as in the BfN scripts 203 "Children and nature in the city".

Playgrounds in natural surroundings are of particular importance as children become more and more “domesticated”.
It is not the equipment and complexity of technical game elements that is important, but the sole existence of scope.

Areas that are close to nature in particular offer space for imaginative design of the environment, where children's urge to discover can be lived out.
A combination of technical play elements such as swings, slides and climbing frames and natural areas can be implemented well in almost all open spaces, depending on the design.

Wild play areas can be designed in a variety of ways to appeal to all ages.

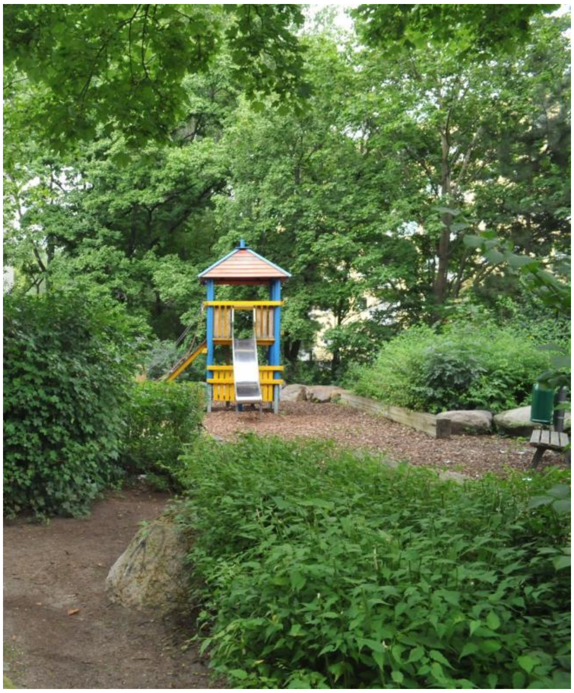

Implementation ideas:

- Swings, slides and climbing frames require a suitable surface on sealed surfaces to prevent injuries.
- Sandboxes can be found in any outdoor facility and are easy to integrate anywhere, regardless of the surface.
- Uneven lawns and meadows not only offer an advantage for the existing flora and fauna - they are also used by children to run, jump and romp.
- The same applies to plant elements such as trees and bushes - they offer a variety of opportunities to stimulate children's creativity, be it for hiding, climbing or stretching out hammocks or suspension ropes.
- Willow structures are of particular importance here as natural play elements in the living environment; they can be laid out independently by local residents.
- In the case of larger open spaces, it is advisable to create a multi-generational park (also: movement course). In addition to the classic game elements, this is supplemented by fitness equipment that encourages visitors of all ages to exercise and relax. The aim is to convey fun and joy in physical activity across generations.

3 Bodies of water

Ponds are undoubtedly a highlight in every garden. Well laid out, they are a hotspot of biodiversity and not only delight city animals.
As a constant source of water, they become a magnet for birds, small mammals and insects, as well as residents who watch the hustle and bustle in and around the pond. At the same time, the pond with its vegetation contributes to improving the bioclimate and acts as a CO2 sink.

A prerequisite for creating a pond is the unsealed soil. For this reason, a near-natural pond is only suitable for urban structure types that have sufficient open space with unsealed soil, preferably fallow land or lawns without deep-rooted trees and shrubs. A pond should only be created with the professional help of an expert who is also familiar with the geological conditions of the site (keyword: groundwater level, pipes, etc.).

When implementing, pay attention to the ecological functionality. In order to create a stable ecosystem, the correct zoning in shallow and deep-water zones and the planting of various aquatic plants is important. The location of the body of water also has an influence on the nutrient input, with shading/sun exposure and the input of leaves playing a major role. The care measures therefore include the professional disposal of waste and organic material, as well as the care of the aquatic plants.

A pond is therefore certainly one of the most complex measures for redesigning the living environment, but it also quickly becomes the undisputed social and ecological focal point of any garden area. If parts of the pond are framed with perennials and shrubs, retreats and corridors are created for small animals. Seating delights residents and tempts them to linger, pause and observe.

Make sure there are no break-off points where children can slide straight into deeper sections of the pond. Well-designed zoning with shallow water zones and shore areas made inaccessible by shrubs can make ponds safer for children.

Furthermore, coarse-meshed iron gratings can be placed on parts of the pond that do not create a barrier for vegetation but will arrest a fall.

Find out whether animal noises (e.g. frogs) are perceived as disturbing by the majority of residents.

If your residential area does not have the demanding requirements of a pond system, a simple water source in the form of larger bird baths can develop a similar attraction for residents and animals.
They can be vital for birds, insects and small mammals, especially in inner-city areas, where the degree of sealing is high and freely accessible bodies of water are rare.

Make sure the bird bath is in a visible place where the animals will feel safe and will accept it. Bird baths that are not directly on the ground but on an elevation make them less attractive for "unwanted visitors" such as rats and mice - but also hedgehogs.

Even a simple waterer needs maintenance to protect birds from disease. They must be cleaned regularly, and the water renewed. The use of chemicals is unnecessary and even counterproductive.
A treatment with a coarse brush and hot water is sufficient for cleaning, as well as drying the trough in the sun for a day before refilling it with fresh water.

3 Woody plantations

Deciduous and coniferous trees

Trees are an important part of the living environment greenery. For many people, trees are part of their idea of nature. Recent publications increasingly point to the connection between trees and mental health. It has been proven that spending time in the forest leads to increased well-being and resilience in people.

Urban trees can help reduce mental stress. But not only mental health benefits from urban trees.

Especially for the urban climate, trees can make a significant positive contribution to offsetting thermal stress. Foliage in the growing season reduces the amount of solar radiation reaching sealed ground and buildings and the associated heating of the environment. At the same time, the air is cooled by transpiration.

In particular, the improvement in the bioclimate is perceived as pleasant by local residents.

Homes located near busy roads also benefit from a slight noise reduction, as well as improved air quality through the conversion of CO_2 to oxygen and the associated filtration of air pollutants.
Of all the measures, urban trees are therefore of particular importance, since the associated ecosystem services are very diverse.

Even the occasional planting of trees is a good investment for the living environment.

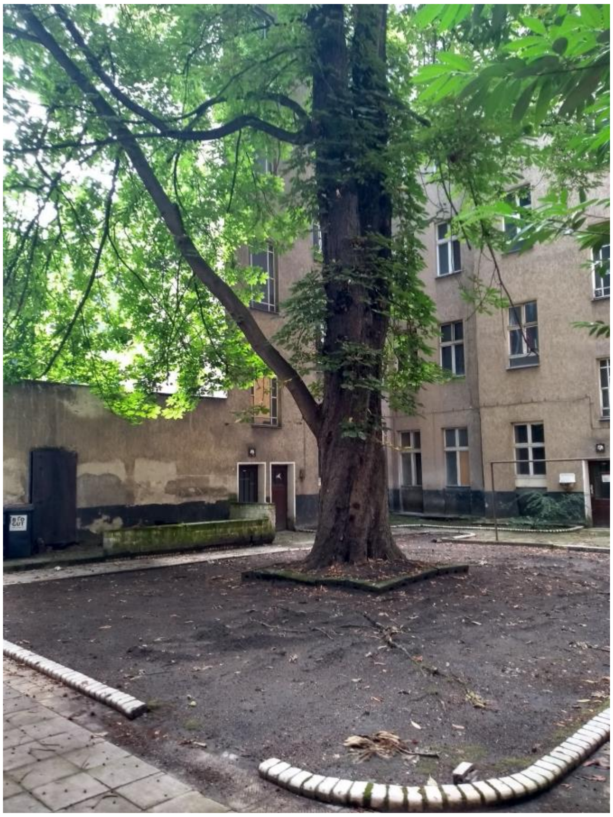

Trees are relatively easy to care for and, depending on the species, reach a great age. They create green islands even in heavily sealed places and as such are also used by the living environment as a stepping stone or island biotope. This can be helped with nesting boxes for bats and birds.

For almost every location, it is therefore advisable to find out about the possibilities of tree planting. Even small inner courtyards are greatly enhanced by trees. Sealed floors can be partially unsealed with a minimum area of 2x2.5m per tree.

3 Woody plantations

When selecting a species, the requirements of the respective species must be taken into account in terms of wet and dry soil. Since the soil in urban areas is often compact, it can make sense to use special devices (e.g. hoses) to ensure sufficient ventilation and irrigation. A root barrier can be installed in the vicinity of pipes. It is important to distinguish between deep-rooted and shallow-rooted trees (various conifers).

Furthermore, when selecting the species, the respective growth behavior must be taken into account - lime trees and oaks reach an impressive height of up to 25 meters, while fruit trees such as cherries and apples rarely grow beyond 15 meters. Especially the planting of fruit trees can be integrated as a horticultural element in urban gardening. Furthermore, fruit trees serve as excellent bee pasture during the flowering period. The removal of leaves in the fall is part of the maintenance measures that exist over the life of the tree.

Although coniferous trees such as pine and spruce do not lose their needles in autumn and appear evergreen, they should be used with caution by small children and are not always recommended in the immediate vicinity of a sandpit or playground, since the essential oils in the needles are not health-promoting, depending on the coniferous tree. However, they are to be considered as a good complement to deciduous trees.

Health disadvantages that can emanate from trees are mostly associated with allergies to hazel and birch. If possible, it is advisable to switch to other trees.

A special note applies to the planting of gingko trees as an aesthetic element: due to its undemanding nature, gingko has established itself as a city tree, but the seeds of the female plants contain butyric acid, which can lead to a significant odor nuisance in autumn. If there is a desire for gingko, male plants should therefore be used instead.

In addition to planting large and small trees, the additional planting of shrubs is recommended as structuring and space-saving elements.

The exact guidelines for successful tree planting can be found in the information provided by the FLL (Research Society for Landscape Development and Landscaping).

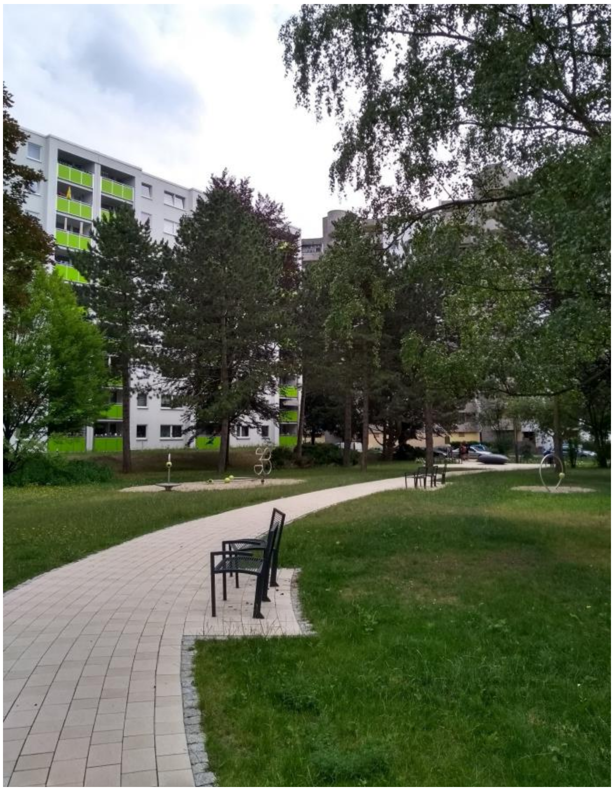

3 Of shrubs and hedges

Shrubs are generally understood to be plants without a continuous main trunk, whose shoots (in contrast to perennials) are woody. Subshrubs have a woody base, with the unwoody branches dying off after each vegetation period and sprout again the following year. While trees have one main trunk, shrubs have many trunks branching near the ground.

If there is only little space, smaller shrubs - similar to perennials - can be planted in tubs and beautify an inner courtyard or peripheral areas.

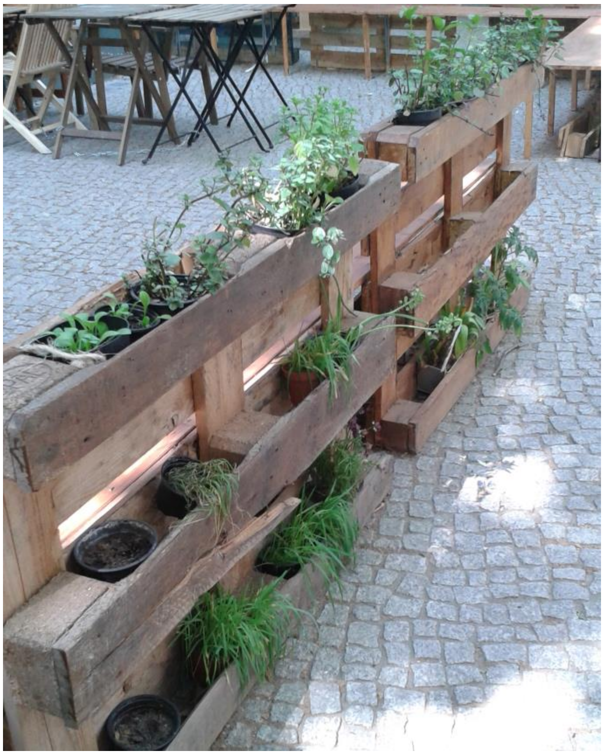

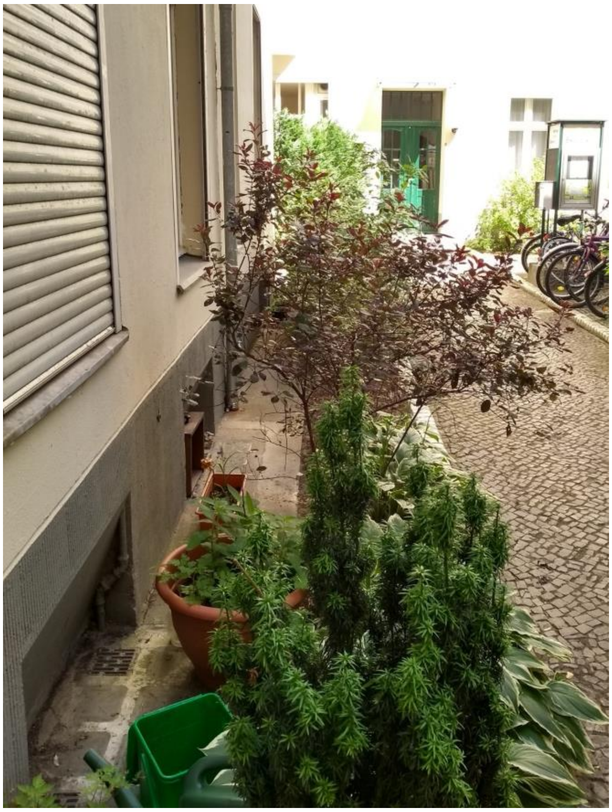

As with woody plants, the health advantage of shrubs lies in their function as primary producers. They help improve city air and create a more comfortable microclimate.

In contrast to trees, the shading rate is low, which means that even in small spaces and close to buildings, plants can be planted without the foliage casting too much shadow on the adjacent apartments during the growing season.

Shrubs can thus be planted selectively in a confined space as a substitute for larger trees. At the same time, they provide a retreat for smaller animals.

3 Of shrubs and hedges

Shrubs are most commonly used as structuring elements in open spaces. They serve as privacy and demarcation. A playground can be demarcated with it as well as the in-house garbage cans. Evergreen plants are recommended.

In the case of more spacious urban structure types such as row and prefabricated buildings, property boundaries and large areas are framed. Evergreen trees and hornbeams (strictly speaking not a shrub but are cut and trimmed as such) are recommended here.

As an alternative to concrete walls and wire mesh fences, living hedges with a distinct border, mantle and core zone can be created if space is available, which offer shelter for birds and mammals as a linear biotope.

In addition to living hedges, there are also structures such as deadwood hedges (so-called Benje hedges) consisting of cut wood and dry-stone walls. The latter are particularly important for insects. They can also be designed as an integral part of seating elements, provided their stability is checked regularly. Both variants impress with their low maintenance requirements and long durability.

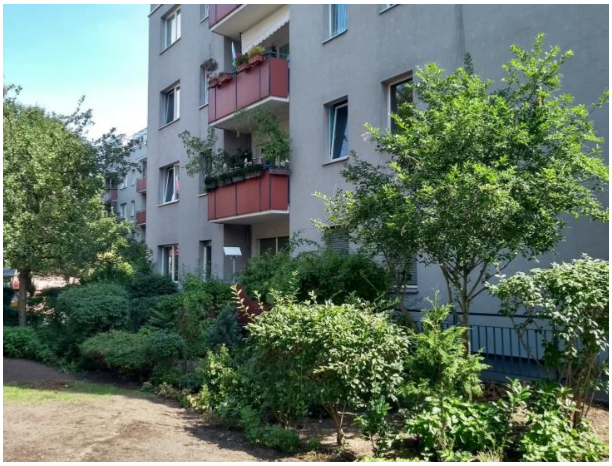

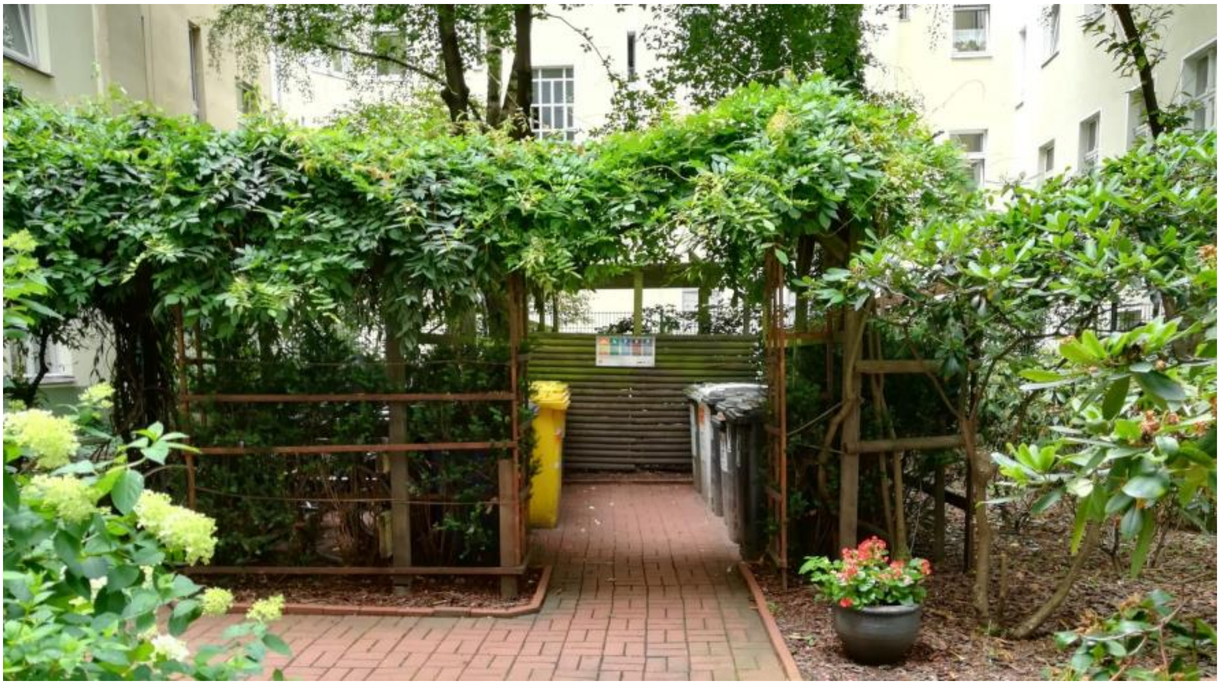

3 Ways, parking lots and peripheral areas

In addition to the measures mentioned above, there are other ways to make the outdoor area more ecological.
Especially where there is not enough space to be active on a large scale, small changes can enhance the living environment and thus lead to improved well-being of the residents.
In particular, the unsealing of the soil is in the foreground in this collection of ideas.

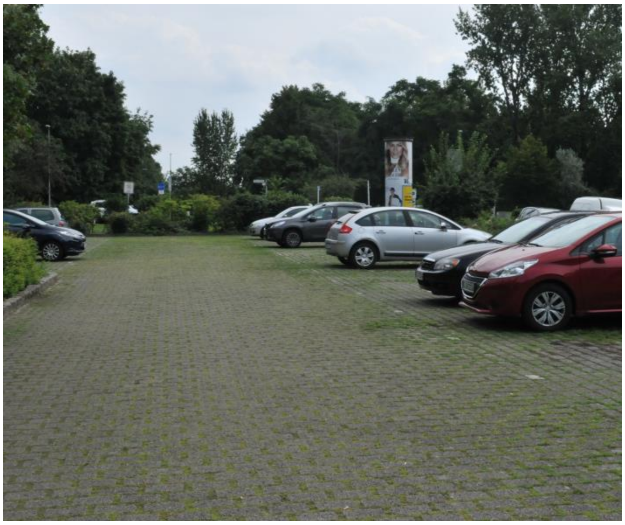

fire brigade access roads

They are mandatory on company premises: According to fire protection law, fire brigade access roads must be accessible and drivable for rescue services with a total weight of up to 16 tons.
However, it is not a reason for an asphalt surface.
Here, a nutrient-poor gravel lawn is suitable as an alternative subsoil. Site-appropriate wildflowers and herbs under 50 cm offer insects a livelihood and ensure a more aesthetic overall picture.

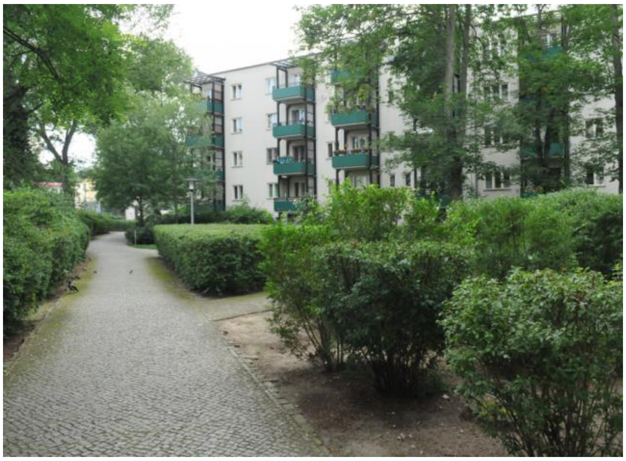

ways

Instead of asphalt paths, split joints, natural stone or gravel are recommended as a water-bound surface, which allow water to drain away.
When designing, in addition to the natural approach, accessibility and traffic safety must also be taken into account.

parking spaces

Spacious parking spaces are available for residents, particularly in the case of residential buildings whose construction dates back to the second half of the last decade.
With large-area sealing of this type, the focus is on water permeability. The selection of the underground is dependent on the frequency with which the parking areas are accessed.

Heavily used parking areas are made more permeable to water by concrete paving with grass or gravel joints and guarantee a resilient subsoil. If there is little use, gravel lawns can be used; it has a higher ecological value.

Trees provide shade between individual parking spaces, which reduces the heating of the parking areas and cars in summer. Elms, oaks and hornbeams are recommended. Linden blossoms lead to sticky panes and should therefore be avoided. Bordered by hedges, parking spaces can be integrated into the overall green picture.

Hints:
The description of the implementation of the measures merely represents recommendations based on technically relevant literature research. Despite the greatest care, there may be deviations in content from other sources.

The content presented is of an explanatory nature and in individual cases does not replace expert advice from those responsible. Please get information from your planning office before implementation.

Status: April 2019

Processing and implementation:
Sonja Mohr-Stockinger

HealthyLiving Projekt:
Dr. Ina Säumel, IRI THESys, Humboldt University Berlin
Dr. Sylvia Butenschön, Institute for Urban and Regional Planning, Technical University of Berlin

Copyright:
Ewelina Skulimowska: pages 1, 5, 7, 8, 9, 10, 13, 14, 17, 18, 20, 22, 23 & 24.
Hannah-Lea Schmid: pages 2, 8, 9 & 21.
Renate Mohr: pages 15, 16, 17 & 22.
Sonja Mohr-Stockinger: pages 11 & 13.

Literature and links:

https://www.stadtentwicklung.berlin.de/umwelt/umweltatlas/
https://www.fll.de/
https://www.futurelandscapes.tu-berlin.de/menue/healthyliving_projekt/
http://www.kuras-projekt.de/
https://www.ufz.de/teebde/
https://www.gebaeudegruen.info/
https://stadtundgruen.de/fokus/deutscher-dachgaertner-verband-ddv.html/
https://www.fbb.de/aktuelles/internationales/details/news/wgin-world-green-infastructure-network/
https://www.db-bauzeitung.de/
https://difu.de/

Exploring the relationship between trees and human stress in the urban environment(2016). J Townsend et al . 42. 146-159.

Nature-Based Solutions to Climate Change Adaptation in Urban Areas. Linkages between Science, Policy and Practice (2017) N Kabisch et al. Springer International Publishing, XI, 342.

Bundesamt für Naturschutz:
Urbanes Grün in der doppelten Innenentwicklung (2016) J Böhm et al. BfN-Skripten 444.
Urbane Grüne Infrastruktur. Grundlage für attraktive und zukunftsfähige Städte. Hinweise für die kommunale Praxis (2017)

<table>
<tr><th></th><th>block development</th><th>reform-oriented perimeter block development</th><th>row settlement</th><th>large housing estates</th></tr>
<tr><td>green roof</td><td colspan="2">Due to the building structure, this is only rarely recommended for block and block perimeter development. Here, private plantings on existing terraces are preferable. If the roof pitch and statics of the building allow for a green roof, also note the monument protection for the selected building.</td><td>If static requirements are met, intensive or extensive roof greening measures are suitable. If there are flat roofs, raised beds can be added.</td><td>Most of the buildings of these structures are best suited for green roof measures. Supplemented by raised beds, they become the new ecological focal point of the residential unit for residents.</td></tr>
<tr><td>facade greening</td><td colspan="2">Due to a high degree of sealing and the small proportion of open spaces close to the ground, facade greening is highly recommended here. Please also note the monument protection of the building here and give preference to climbing aids for ground-based systems. If the structure is porous, it is important to keep an eye on the root and tip growth of the tendril and to cut it back regularly.</td><td>The same applies here: facade greening is a simple option for integrating greenery into the living environment and should always be considered. Self-climbing plants and plants with climbing aids make rows of buildings green. If the building structure allows it, wall-mounted systems can be integrated.</td><td>Due to the better building fabric, more modern buildings are well suited for wall-mounted systems. If this cannot be implemented for financial or planning reasons, ground-based systems are highly recommended.</td></tr>
<tr><td>meadows and wild shrubs</td><td>Paved or concrete inner courtyards often do not allow for lawns. Furthermore, the height of the building and the size of the courtyard determine the incidence of light. Wild perennials can be grown in tubs or raised beds and should be integrated into every yard.</td><td>Depending on the size of the yard and the degree of sealing, meadow sections can be realized. Note the incidence of light and also plant wild perennials. If there is not enough free space, place raised beds or tubs in sun-exposed areas.</td><td>Green spaces between the rows of buildings ensure good incidence of light and are suitable for wild flowers and perennials. Create large or small wildflower beds and complement them with raised beds.</td><td>Larger open spaces enable the creation of a diverse wildflower meadow. Make sure that this is step-protected under certain circumstances by releasing other sub-areas.</td></tr>
<tr><td>open space design</td><td colspan="2">The design largely depends on the size of the yard and the demands of the residents. If there are children, sandboxes and swings can be installed in the yard, regardless of the degree of sealing. Raised beds invite you to garden together, while benches and tables lure residents outside. Even in a small space, create an opportunity for local residents to stay and spend their free time.</td><td>Since row developments often include larger units, the diversity of residents is particularly high. Multi-generation parks are particularly recommended here.</td><td>The population density and the associated diversity can be served by a multifaceted range of design elements. Create social spaces through seating, multi-generation parks and the joint management of e.g., vegetable beds.</td></tr>
<tr><td>bodies of water</td><td>Bodies of water are - depending on the size and sealing of the yard- difficult to integrate and take up a lot of space. Nevertheless, water sources are indispensable for city animals. Provide bird baths.</td><td>In larger courtyards, small bodies of water can be easily integrated. If this is not possible, create other water sources for city animals in the form of bird baths.</td><td>If there is enough space between the rows of buildings, several smaller bodies of water can be integrated in the form of fountains or ponds.</td><td>Bodies of water create an ecological and social hotspot on larger open spaces and should therefore be preferred.</td></tr>
<tr><td>woody</td><td>Trees and shrubs require an area of at least 5m² that is free of sealing, but 6m² is better according to DIN 18916. Note the incidence of light and possible shading from low-lying apartments during the growing season. Soil quality should be known before planting.</td><td>A larger open space means that trees and shrubs can be easily integrated into perimeter block developments. Pay attention to possible shading of the apartments below and a minimum distance to the building.</td><td>Low trees can be planted several times between the rows of houses and can be supplemented by large trees. Fruit trees are pollinator friendly and provide residents with a variety of choices over the long term.</td><td>Both large and small trees can be accommodated on larger systems. Plant a variety of fruit trees that residents of all ages will enjoy, and optionally incorporate maintenance into an urban gardening project.</td></tr>
<tr><td>shrubs and hedges</td><td>With partially sealed floors, shrubs are an alternative to large trees. Structure the yard by lining borders and parking spaces. If there is a lack of space, plant shrubs in larger containers.</td><td>Line parking spaces, flower beds and verges with shrubs to increase the amount of greenery.</td><td>Shrubs and hedges create structure between the rows of buildings and can frame paths and parking spaces. The combination of different species is of ecological advantage here.</td><td>Integrate shrubs and natural hedges as structuring elements. By planting different species, you create diversity.</td></tr>
</table>
