# Supplementary material for: Awakening the sleeping giant of urban green in times of crisis—coverage, co-creation and practical guidelines for optimizing biodiversity-friendly and health-promoting residential greenery
Source: Front Public Health. 2023 Jun 28;11:1175605. doi: 10.3389/fpubh.2023.1175605 (PMC10345840; doi:10.3389/fpubh.2023.1175605)
Supplement: Supplementary file 3 [file Data_Sheet_2.PDF]

Gesundheitsorientierte Umgestaltung des innerstädtischen Wohnumfeldgrüns

Sonja Mohr-Stockinger

Leitfaden für Wohnungsbaugenossenschaften

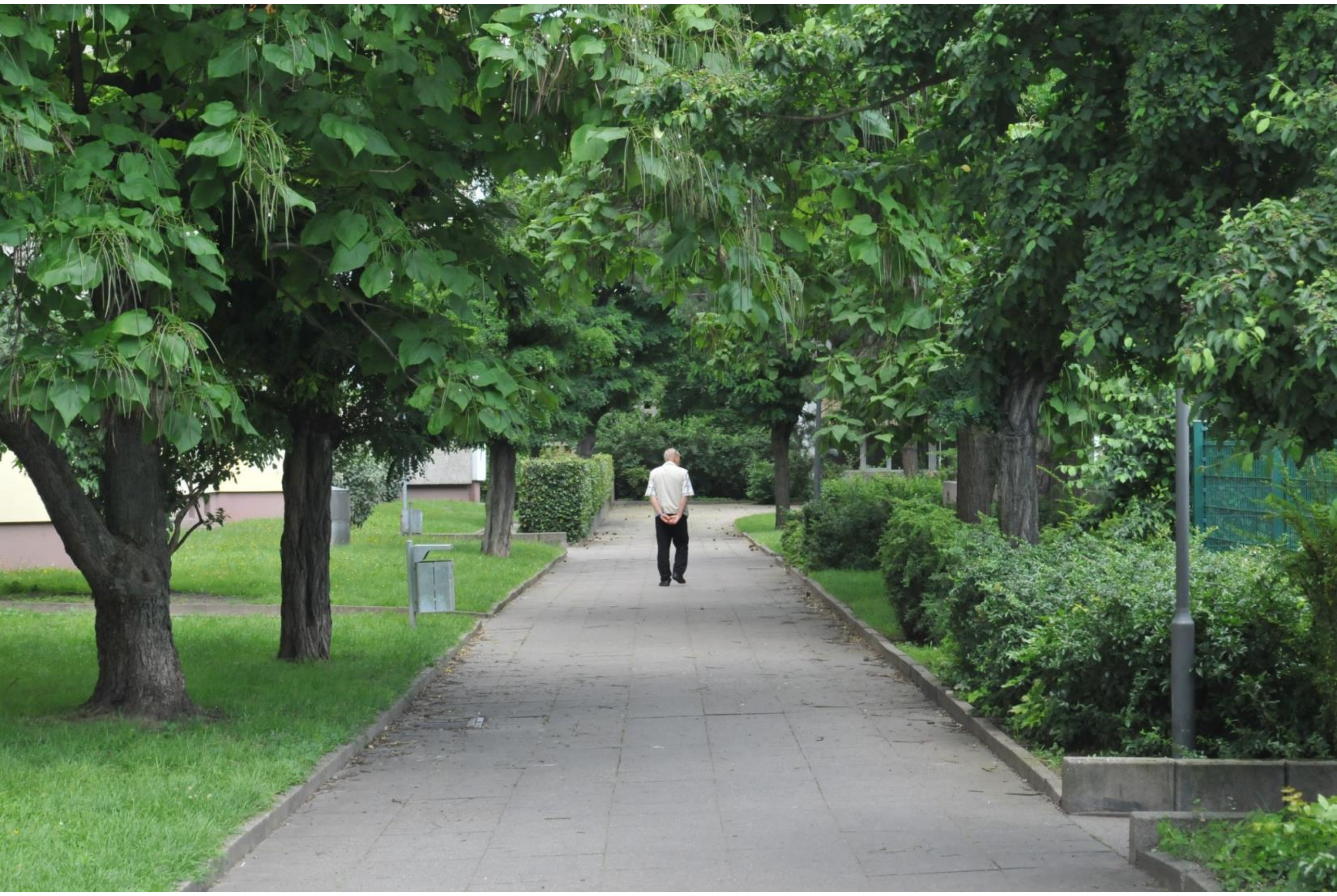

HealthyLiving
Strategie und Planungsinstrument für gesundheitsförderndes Wohnumfeldgrün in der Stadt der Zukunft

IRI THESys

HUMBOLDT-UNIVERSITÄT ZU BERLIN

Technische Universität Berlin

Vorwort

Der Leitfaden “Gesundheitsorientierte Umgestaltung des innerstädtischen Wohnumfeldgrüns“, ist im Rahmen des Projektes *HealthyLiving* an der Technischen Universität Berlin entstanden. Er ist Teil einer Masterarbeit im Studiengang „Stadtökologie“.
HealthyLiving befasst sich mit Strategien der nachhaltigen Stadtplanung, wobei die Ausgleichs- und Entlastungswirkung von Innerstädtischen Grün- und Blaustrukturen auf die Lebensqualität und Gesundheit der Städter betrachtet wird. *HealthyLiving* wird durch die Fritz und Hildegard Berg-Stiftung im Stifterverband für die Deutsche Wissenschaft sowie das Rahmenprogramm FONA (Forschung für nachhaltige Entwicklung) des BMFB gefördert.

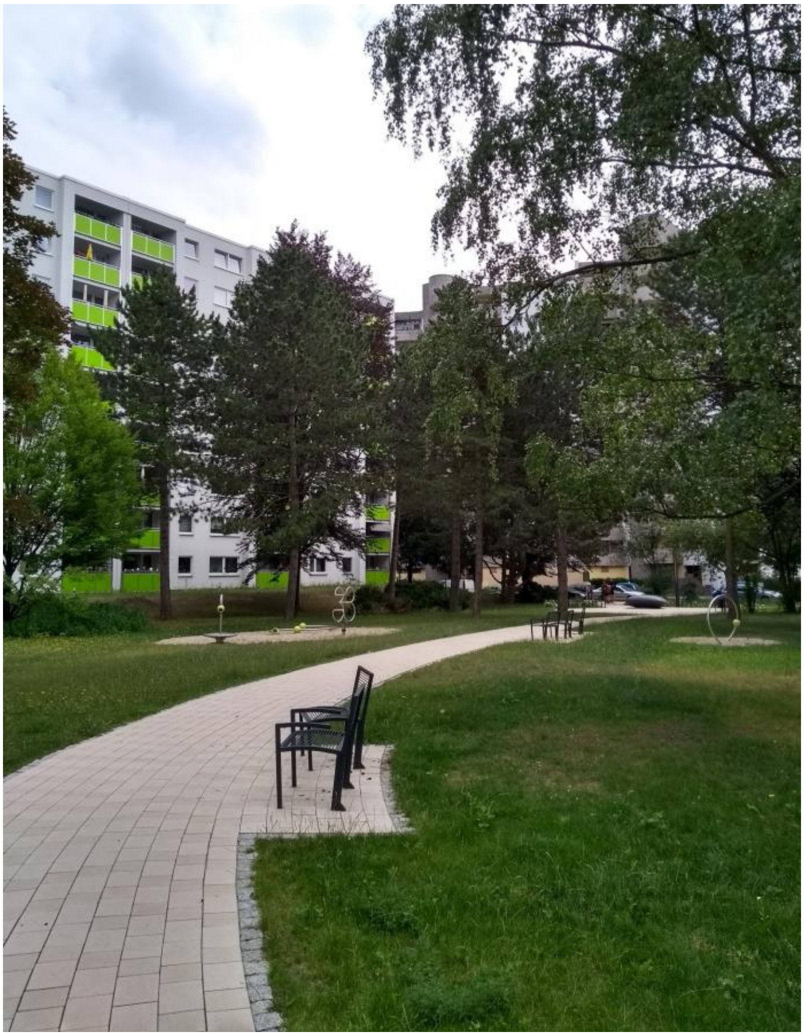

Inhaltsverzeichnis

1 **Stadtnatur**
Die Stadt und ihr Klima
Städtische Belastungen
Stadtgrün und Gesundheit
HealthyLiving Projekt

2 **Grüne Umgestaltung**
Vorgehensweise
Stadtstrukturtypen
Roter Faden der grünen Umgestaltung

3 **Praktische Umsetzung**
Dachbegrünung
Fassadenbegrünung
Wiesen und Wildstauden
Rasenflächen aktiv gestalten
Gewässer
Gehölzpflanzungen
Sträucher und Hecken
Wege, Parkplätze und Randflächen

4 Impressum

5 **Entscheidungsmatrix**

1 Stadtnatur

Die Stadt und ihr Klima

Stadt und Natur sind zwei Begriffe, die in den Köpfen vieler Menschen einen Konflikt beschreiben:
Wo die Stadt entsteht, hat Natur keinen Platz. Dieser vermeintliche Widerspruch ist aus heutiger Sicht überholt und ein Relikt aus jener Zeit, als viele Landstriche und naturnahe Gelände baulichen Strukturen weichen mussten. Der Bedarf an arbeitsnahem Wohnraum stieg in Zeiten der Industrialisierung an, und somit wurden Städte mehr und mehr zum neuen Wohnort der arbeitenden Gesellschaft.
Seit jeher wachsen Städte weltweit kontinuierlich: laut der World Health Organisation WHO leben derzeit mehr als 50% der Weltbevölkerung in Städten- ein Trend, der unaufhaltsam fortschreitet.

Die steigende Bevölkerungsdichte stellt Städte vor ganz neue Herausforderungen: neuer Wohnraum muss geschaffen und gleichzeitig so gestaltet werden, dass die Lebensqualität der Städter nicht beeinträchtigt wird.
Im Gegensatz zum naturnahen Umfeld weist der städtische Lebensraum Eigenschaften auf, die die Gesundheit der Stadtbevölkerung einschränken kann.
Insbesondere in strukturschwachen Gebieten ist die sozial benachteiligte Bevölkerung derzeit noch Mehrfachbelastungen ausgesetzt, was zu einer Ungleichheit im Sinne der Umweltgerechtigkeit geführt hat (siehe: Umweltatlas Berlin).

Wie können zukunftsorientierte Städte entstehen, welche den Fokus auf die Klimaanpassung und Gesundheit seiner Menschen legen? Wie kann eine Stadt umweltgerechter werden und die Lebensqualität für alle Bürger_innen erhöhen?

Im Rahmen dieses Leitfadens wird das Stadtklima und seine Besonderheiten vorgestellt und Maßnahmen erläutert, die gesundheitsorientierte Ökosystemdienstleistungen liefern.

Ziel ist es, einen praxisorientierten Überblick über die komplexe Thematik der Stadtnatur und ihren Einfluss auf das menschliche Wohlbefinden zu geben.

Städtische Belastungen

Städte weisen einen hohen Anteil an baulichen Strukturen auf. Stein, Stahl und Beton kühlen im Winter aus, heizen sich im Sommer hingegen drastisch auf. Somit kommt es mitunter zu großen Temperaturgefällen zwischen dem Stadtkern und dem Umland. Versiegelter Boden behindert zudem die natürliche Wärmeregulation durch fehlende Verdunstung. Insbesondere die mangelnde Abkühlung bei Nacht hat Städte zu sog. Hitzeinseln gemacht. Hitzeinseln sind für den menschlichen Organismus eine große Belastung. In sommerlichen Hitzewellen kommt es vorrangig in Städten zu einer erhöhen Mortalität. Bedenkt man die steigenden Durchschnittstemperaturen aufgrund des Klimawandels wird deutlich, dass zum Schutz der Stadtbevölkerung das Stadtklima dringend angepasst werden muss.

Doch nicht nur bauliche Strukturen haben auf das lokale Klima einen Einfluss. Verkehr und Industrie führen zu einer erhöhten Feinstaubbelastung und einer schlechten Luftqualität. Während feinste Partikel in der Luft sich in pflanzlichen Strukturen absetzen und gefiltert werden, verweilen diese Partikel bei fehlender Vegetation in hoher Konzentration zwischen dichter Bebauung und stark befahrenen Straßen.

Ein weiterer Forschungszweig beschäftigt sich derweil mit den Auswirkungen der künstlichen Beleuchtung. Gerade durch den Gebrauch von LEDs als klimaverträgliche Alternative zu Glühbirnen hat sich das nächtliche Straßenbild erheblich geändert. So ist heute die Lichtverschmutzung „Sky glow“ als wichtiger Faktor menschlicher Gesundheit anerkannt.

1 Stadtnatur

Stadtgrün und Gesundheit

Um eine Stadt zukunftsorientiert umzugestalten lohnt sich ein Blick in die Natur, um ihr gesundheitliches Potential zur Minderung diverser städtischer Einflüsse wie Lärm und Luftqualität, zu erkennen und zu nutzen.

Nicht nur die zuvor erwähnten Belastungen führen zu einer Reduktion des Wohlbefindens, häufig ist es auch die Lebenssituation in der Stadt, die maßgeblich an dem Gesundheitszustand der Städter beteiligt ist.
Die positiven Beiträge, die das urbane Grün bietet, werden im Konzept der Ökosystemleistungen dargestellt.

Ökosystemleistungen sind im Allgemeinen definiert als die indirekten und direkten Beiträge, die die Natur zum menschlichen Wohlergehen bereitstellt. Die Beiträge, die im städtischen Kontext von Bedeutung sind, lassen sich primär in drei Kategorien der regulierenden, kulturellen und versorgenden Leistungen einordnen.
Regulierungsleistungen führen zu einer Belastungsminderung des Stadtklimas, der Luft und der Gewässer.
Versorgungsleistungen beinhalten die Trinkwasserversorgung und die Bereitstellung von Rohstoffen wie Holz und Nahrung aus urbanem Anbau.
Kulturelle Leistungen hingegen beschreiben die Erholungs- und Bildungsbeiträge, die die Gesellschaft aus der Stadtnatur zieht.
Sind ökologischen Funktionen der Stadtnatur einer Gesellschaft von Nutzen, erhalten sie somit das Prädikat der Ökosystemleistung.

In diesem Zusammenhang ist es wichtig zu erwähnen, dass die Nutzung der Ökosystemleistungen je nach Kultur- und Werteverständnis variieren und in verschiedenen Gesellschaften oder Gruppierungen unterschiedlich genutzt werden können.

Im Detail sind die Ökosystemleistungen im „TEEB Naturkapital Deutschland“ nachzulesen.

Für eine gelungene Implementierung von Stadtgrün in vorhandene Strukturen ist es notwendig, die Möglichkeiten und Grenzen zu benennen. Bevor die einzelnen Begrünungsmaßnahmen im Folgenden beschrieben werden, wird die Baustruktur analysiert. Basis liefern die Daten der Berliner Senatsverwaltung, auf denen auch die Kategorisierung des *HealthyLiving* Projekts fußt. Daraus ergeben sich grob 4 Kategorien, in welche sich die Mehrheit der Berliner Mehrfamilienhäuser einordnen lassen.
Um die Entscheidung für eine der beschriebenen Begrünungsmaßnahme zu erleichtern, sind die Maßnahmen und die Baustrukturtypen in einer Matrix grafisch dargestellt, um Potentiale zu erkennen, Alternativen vorzuschlagen und bewerten zu können.

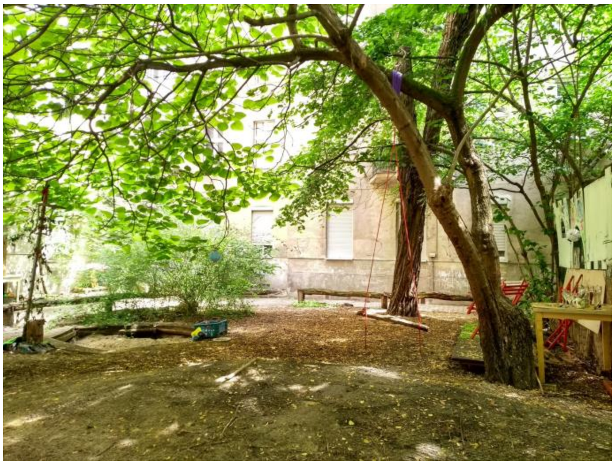

2 Grüne Umgestaltung

Vorgehensweise

Bevor die praktische Umsetzung des Vorhabens beginnt und gepflanzt werden kann, bedarf es vorab an strategischer Planung. Auf diese Weise können finanzieller und zeitlicher Aufwand abgeschätzt und Sackgassen umgangen werden. Gleichzeitig werden in jeder Planungsstufe alle Beteiligten in den Entscheidungsprozess involviert und können eigene Wünsche, Ideen und Bedenken äußern.

1. Bestandsanalyse- Wie ist der Status Quo?

In einem ersten Schritt wird die Ausgangslage analysiert. Dabei sind folgende Fragen zu beantworten:

- Was für ein Stadtstrukturtyp liegt vor, welcher umgestaltet werden soll? (Sehen Sie dazu die Beschreibung der Stadtstrukturtypen)
- Welche Möglichkeiten und Einschränkungen ergeben sich aus den jeweiligen Bauwerken? Welche Gegebenheiten setzen der Umsetzung Grenzen, welche Vorteile ergeben sich für eine grüne Umgestaltung?
- Sind Freiflächen vorhanden, wenn Ja: in welcher Formation- zusammenhängend oder partiell?
- Welche Bodenbeschaffenheit liegt vor, wie hoch ist der Versiegelungsgrad?

2. Anwohner mobilisieren- Partizipation leben!

- Was sind die Wünsche und Bedürfnisse der Anwohner? Hierbei ist eine interkulturelle Kompetenz von Vorteil. Die Maßnahmen müssen auch auf die Altersstruktur der Anwohner abgestimmt werden (Sitzgelegenheiten, Spielplätze etc.).
- Mängel aufdecken, gibt es Bedenken? (Allergene Pflanzungen, giftige Sträucher etc.)
- Möglichkeit zur Partizipation ausschöpfen (Siehe: Urban Gardening, Dachbegrünung, etc)
- Partizipation auch bei der zukünftigen Pflege der Anlagen bedenken

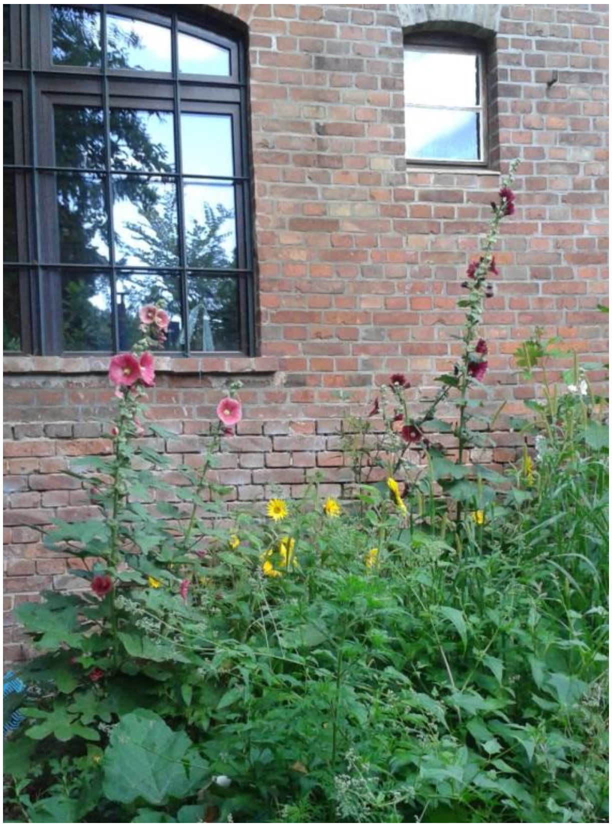

3. Planungsentwurf erstellen- Ziele definieren!

- Welche Art von Maßnahme wird präferiert? Lesen Sie dazu die folgenden Maßnahmenbeschreibungen in Kapitel 3.
- Erster Gestaltungsentwurf in Zusammenarbeit mit Landschaftsplanern und Architekten. Welche Stolpersteine liegen im Weg: was ist machbar, wo sind die Grenzen?
- Rücksprache und Konsultation von Anwohnern. Entspricht der Entwurf den Vorstellungen? Gibt es Bedenken oder Rückfragen?

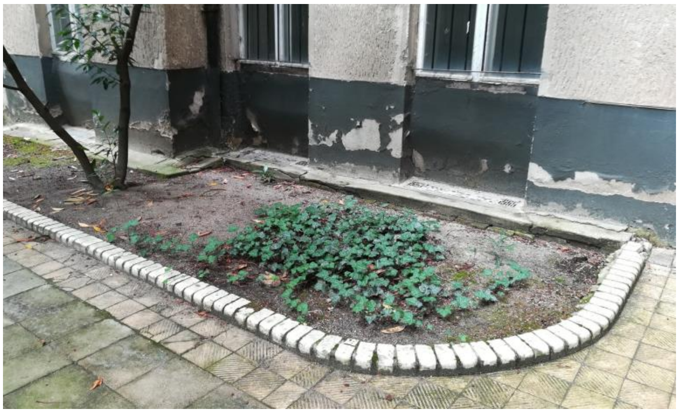

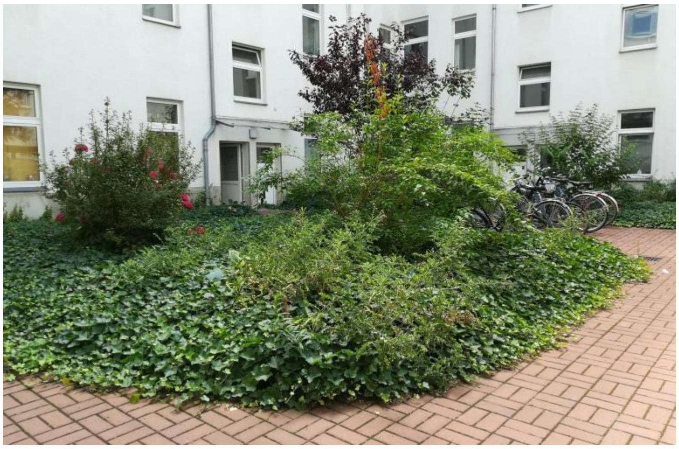

4. Kosten und Zeitplan erstellen- weniger ist häufig mehr!

- Welche finanziellen Mittel stehen zur Verfügung? Informieren Sie sich auch über mögliche Förderungsmaßnahmen- z.B. von Dachbegrünung als umweltgerechtes und energieeffizientes Bauvorhaben.
- In welchem Zeitraum ist die Umgestaltung von Beginn der Planung bis zum Abschluss der Maßnahme vorgesehen?
- Wie hoch ist der Pflegeaufwand der einzelnen Maßnahmen?
- Gibt es Möglichkeiten der Selbstverwaltung durch Anwohner oder sind geschulte Pflegekräfte nötig? Dafür sind Zuständigkeiten und Ablaufpläne zu erstellen.
- Wie hoch sind die Gesamtkosten- inklusive der Instandhaltung?

Ansprechpartner:

Für die erfolgreiche Umsetzung der Maßnahmen lohnt sich ein Gespräch mit fachkundigen Experten. Folgende Anlaufstellen stehen Ihnen zu weiterführenden Informationen zu Verfügung:
- Garten- und Landschaftsplaner sowie Landschaftsarchitekten
- Umwelt- und Naturschutzverbände
- Naturschutzbehörde
- Untere Denkmalschutzbehörde

2 Grüne Umgestaltung

Im Folgenden werden die häufigsten Stadtstrukturtypen in deutschen Großstädten erläutert.
Die Entscheidungsmatrix am Ende des Leitfadens, geht auf die einzelnen Strukturen im Zusammenhang mit Umgestaltungsmaßnahmen ein und benennt die jeweilige Eignung, gibt Umsetzungsratschläge und weist auf mögliche Stolpersteine hin. Sie dient als spezifische Orientierung und Entscheidungshilfe.

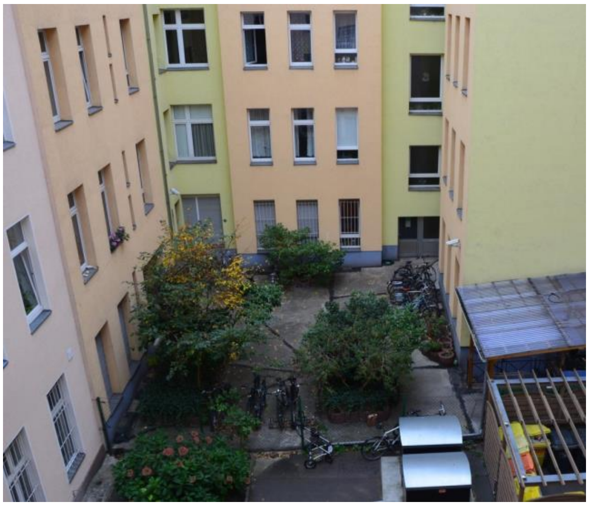

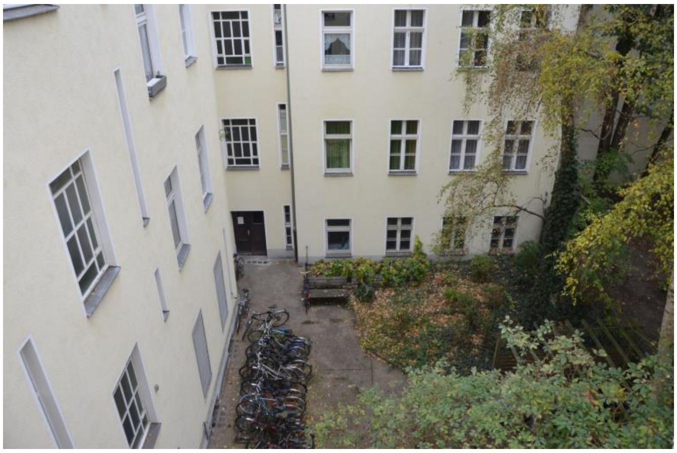

A: Blockbebauung

Die Baustruktur aus den Jahren 1870-1918 weist eine (fast) geschlossene, meist 4-6 geschossige Bebauung auf. Die Grundstücke sind mit Vorderhaus, Seitenflügeln und Hinterhaus bebaut. Dadurch ergeben sich die typischen Hinterhöfe, meist mit betonierten Hofflächen, vereinzelt sind auch abgegrenzte Beete zu finden.

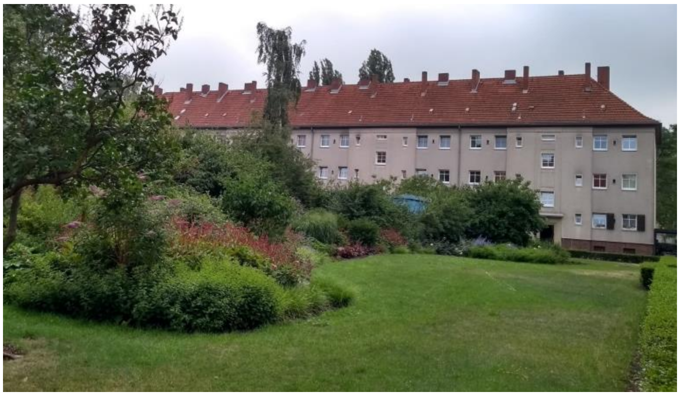

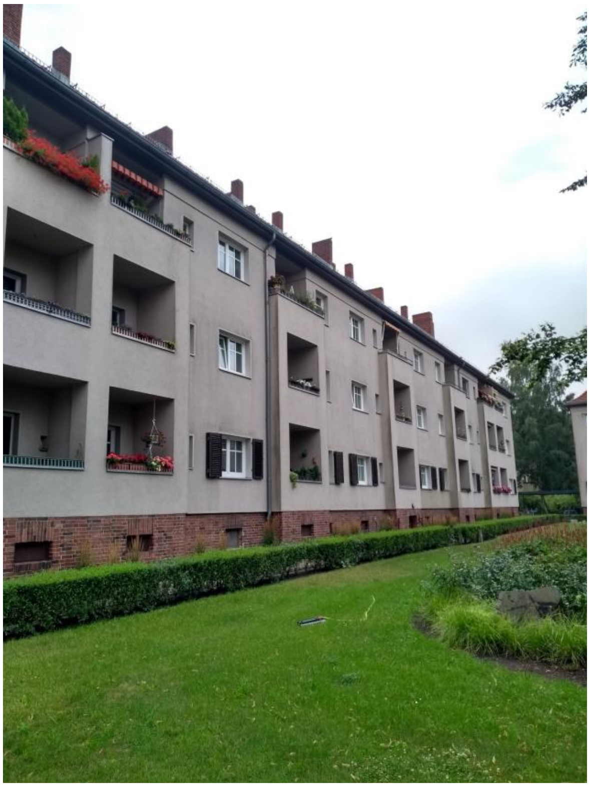

B: Blockrandbebauung

Seit den 1920ern wurde vor allem in Blockrandbebauung mit ihren 3-4 geschossigen, (fast) geschlossenen Strukturen gebaut. Diese Baustruktur weist eine quadratische Randbebauung mit meist grünen Innenhöfen auf, die meist eine Rasenfläche und vereinzelt Bäume beinhalten. Neben den begrünten Innenhöfen sind auch gepflasterte oder betonierte Flächen vorhanden.

2 Grüne Umgestaltung

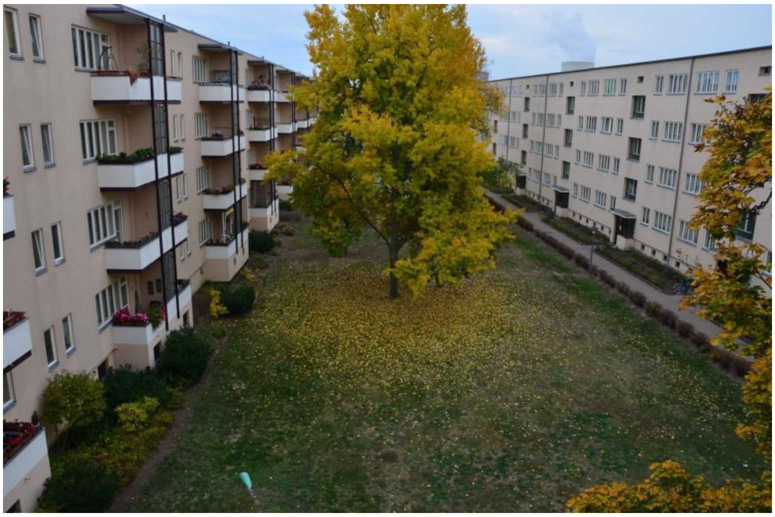

C: Zeilenbebauung

Die meist 4 geschossigen Häuserzeilen verlaufen häufig aufgereiht, wodurch sich größere, lang-gestreckte Freiflächen ergeben, die mit einander verbunden sind. Diese liegen meist als Rasenfläche mit punktuellen Sträuchern und Bäumen vor.

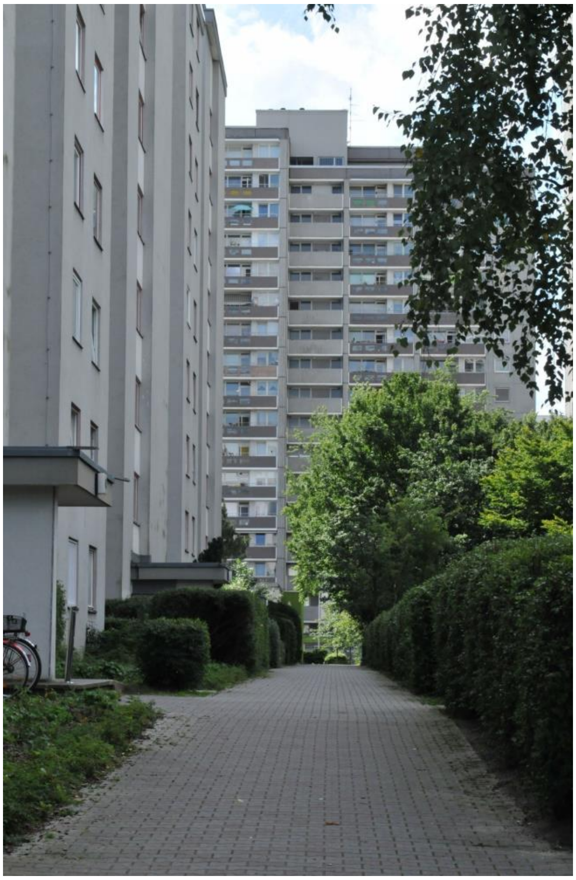

D: Hochhäuser/Plattenbauten

Die Zeilen oder Punkthäuser mit unterschiedlicher Block- oder Zeilenbebauung weisen meist mehr als 6 Geschosse auf. Der große Anteil nichtbebauter Fläche liegt meist betoniert als Parkplatz oder mit angelegten Ziergärten vor, durch die Erschließungswege verlaufen.

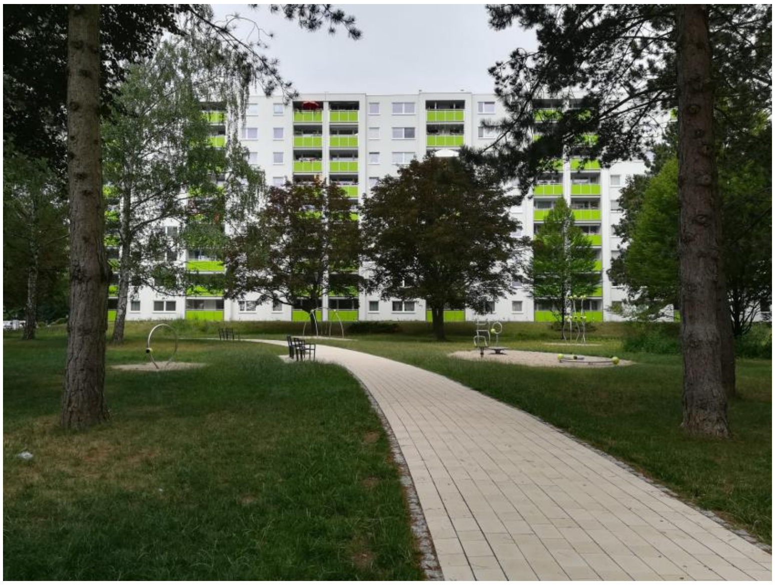

Die Einteilung der Strukturtypen erfolgt nach der Vorlage des HealthyLiving Projekts auf Grundlage des Berliner Umweltatlas.
Siehe auch:
www.stadtentwicklung.berlin.de/umwelt/umweltatlas.de/da607_03.htm

2 Grüne Umgestaltung

Roter Faden der grünen Umgestaltung

Aus den in Kapitel 3 gelisteten Maßnahmen, lassen sich einige Grundsätze ableiten, die auf alle Gestaltungsideen anwendbar sind.

Boden entsiegeln

Städtische Böden weisen einen extremen Versiegelungsgrad auf. Der Boden als Speicher und Puffer, sowohl für Wasser als auch zum Ausgleich von Temperaturschwankungen kann nur im unversiegelten Zustand seine natürliche Funktion erfüllen. Oberstes Gebot der grünen Umgestaltung ist somit die Entsiegelung des Bodens.
Der offene Boden bietet die Grundlage für alle gestalterischen Maßnahmen, er schafft Raum, um Pflanzen und Tiere anzusiedeln und das lokale Mikroklima erheblich zu verbessern.

Pflanzenwahl

Bei der Gestaltung naturnaher Flächen muss zunächst entschieden werden, ob es sich dabei um eine temporäre oder dauerhafte Maßnahme handelt.
Bei der Auswahl der Pflanzen sollte unsere heimische Natur bevorzugt werden. Sie enthält eine große Auswahl an Pflanzen, die in ihrem Lebenszyklus am ehesten unseren klimatischen Verhältnissen angepasst sind. Zudem wird so die Lebensgrundlage vieler heimischer Insekten und Tierarten gestärkt und ökologische Lebensgemeinschaften ermöglicht.

Vielfalt an Pflanzen schafft eine ebenso große Vielfalt an Lebensräumen, die für ein stabiles Ökosystem unverzichtbar sind. Verwilderte Teilbereiche bieten Rückzugsorte für Kleintiere wie Igel, Eidechsen und Vögel.
Nichtsdestotrotz ist eine gewisse Pflege der Bereiche unabdingbar, damit eine Schädlingsansiedlung vermieden wird.

Baustoffe

Strukturgebende Elemente zur Errichtung oder Abgrenzung von neuen Konstruktionen bestehen vorrangig aus Holz, Naturstein oder Schotter. Asphalt oder Betonpflaster sind zwar leicht zu reinigen, versiegeln jedoch den Boden und sind wasserundurchlässig.
Bei Flächen, die einen Schutz vor Erosion oder Setzung benötigen, kann eine Ingenieurbiologische Bauweise eingesetzt werden. Dies ist bei geneigten Flächen und sandigen Böden von Vorteil. Dabei werden tote Materialien wie Stein, Schutt oder Totholz mit lebendem Material kombiniert, wobei die Wurzelkraft der Pflanzen den Boden vor Abtragung schützt. Geeignet sind u.a. Weidenruten oder Steckhölzer, die sich vegetativ vermehren.

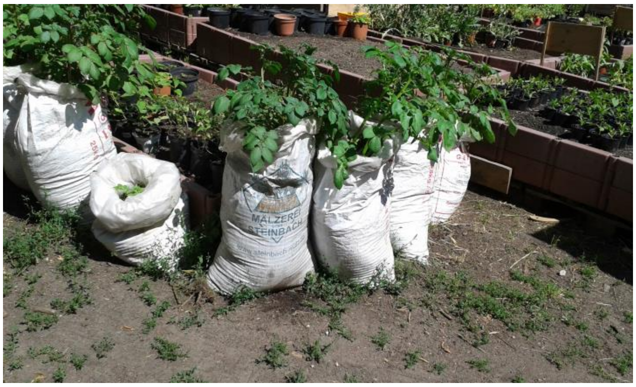

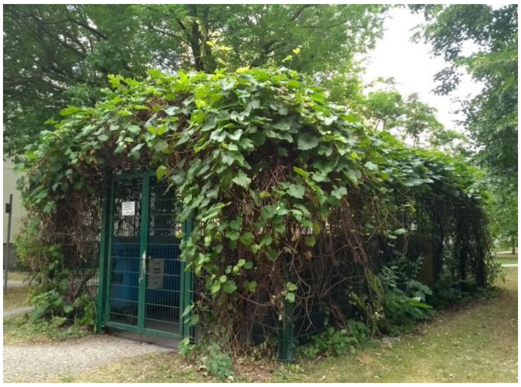

3 Dachbegrünung

Gerade in dichten Siedlungsgebieten, wo nur wenig Freiraum zu Verfügung steht, werden Dächer als ungenutzte Fläche attraktiv. Dächer können mit verschiedenen Ansätzen nutzbar gemacht werden. Egal ob einfache Dachbegrünung oder intensive Dachgärten- schon einfache Elemente können aus kleinen Flächen wie Schuppen- oder Garagendächern grüne Oasen schaffen. Je nach Anlage schaffen Grüne Dächer ein verbessertes Bioklima, indem sie sowohl wärmedämmende, als auch kühlende Eigenschaften besitzen. Dadurch sind die Gebäude geringeren Temperaturschwankungen ausgesetzt, was insbesondere Stadtbewohnern zu schaffen macht. Insbesondere die daraus resultierende Energieersparnis der Bewohner ist erwähnenswert, die sich nach der Substratdicke richtet.
Wo kein komplettes Dach begrünt werden kann, können Hochbeete angelegt und nach den Wünschen der Bewohner bepflanzt werden. Zusätzlich schaffen Bänke, Tische und vereinzelte Spielelemente einen privaten Nah-erholungsraum über den Dächern der Großstadt.

Extensive und Intensive Dachbegrünung:

Bei Dachbegrünungsmaßnahmen unterscheidet man zwischen der Extensiven und der Intensiven Dachbegrünung. Beide unterscheiden sich vor allem in ihrer Substratdichte und dem damit verbundenen Pflegeaufwand, was vor allem bei der Statik des Gebäudes zu beachten ist. Ein Gründach wird in mehreren Schichten aufgebaut, das in aufsteigender Reihenfolge die Schutzlage, Drainschicht, Filterschicht, Substratschicht und Vegetationsschicht beinhaltet. Bei einschichtigen Konstruktionen ist die Drainschicht in der Substratschicht mit ihrer Funktion integriert.
Je nach Substratdichte lassen sich bei der extensiven Dachbegrünung vor allem Kräuter, Gräser, Moose und Sedum-Arten anpflanzen, während bei der intensiven Dachbegrünung auch Stauden und Sträucher gepflanzt werden können. Dächer einer Neigung von 0-15 Grad eignen sich am ehesten für Dachbegrünungen, da sie ohne zusätzliche technische Sicherungen auskommen, was ihre Ausführung erleichtert. Bei der Berechnung der Auflast wird der gesamte Aufbau mit Vegetation im wassergesättigten Zustand berücksichtigt. Auch der Dachaufbau wird betrachtet: handelt es sich um ein einschaliges Wärmedach oder zweischaliges Kaltdach?

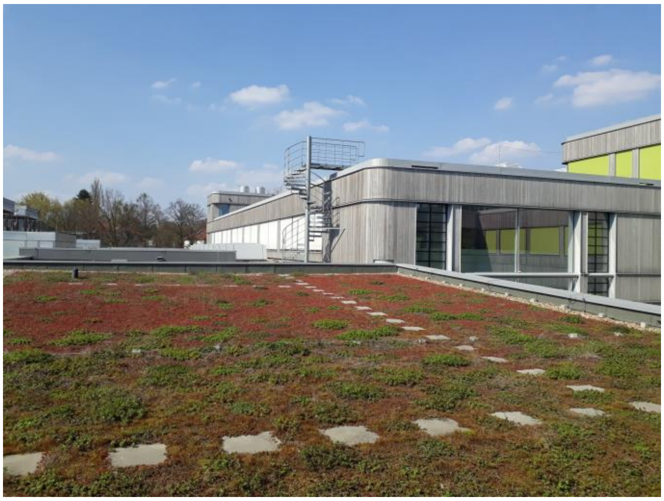

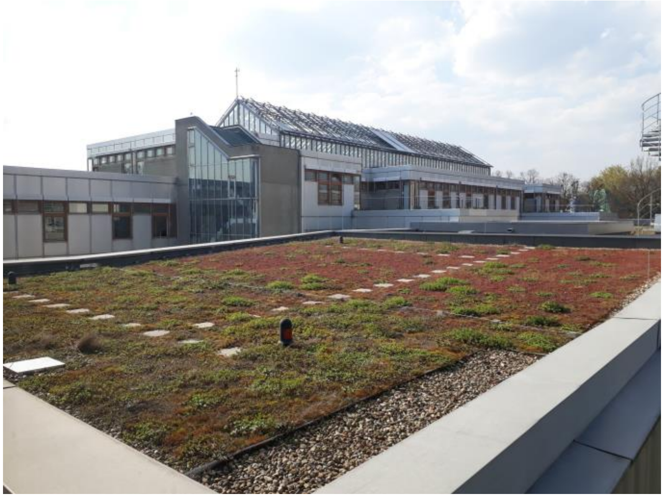

3 Dachbegrünung

Alternative Dachnutzung:

Je nach Ausgangslage können alternativ einzelne Hochbeete zum eigenen Dachgarten werden. Sie besitzen den Vorteil, dass sie weniger Last für die Dächer bedeuten und den Bewohnern mehr Möglichkeiten bieten, ihre eigenen Gestaltungsideen umzusetzen. Sowohl die Freude an Zierpflanzen als auch der eigene Gemüsegarten können hier gleichberechtigt ausgelebt werden. Bänke und Tische laden zum Verweilen ein und erlauben ein Stück Natur in unmittelbarer Nähe zur eigenen Wohnung.

Was ist zu beachten?

- Ist unsere Dachabdichtung wurzelfest?
- Ist unsere Statik geeignet für die Auflast der Dachbegrünung (extensiv/intensiv)?
- Ist eine Absturzsicherung eingeplant? (Geländekonstruktion)
- Steht unser Vorhaben in Konflikt mit dem Denkmalschutz? (Auskunft der unteren Denkmalbehörde)

...dies und vieles Weitere entnehmen Sie der FLL-Dachbegrünungsrichtlinie

Extensive Dachbegrünung	Intensive Dachbegrünung
**Pflanzengesellschaften:** Moose, Gräser, Kräuter und Sedumarten<br>**Kosten und Pflege:** niedrig, Bewässerung nur bei der Ansaat<br>**Schichtdicke:** 6 - 15 cm<br>**Gewicht:** 60 - 180 km/m²	**Pflanzengesellschaften:** auch Sträucher, Stauden und flachwurzelnde Bäume<br>**Kosten und Pflege**: hoch, regelmäßige Bewässerung<br>**Schichtdicke:** 15 - 100 cm<br>**Gewicht:** 180 - 1500 kg/m²
Eine hohe biologische Wertigkeit wird vor allem durch ein abwechslungsreiches Terrain sichergestellt. Auch bei einer extensiven Begrünung können kleinere Hügel angelegt werden, Wildblumenwiesen und Gräser gepflanzt, die vor allem Insekten zu Gute kommen und einen Beitrag zur Artenvielfalt leisten.<br>Bei der Pflanzenauswahl achten Sie auf Pflanzgemeinschaften aus Moosen, Kräutern und Gräsern, die dem trockenen und sonnigen Habitat angepasst sind.	Der intensiven Dachbegrünung stehen mehr Gestaltungsmöglichkeiten offen, auch wenn dies mit einem erhöhten Pflegeaufwand einher geht. Vor allem, wenn keine bodennahen Freiflächen zur Verfügung stehen, finden Sträucher und Büsche auf Dächern ihre Berechtigung. Beispiele für Gehölze und Sträucher können sein: Hartriegel, Zwergmispel, Kartoffelrose, Sommerflieder, Johannisbeere, Buchsbaum und Zierquitte sowie Lavendel, Salbei und Glockenblumen.

3 Fassadenbegrünung

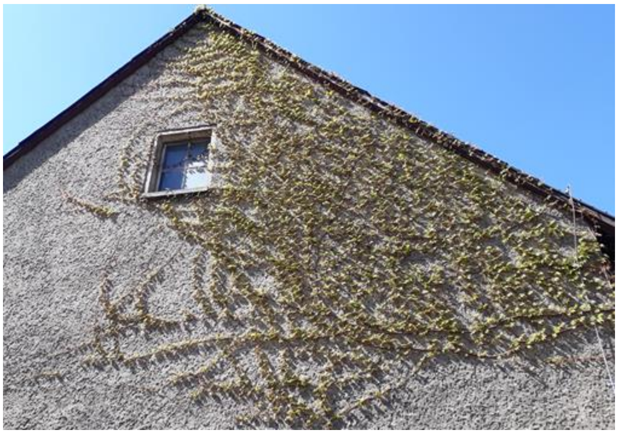

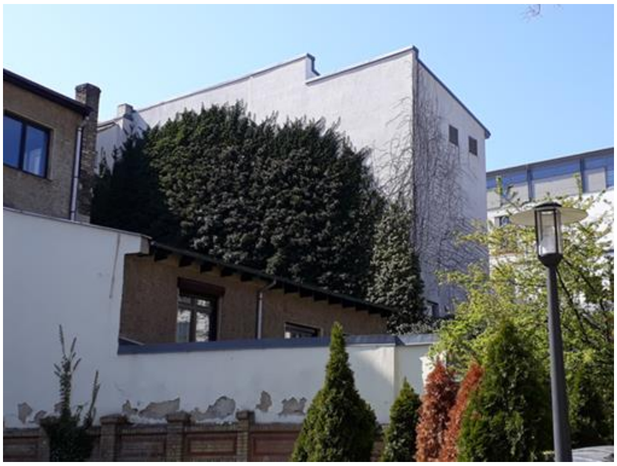

Genau wie Dachbegrünungen besitzen auch Fassadenbegrünungen Eigenschaften, die das Stadtklima verbessern können. Ihre Funktion besteht weniger in der aktiven Nutzung wie ein Dachgarten, der zum eigenen Gärtnern und verweilen einlädt, sondern mehr in der Verbesserung der stadtklimatischen Verhältnisse. Durch Verdunstung und Beschattung können bei einer Fassade mit Efeubepflanzung Temperaturunterschiede bis zu 3 Grad gemessen werden. Darüber hinaus können begrünte Fassaden einen positiven Einfluss auf Lärmbelastungen an stark befahrenen Straßen haben. Grade in Straßenschluchten dringt Verkehrslärm in das Gebäudeinnere. In Abhängigkeit von der Blattdicke, Blattgröße und Blattstellung kann die weiche Vegetationsschicht eine abpuffernde Wirkung erzeugen. Neben der schalldämmenden Wirkung ist auch die verminderte Schadstoffbelastung durch Feinstaub an Straßen-exponierten Gebäuden erwähnenswert.

Bei Fassadenbegrünung wird zwischen bodengebundenen und wandgebunden Systemen unterschieden.

Vor und Nachteile:

Bodengebundene Systeme haben den Vorteil, dass sie weniger Konstruktion benötigen und je nach Pflanzart weniger Instandhaltungsmaß-nahmen benötigen. Des weiteren benötigen sie wenig Platz und sind somit auch bei geringen Freiflächen gut einsetzbar. Die Bewässerung bei Bodengebundenen Systemen erfolgt ausschließlich über das Wurzelgeflecht im Boden. Je nach Pflanzenauswahl können diese eigenständig oder mit Hilfe eines Gerüstes die Hauswand erklimmen.

Bei allen Vorteilen, die Kletterpflanzen bieten, sind die Nachteile nicht zu vernachlässigen. Je nach Wuchseigenschaft kann es bei mangelnder Pflege zu Gebäudeschäden kommen. Selbstklimmer (z.B. Efeu) erklimmen die Fassade mit Hilfe von Haftorganen. Raue und poröse Fassaden können bei der Entfernung durch die Haftorgane beschädigt werden.

Bei alten Gebäuden ist es ratsam, auf selbstklimmende Pflanzen zu verzichten oder auf Gerüstkletterpflanzen auszuweichen. Auf glatten oder gestrichenen Fassaden ist stark haftendes Efeu kein Problem, wobei der Wilde Wein weniger tief in den Putz eindringt. Lichtfliehende Pflanzen können mit ihren Triebspitzen in Spalten und Ritzen dringen und dort Schäden verursachen. Insbesondere bei starkem Dickenwachstum können umschlungene Gebäudeteile belastet werden und verformen gar brechen. Deshalb ist es ratsam, die Pflanzen davon abzuhalten, unter die Dachschindeln zu gelangen, und bei porösen Fassaden von diesen Arten Abstand zu nehmen.

Wandgebundene Systeme erfordern höhere technische Ansprüche, da die Pflanzen nicht am Boden, sondern in Wandkonstruktionen verwurzelt sind.

3 Fassadenbegrünung

Diese Konstruktionen lassen sich grob unterscheiden in

- Pflanzgefäße, die horizontal an der Wand verankert sind
- modulare Systeme, wobei Pflanzmodule wie Kacheln an der Fassade befestigt werden und die Pflanzen vertikal wurzeln
- flächige Konstruktionen, wobei die vertikale Bepflanzung nicht in Modulen erfolgt, sondern eine vertikale Substratfläche bildet.

Da die Pflanzen an der Fassade und nicht dem Boden verwurzelt sind, muss eine kontinuierliche, künstliche Bewässerung gewährleistet werden. Durch den Konstruktions- und Pflegeaufwand bei wandgebundenen Systemen sind die Kosten der Fassadenbegrünung deutlich höher.
Anders als bei bodengebundenen Systemen ist die Auswahl an Pflanzen dafür umso diverser, und der dämmende/kühlende Effekt durch Verdunstung erhöht.
Bei Neubauten lassen sich wandgebundene Systeme gut in die Planung mit integrieren, oder Teile großer Fassadenflächen damit begrünen.

Welche Pflanzenarten eignen sich wofür?

Selbstklimmer:

Gemeiner Efeu (*Hedera helix*)
Dreispitzige Jungfernrebe (*Parthenocissus tricuspidata*)
Kletterhortensie (*Hydrangea petiolaris*)

Gerüstkletterpflanzen:

Knöterich (*Fallopia baldschuanica*)
Gemeine Waldrebe (*Clematis vitalba*)
Blauregen (*Wisteria sinensis*)
Echter Hopfen (*Humulus lupulus*)

Bei Wandgebundenen Systemen sind folgende Arten nennenswert:

Balkan-Storchschnabel (*Geranium macrorrhizum*)
Purpurglöckchen (*Heuchera micrantha*)
Seggen (*Carex spec.*)
Bergenie (*Bergenia cordifolia*)

Was muss ich bei der Pflanzenwahl beachten?

Systemübergreifend richtet sie die Pflanzenwahl nach diversen klimatischen Faktoren, darunter die Sonnenexposition der Fassade (Himmels-ausrichtung, Sonnenstunden und Schatten durch Nachbargebäude sind zu beachten!), der baulichen Fassadenqualität und der Windströmung.
Generell gilt bei Fassadenbegrünung dasselbe wie bei der Dachbegrünung: vor jeder Maßnahme ist eine Auseinandersetzung mit der Bausubstanz, der damit verbundenen Pflanzenwahl und Konflikten mit dem Denkmalschutz zu beachten.

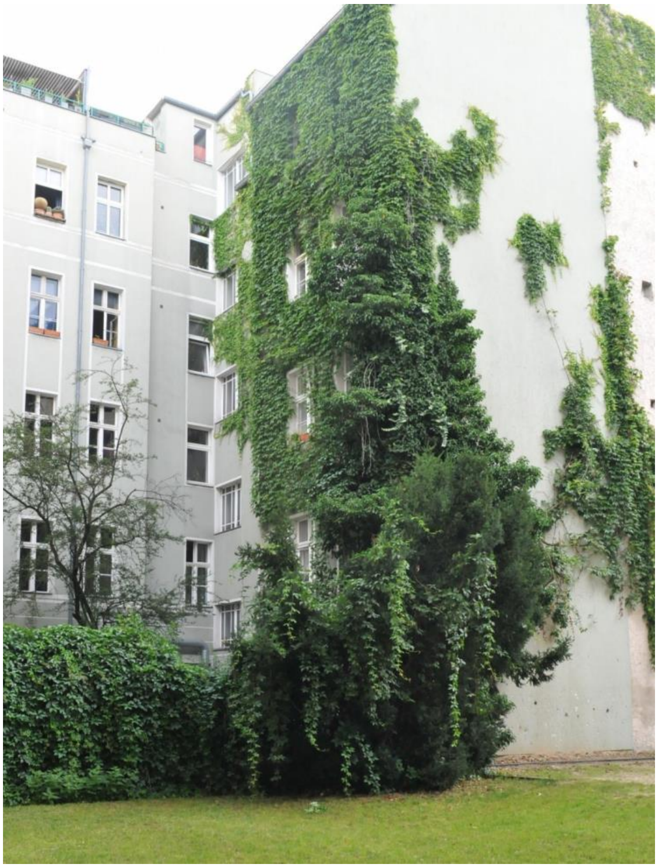

3 Wiesen und Wildstauden

Fast jeder, der einen eigenen Garten besitzt, hat sie- die Rasenfläche.
Von Perfektionismus bis hin zur vermooster Freifläche: bei der Vorstellung des perfekten Rasens scheiden sich die Geister.
Fakt ist jedoch, dass ein leuchtend grüner Rasen mit dichtem Bewuchs und ohne „Unkraut“ nur mit viel Aufwand erhalten werden kann. Wer sich einen perfekten grünen Teppich wünscht, muss viel investieren: viel Wasser, Dünger, Pflege und Zeit. Mit naturnaher Gestaltung und Artenvielfalt hat dies nicht mehr viel zu tun.

Auf eine nutzbare Freifläche deshalb zu verzichten ist aber nicht nötig. Freiflächen, die mit krautiger Vegetation bedeckt sind, haben zwar keinen signifikant kühlenden Effekt wie Bäume und Sträucher, dienen aber als Regenwasserversickerungsfläche. Darüber hinaus haben Wiesen eine vorrangig kulturelle Ökosystemdienstleistung.
Spätestens seitdem die amtierende Bundesumweltministerin Saskia Schulze ihren Aktionsplan zum Insektenschutz vorgestellt hat, ist die ökologische Relevanz der Bestäuber in Deutschland allseits bekannt. Nachdem einige Jahre zuvor das Bienensterben für einen veränderten Blickwinkel auf unsere Agrarlandschaft gesorgt hat, ist mit dem derzeitigen Rückgang der Vielfalt unserer Insekten der Aufruf zu Handeln auch in der Mitte der Gesellschaft angekommen.
Wildblumenmischungen und Handbücher zur korrekten Anlage einer sog. „Bienenweide“ sind gefragter denn je.

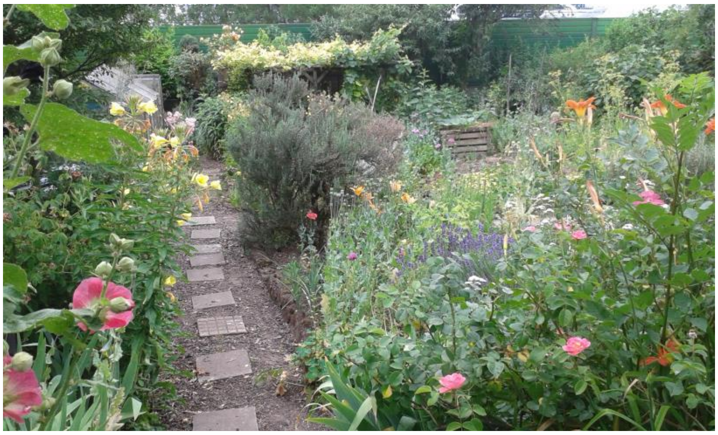

Auch in diesem Leitfaden hat die Wildblumenwiese einen besonderen Stellenwert. Anders als bei den restlichen Maßnahmen liegt der Schwerpunkt hier -neben der Verbesserung des Wohnklimas- insbesondere auf der Bewahrung der Biodiversität am Wohnort.

Dies bedeutet im Umkehrschluss aber nicht die zu vernachlässigende Wirkung von blühenden Mischpflanzungen auf das menschliche Wohlbefinden.

Allerdings ist eine Wiese nicht gleich eine Wiese. Gerade die farbenfrohen Wildblumenwiesen besitzen je nach Saatgut eine Haltbarkeit von bis zu drei Jahren. Die Blühfläche ändert sich mit der Zeit und wird in den Folgejahren anders in Erscheinung treten als zuvor. Für den Erfolg der Maßnahme sind eine gute Vorbereitung des Bodens (spezifisch: eggen) und bis zu zwei Mahden pro Jahr notwendig. Für die korrekte Anlage und Pflege ihrer Wildblumenwiese nutzen Sie Informationen aus Quellen der Landschaftspflege.

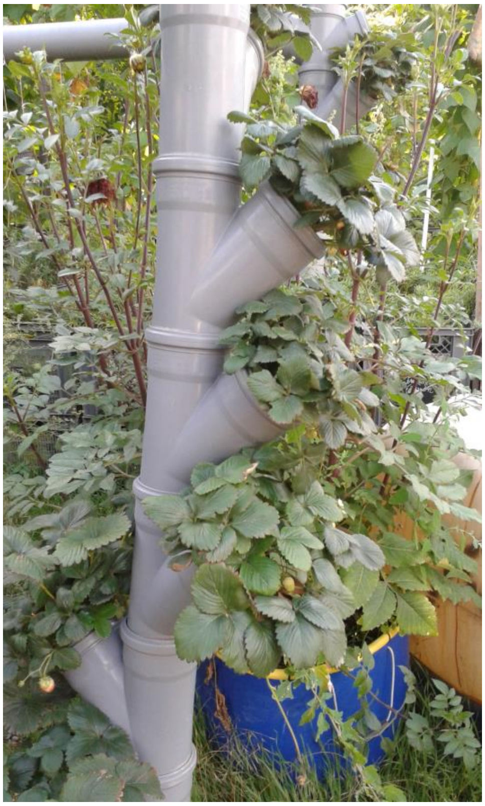

3 Wiesen und Wildstauden

Während mehrjährige Wildblumenwiesen ihren Schwerpunkt als Bienenweide und ihre Ästhetik legen, sind haltbare Wiesen auch von Nutzen für nicht bestäubenden Insekten und können einen erheblichen Beitrag zur biologischen Vielfalt leisten. Bei richtiger Anlage und Pflege können solche Wiesen nahezu unbegrenzt bestehen. Ihr Nachteil liegt jedoch in der aufwändigeren Anlage. Somit muss unter Umständen die Bodenzusammensetzung verändert werden, um eine Grundlage für die gewünschte Vegetation zu bilden. Hinzu kommt, dass vorhandene Vegetation restlos aus dem Boden entfernt werden muss, um eine erneute Besiedlung zu vermeiden.

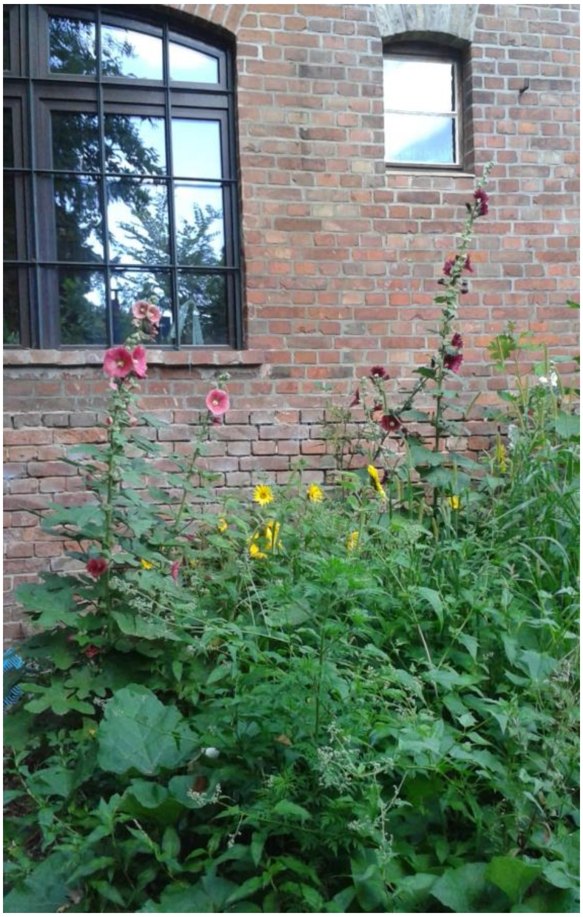

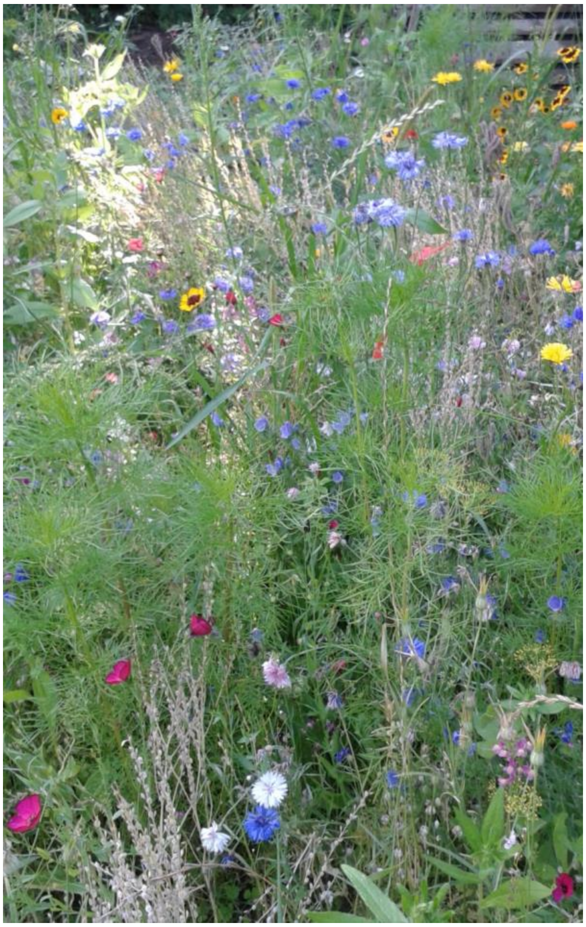

Um das ökologische Gleichgewicht der Wiese nicht zu stören, ist sie zudem nicht als Spielfläche geeignet und sollte nur selten bis gar nicht betreten werden. Bei vorhandenen Allergien gegen Gräser kann es zudem zu gesundheitlichen Beschwerden bei den Bewohnern kommen- dies gilt es bei der Planung zu beachten.

3 Wiesen und Wildstauden

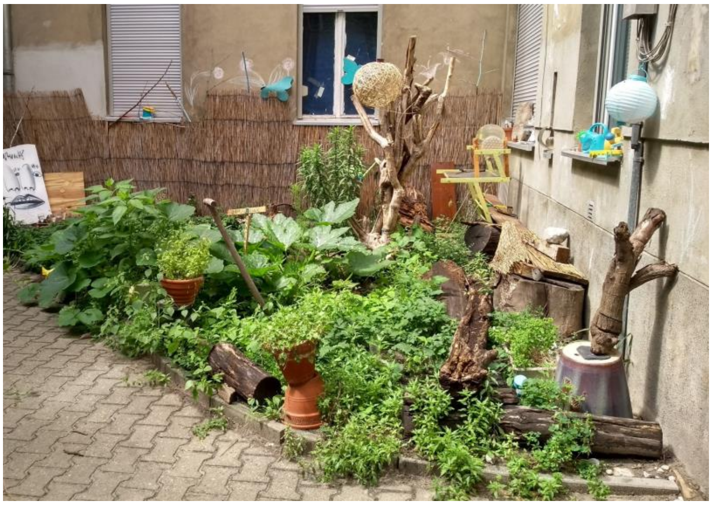

Wildstauden als Alternative bei Platzmangel

Bei kleinen Grünflächen im inneren Stadtkern sind solche Anlagen nur bedingt empfehlenswert. Fehlt der Platz für eine mehrjährige Wildblumenwiese sind Alternativen wie blühende Hecken (siehe: Sträucher, Hecken und Büsche) und Randsäume, sowie Stauden denkbar. Die Vorteile dafür liegen auf der Hand:
Wildstauden wie Schafgarbe, Wiesensalbei oder Lavendel lassen sich problemlos in Hochbeete oder Kübel pflanzen und in sonnenexponierte Stellen eines Innenhofes stellen. Somit ist diese Alternative äußerst platzsparend und ein offener Boden ist keine Voraussetzung. Insbesondere bei versiegelten Innenhöfen helfen sie, den vorhandenen Raum aufzuwerten. Beachten Sie bei der Auswahl der Arten die Wurzeltiefe und maximale Wuchshöhe, sowie die Dauer der direkten Sonneneinstrahlung.

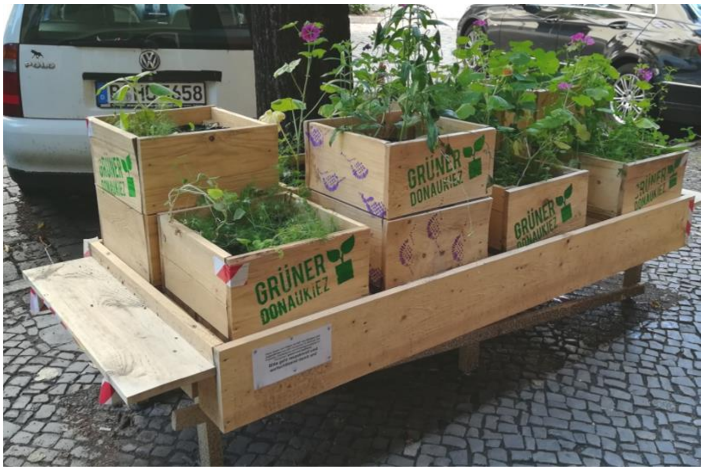

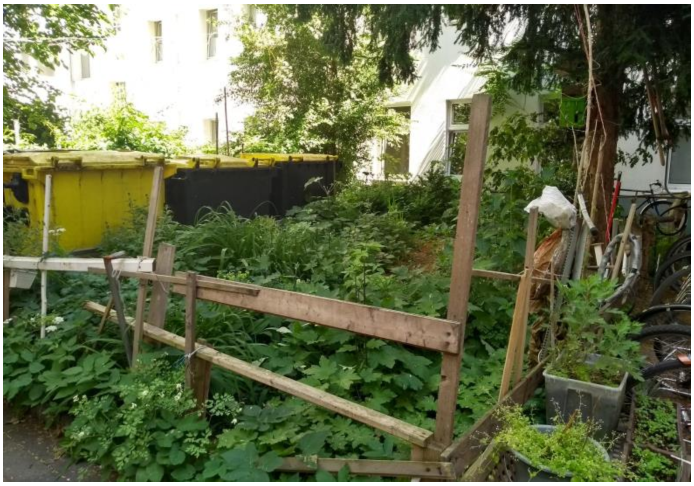

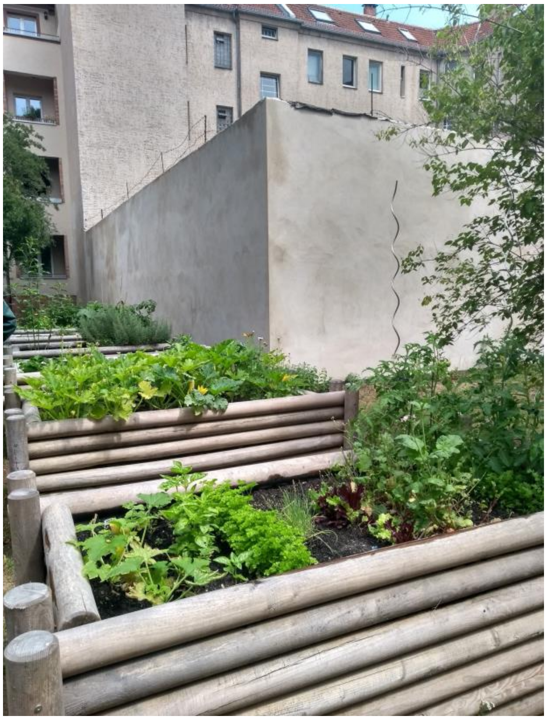

3 Rasenflächen aktiv gestalten

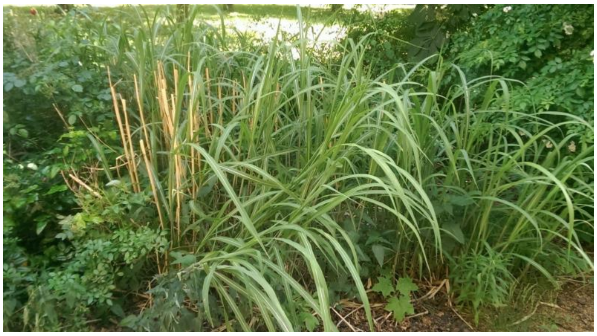

Welche große Bedeutung die (Stadt-)natur auf Kinder und ihre Entwicklung hat, lässt sich ausführlich im TEEB Naturkapital Deutschland- „Grün in der Stadt"; sowie in den BfN-Skripten 203 „Kinder und Natur in der Stadt" nachlesen.

Im Rahmen einer zunehmenden „Verhäuslichung" der Kinder sind insbesondere Spielplätze in naturnahen Umgebungen von großer Bedeutung. Dabei ist nicht die Ausstattung und Komplexität technischer Spielelemente von Bedeutung, sondern das alleinige Vorhandensein von Spielräumen. Gerade naturnahe Gelände bieten dabei einen Raum zur phantasievollen Gestaltung der Umwelt, wo der kindliche Entdeckungsdrang ausgelebt werden kann.
Eine Kombination aus technischen Spielelementen wie Schaukeln, Rutschen und Klettergerüsten und naturnahen Flächen lässt sich je nach Ausführung gut auf fast allen Freiflächen umsetzen.
Wilde Spielflächen können auf unterschiedliche Weise so gestaltet werden, dass sie altersübergreifend ansprechend wirken.

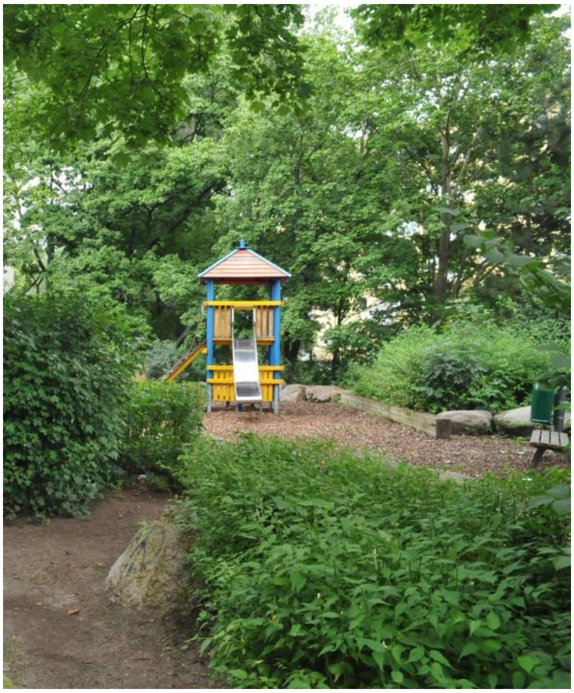

Umsetzungsideen:

- Schaukeln, Rutschen und Klettergerüste benötigen bei versiegelten Untergründen eine geeignete Unterlage, um Verletzungen vorzubeugen
- Sandkästen finden in jeder Außenanlage Platz und sind überall leicht zu integrieren, unabhängig vom Untergrund
- ungleichmäßige Rasen- und Wiesenflächen bieten nicht nur einen Vorteil für die vorhandene Flora und Fauna- sie werden auch von Kindern zum Rennen, Springen und Toben genutzt.
- Im hohen Gras einer Wiese lässt es sich gut verstecken, während auf Rasenflächen viel gerannt und gespielt werden kann (siehe „Rasen" und „Wiese")
- Gleiches gilt für pflanzliche Elemente wie Bäume und Sträucher- sie bieten eine Vielzahl an Möglichkeiten, die Kreativität der Kinder zu wecken, sei es zum Verstecken, Klettern als auch zum Aufspannen von Hängematten oder Tragseilen.
- Einen besonderen Stellenwert haben hier Weidenbauten als natürliche Spielelemente im Wohnumfeld, sie können von Anwohnern eigenständig angelegt werden
- Bei größeren Freiflächen empfiehlt es sich, einen Mehrgenerationenpark (auch: Bewegungs-parcours) anzulegen. Dieser wird neben den klassischen Spielelementen noch um Fitnessgeräte ergänzt, die Besucher jedes Alters zur Bewegung und Entspannung animiert. Ziel ist es, generationsübergreifend Spaß und Freude an körperlicher Betätigung zu vermitteln

3 Gewässer

Teiche sind zweifelsohne ein Highlight in jedem Garten. Gut angelegt sind sie ein Hotspot der Biodiversität und erfreuen nicht nur Stadttiere. Als stetige Wasserquelle werden sie zum Magneten für Vögel, kleine Säuger und Insekten, sowie Bewohner, die dem regen Treiben im und um den Teich herum zuschauen. Gleichzeitig leistet der Teich mit seiner Vegetation einen Beitrag zur Verbesserung des Bioklimas und fungiert als CO2-Senke.
Voraussetzung für die Anlegung eines Teiches ist der entsiegelte Boden. Aus diesem Grund, eignet sich ein naturnaher Teich nur bei Stadtstrukturtypen, die über genügend Freifläche mit entsiegeltem Boden verfügen, vorzugsweise Brachfläche oder Rasen ohne weit verwurzelte Gehölze. Die Anlegung eines Teiches sollte nur mit fachkundiger Hilfe eines Experten erfolgen, der sich zusätzlich mit den geologischen Gegebenheiten des Standortes auskennt (Stichwort Grundwasserstand, Leitungen etc.).
Achten Sie bei der Umsetzung auf die ökologische Funktionalität. Um ein stabiles Ökosystem zu erschaffen ist die richtige Zonierung in Flach- und Tiefwasserzonen und die Pflanzung verschiedener Wasserpflanzen von Bedeutung. Der Standort des Gewässers hat ebenso einen Einfluss auf den Nährstoffeintrag, hier spielt die Beschattung/Sonnenexposition und der Ein-trag von Laub eine große Rolle. Zu den Pflegemaßnahmen gehört deshalb die fachgerechte Entsorgung von Müll und organischem Material, sowie die Pflege der Wasserpflanzen.
Ein Teich ist somit sicher eine der aufwändigsten Maßnahmen zur Umgestaltung des Wohnumfelds, wird aber genauso schnell zum unangefochtenen sozialen und ökologischen Mittelpunkt einer jeden Gartenanlage. Werden Teile des Teiches mit Stauden und Sträuchern gerahmt, werden Rückzugsorte und Korridore für Kleintiere geschaffen. Sitzgelegenheiten erfreuen Bewohner und verleiten zum Verweilen, Innehalten und Beobachten.
Stellen sie sicher, dass es keine abbruchsgefährdeten Stellen gibt, an denen Kinder direkt in tiefere Teichabschnitte rutschen können. Eine gut angelegte Zonierung mit Flachwasserzonen und durch Sträucher unzugänglich gemachte Uferbereiche können Teiche für Kinder sicherer machen. Des weiteren können grobmaschige Eisengitter auf Teile des Teiches gelegt werden, die keine Barriere für Vegetation schaffen, aber einen Sturz auffangen. Erkundigen Sie sich, ob Tiergeräusche (z.B. Frösche) von der Mehrzahl der Bewohner als störend empfunden werden.

Verfügt ihr Wohngebiet nicht über die anspruchsvollen Voraussetzungen einer Teichanlage, kann eine einfache Wasserquelle in Form von größeren Vogeltränken eine ähnliche Anziehungskraft für Bewohner und Tiere entwickeln. Insbesondere im innerstädtischen Bereich, wo der Versiegelungsgrad hoch ist und frei zugängliche Gewässer rar sind, können sie für Vögel, Insekten und kleine Säugetiere lebensnotwendig sein.
Stellen Se sicher, dass die Vogeltränke an einem einsehbaren Platz steht, wo sich die Tiere sicher fühlen und diese annehmen. Vogeltränken, die sich nicht unmittelbar am Boden, sondern auf einer Erhöhung befinden, machen diese weniger attraktiv für „ungewollte Besucher“ wie Ratten und Mäuse- aber auch Igel. Auch eine simple Tränke benötigt Pflege, um Vögel vor Krankheiten zu schützen. Sie sind regelmäßig zu reinigen und das Wasser zu erneuern. Dabei ist der Einsatz von Chemie unnötig und sogar kontraproduktiv. Für die Reinigung reicht eine Behandlung mit grober Bürste und heißem Wasser, sowie das eintägige Austrocknen der Tränke an der Sonne, bevor frisches Wasser nachgefüllt wird.

3 Gehölzpflanzungen

Laub- und Nadelbäume

Bäume sind wichtige Bestandteile des Wohnumfeldgrüns. Für viele Menschen gehören Bäume zu ihrer Vorstellung von Natur. In aktuellen Publikationen wird immer häufiger auf die Verbindung zwischen Bäumen und mentaler Gesundheit hingewiesen. Der Aufenthalt im Wald führt bei Menschen nachweislich zu einem gesteigerten Wohlbefinden und Resilienz. So können Stadtbäume dazu beitragen, mentalen Stress zu reduzieren. Doch nicht nur die mentale Gesundheit profitiert von Stadtbäumen. Insbesondere für das Urbane Klima, können Bäume einen erheblichen positiven Beitrag leisten, thermische Belastungen auszugleichen. Durch das Laub in der Vegetationsperiode verringert sich die Sonneneinstrahlung, die auf versiegelten Boden und Gebäude auftrifft, und das damit verbundene Aufheizen des Umfelds. Gleichzeitig wird die Luft durch Transpiration abgekühlt. Insbesondere die Verbesserung des Bioklimas wird von Anwohnern als angenehm empfunden.
Wohnungen, die sich nahe einer viel befahrenen Straßen befinden, profitieren zudem von einer leichten Geräuschminderung, sowie von der verbesserten Luftqualität durch die Umwandlung von $CO2$ in Sauerstoff, als auch der damit verbundenen Filtration von Luftschadstoffen.

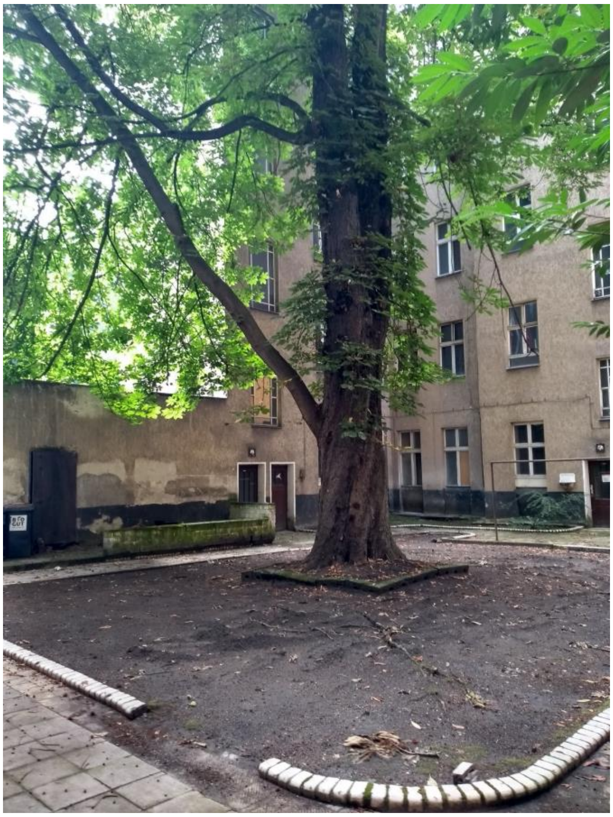

Von allen Maßnahmen werden Stadtbäumen daher ein besonderer Stellenwert zugeschrieben, da die dazugehörigen Ökosystemleistungen sehr vielfältig sind.
Bereits die vereinzelte Pflanzung von Bäumen ist eine gute Investition für das Wohnumfeld. Bäume sind relativ pflegeleicht und erreichen je nach Art ein hohes Alter. Auch an stark versiegelten Orten schaffen sie grüne Inseln und werden als solche auch von der belebten Umwelt als Trittstein oder Inselbiotop genutzt. Mit Nistkästen für Fledermäuse und Vögel kann dem nachgeholfen werden.
Bei nahezu jedem Standort ist es deshalb angeraten, sich über die Möglichkeiten von Baumpflanzungen zu informieren. Bereits kleine Innenhöfe werden durch Bäume enorm aufgewertet. Versiegelte Böden können mit einer Mindestfläche von 2x2,5m pro Baum teilentsiegelt werden.

3 Gehölzpflanzungen

Bei der Artauswahl ist auf die Ansprüche der jeweiligen Art zu achten, was Nässe und Trockenheit des Bodens betrifft. Da der Boden in städtischen Gebieten häufig verdichtet vorliegt, kann es sinnvoll sein, mit speziellen Vorrichtungen (z.B. Schläuchen), für ausreichend Belüftung und Bewässerung zu sorgen. Bei Nähe zu Leitungen kann eine Wurzelsperre eingebaut werden. Hierbei gilt es, zwischen Tiefwurzlern und Flachwurzlern (div. Nadelbäume) zu unterscheiden.

Des Weiteren gilt bei der Artauswahl das jeweilige Wuchsverhalten zu beachten- Linden und Eichen erreichen eine stattliche Höhe von bis zu 25 Metern, während Obstbäume wie Kirsche und Apfel selten über 15 Meter hinauswachsen. Grade die Anpflanzung von Obstbäumen kann als ein gärtnerisches Element in Urban Gardening integriert werden. Des Weiteren dienen Obstbäume in der Blütezeit als hervorragende Bienenweide. Die Entfernung von Laub im Herbst gehört zu den pflegerischen Maßnahmen, die über die Lebenszeit des Baumes besteht.
Nadelbäume wie Kiefer und Fichten verlieren ihre Nadeln zwar nicht im Herbst und erscheinen immergrün, sind jedoch bei Kleinkindern mit Vorsicht zu genießen und nicht immer in direkter Nähe eines Sandkastens oder Spielplatzes empfehlenswert, da die ätherischen Öle der Nadeln je nach Nadelbaum nicht gesundheitsfördernd sind. Sie sind jedoch als eine gute Ergänzung zu Laubbäumen zu betrachten.

Gesundheitliche Nachteile, die von Bäumen ausgehen können, sind meist mit Allergien gegen Hasel und Birke verbunden. Nach Möglichkeit empfiehlt es sich, hierbei auf andere Bäume auszuweichen.
Ein besonderer Hinweis gilt der Pflanzung von Gingko-Bäumen als ästhetisches Element: Durch seine Anspruchslosigkeit hat sich Gingko als Stadtbaum etabliert, jedoch verfügen die Samen der weiblichen Pflanzen über Buttersäure, was im Herbst zu einer erheblichen Geruchsbelästigung führen kann. Bei bestehendem Wunsch nach Gingko sollte deshalb auf männliche Pflanzen ausgewichen werden.
Neben der Pflanzung von großen und kleinen Bäumen ist auch die ergänzende Pflanzung von Sträuchern als strukturgebende und platzsparende Elemente empfehlenswert.
Die genauen Richtlinien für eine gelungene Gehölzpflanzung entnehmen Sie den Angaben der FLL (Forschungsgesellschaft Landschaftsentwicklung Landschaftsbau e. V.).

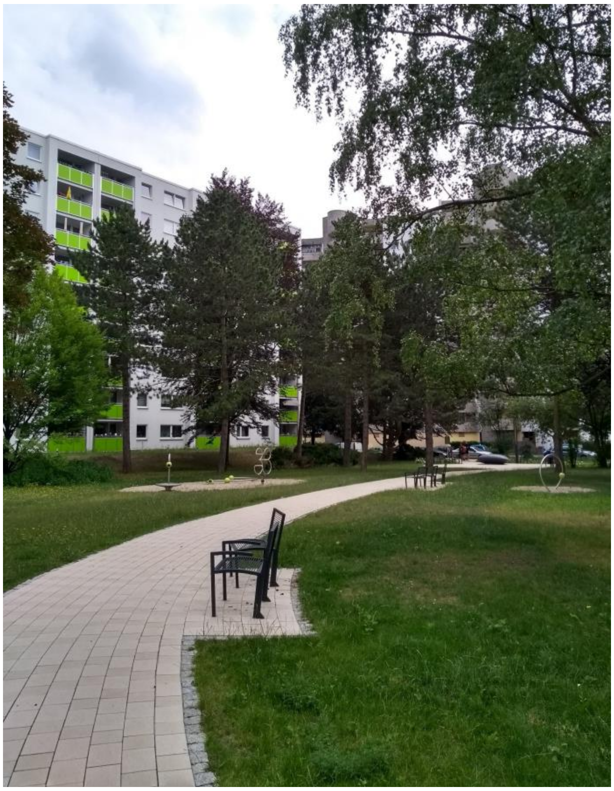

3 Von Sträuchern und Hecken

Unter Sträuchern (ugs. Gebüsch) versteht man im Allgemeinen Pflanzen ohne durchgehenden Hauptstamm, deren Triebe (im Gegensatz zu Stauden) verholzen. Halbsträucher besitzen einen verholzten Ansatz, wobei die unverholzten Zweige nach jeder Vegetationsperiode absterben und im Folgejahr erneut austreiben. Während Bäume einen Hauptstamm aufweisen, haben Sträucher viele bodennah verzweigte Stämme.

Ist nur geringer Platz vorhanden, lassen sich kleinere Sträucher- ähnlich wie Stauden- in Kübel einpflanzen und einen Innenhof oder Randgebiete verschönern.

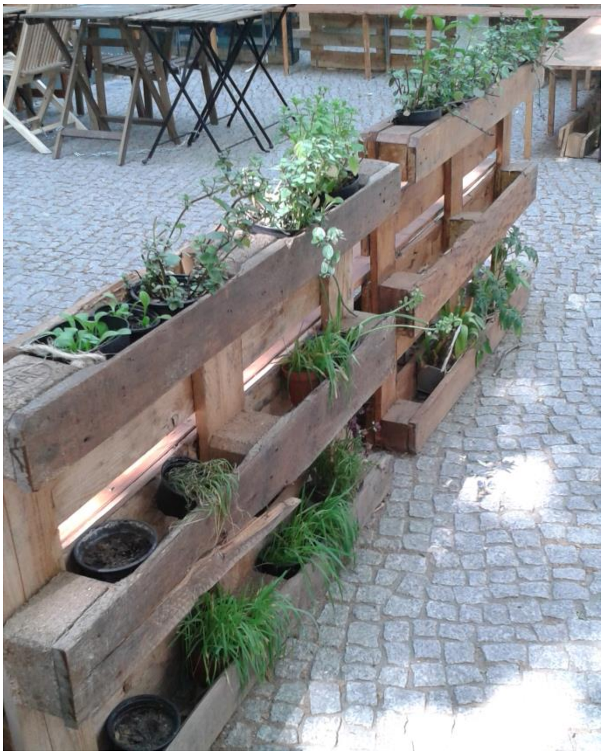

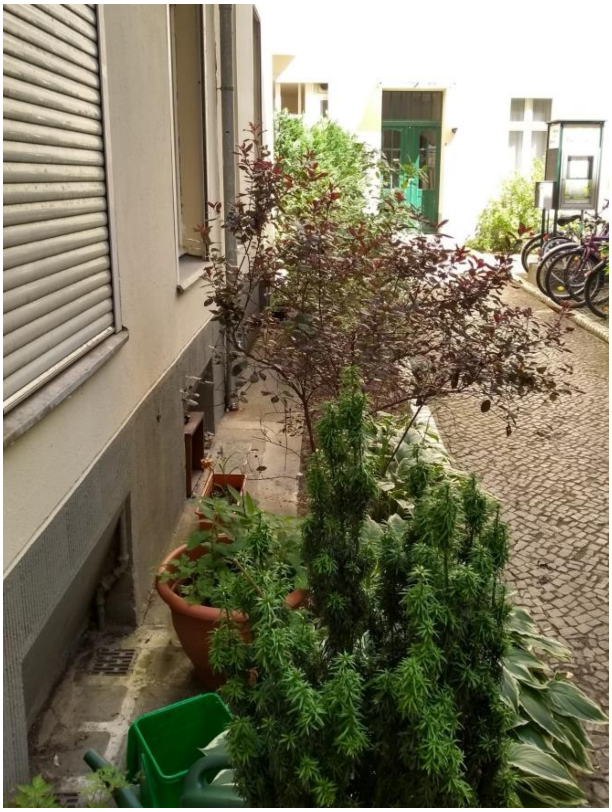

Der gesundheitliche Vorteil von Sträuchern liegt, wie auch bei Gehölzen, in ihrer Funktion als Primärproduzenten. Sie tragen zur Verbesserung der Stadtluft bei und sorgen für ein angenehmeres Mikroklima.

Im Gegensatz zu Bäumen ist die Schattenwurfrate gering, das bedeutet, dass selbst auf kleinen Räumen und nah an Gebäuden gepflanzt werden kann, ohne dass das Laub in der Vegetationszeit zu viel Schatten in die angrenzenden Wohnungen wirft.

Sträucher können somit auf beengtem Raum punktuell als Ersatz für größere Gehölze gepflanzt werden. Gleichzeitig geben sie einen Rückzugsort für kleinere Tiere

3 Von Sträuchern und Hecken

Am häufigsten werden Sträucher als strukturgebende Elemente auf Freiflächen verwendet. Sie dienen als Sichtschutz und Abgrenzung. Ein Spielplatz lässt sich damit ebenso abgrenzen wie auch die hauseigenen Mülltonnen. Immergrüne Gewächse sind dabei empfehlenswert

Bei weiträumigeren Stadtstrukturtypen wie Zeilen- und Plattenbauten werden Grundstücksgrenzen und großflächig eingerahmt. Immergrüne Gehölze, sowie Hainbuche (streng genommen kein Strauch, wird jedoch als solcher geschnitten und gestutzt) sind hier empfehlenswert.

Alternativ zu Betonmauern und Maschendrahtzäunen können bei vorhandenem Platz lebende Hecken mit einer ausgeprägten Saum-Mantel- und Kernzone entstehen, die als Linienbiotop Unterschlupf für Vögel und Säugetiere bieten.

Neben lebenden Hecken bieten sich auch Gebilde wie Totholzhecken (sog. Benjeshecken) bestehend aus Gehölzschnitten, sowie Trockensteinmauern an. Letztes sind vor allem für Insekten von hohem Stellenwert. Sie können auch als integrativer Teil von Sitzelementen gestaltet werden, vorausgesetzt, ihre Standfestigkeit wird regelmäßig überprüft. Beide Varianten bestechen durch geringen Pflegeaufwand und eine lange Haltbarkeit

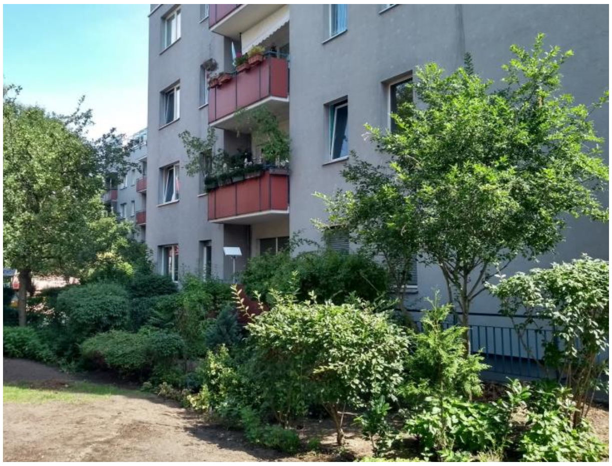

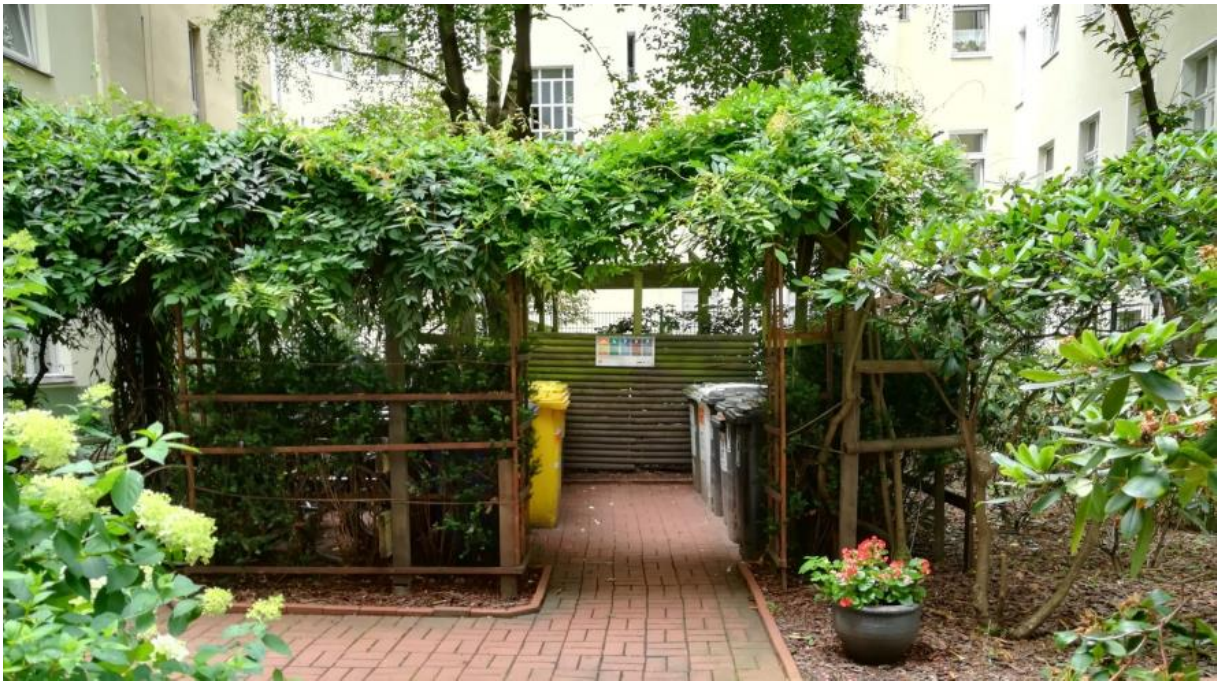

3 Wege, Parkplätze und Randflächen

Neben den zuvor genannten Maßnahmen gibt es noch weitere Möglichkeiten, den Außenbereich ökologischer zu gestalten. Insbesondere, wo zu wenig Platz vorhanden ist um großflächig aktiv zu werden, können kleine Änderungen das Wohnumfeld aufwerten und somit zu einem verbesserten Wohlbefinden der Bewohner führen. Insbesondere die Entsiegelung des Bodens steht bei dieser Ideensammlung im Vordergrund.

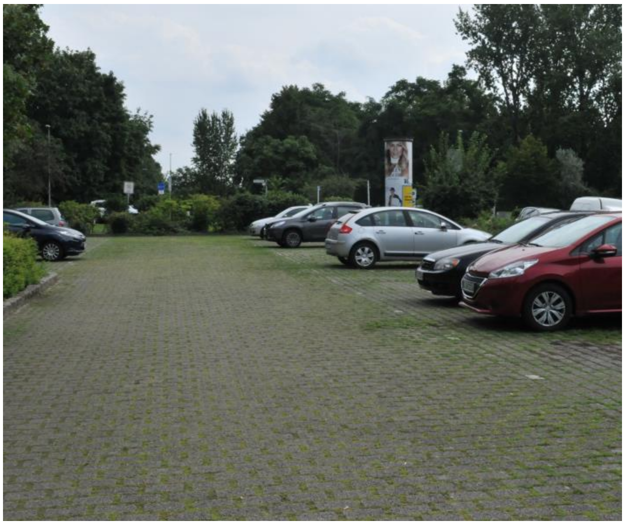

Feuerwehrzufahrten

Auf Unternehmensflächen sind sie Pflicht: Feuerwehrzufahrten müssen nach Brandschutzrecht für Rettungskräfte mit bis zu 16 Tonnen Gesamtgewicht zugängig und befahrbar sein. Ein Grund für eine asphaltierte Fläche ist es jedoch nicht.
Hier eignet sich ein nährstoffarmer Schotterrasen als alternativer Untergrund. Standortgerechte Wildblumen- und Kräuter unter 50 cm bieten Insekten eine Lebensgrundlage und sorgen für ein ästhetischeres Gesamtbild.

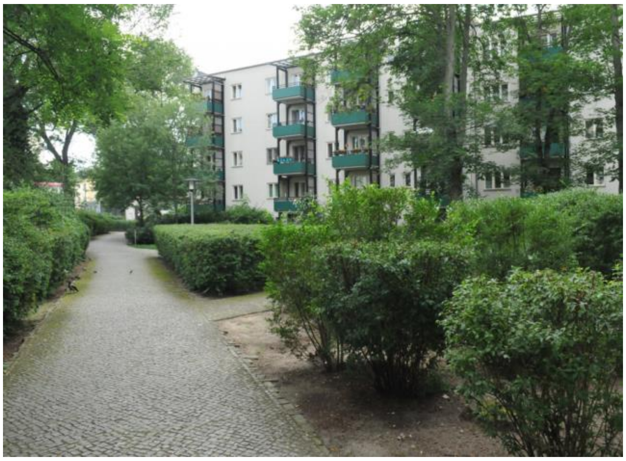

Wege

Anstelle von asphaltierten Wegen sind hier Splittfugen, Naturstein oder Schotter als wassergebundene Decke empfehlenswert, welche Wasserabfluss ermöglichen.
Bei der Gestaltung sind neben dem naturnahen Ansatz auch die Barrierefreiheit und Verkehrssicherheit zu beachten.

Parkplätze

Grade bei Wohnhäusern, deren Bau sich auf die zweite Hälfte der letzten Dekade datieren lässt, sind großräumige Parkplätze für Anwohner vorhanden. Bei großflächigen Versiegelungen dieser Art steht Wasserdurchlässigkeit im Vordergrund. Die Auswahl des Untergrundes ist dabei abhängig von der Frequenz, in welcher die Parkflächen angesteuert werden.
Stark genutzte Parkflächen werden durch Betonpflaster mit Rasen- oder Splittfugen wasserdurchlässiger und garantieren einen belastbaren Untergrund. Bei geringer Nutzung kann auf Schotterrasen zurückgegriffen werden; er besitzt eine höhere ökologische Wertigkeit.
Zwischen einzelnen Stellplätzen spenden Bäume Schatten, was im Sommer eine Aufheizung der Parkflächen und der PKWs verringert. Empfehlenswert sind Ulmen, Eichen und Hainbuchen. Lindenblüten führen zu verklebten Scheiben und sollten deshalb gemieden werden. Von Hecken gesäumt lassen sich Parkplätze in das grüne Gesamtbild einfügen.

Hinweise:
Die Darstellung der Maßnahmenumsetzung stellt lediglich Empfehlungen auf Basis fachlich relevanter Literaturrecherche dar. Trotz größter Sorgfalt kann es zu inhaltlichen Abweichungen in anderen Quellen kommen.
Die dargestellten Inhalte haben erläuternden Charakter und ersetzen im Einzelfall keine fachkundliche Beratung mit Verantwortlichen. Bitte Informieren Sie sich vor der Umsetzung bei Ihrem Planungsbüro.

Stand: April 2019

Bearbeitung und Durchführung:
Sonja Mohr-Stockinger

HealthyLiving Projekt:
Dr. Ina Säumel, IRI THESys, Humboldt Universität Berlin
Dr. Sylvia Butenschön, Institut für Stadt- und Regionalplanung, Technische Universität Berlin

Bilderverzeichnis:
Ewelina Skulimowska: Seiten 1, 5, 7, 8, 9, 10, 13, 14, 17, 18, 20, 22, 23 und 24.
Hannah-Lea Schmid: Seiten 2, 8, 9 und 21.
Renate Mohr: Seiten 15, 16, 17 und 22.
Sonja Mohr-Stockinger: Seiten 11 und 13

Literatur und Links:

https://www.stadtentwicklung.berlin.de/umwelt/umweltatlas/
https://www.fll.de/
https://www.futurelandscapes.tu-berlin.de/menue/healthyliving_projekt/
http://www.kuras-projekt.de/
https://www.ufz.de/teebde/
https://www.gebaeudegruen.info/
https://stadtundgruen.de/fokus/deutscher-dachgaertner-verband-ddv.html/
https://www.fbb.de/aktuelles/internationales/details/news/wgin-world-green-infastructure-network/
https://www.db-bauzeitung.de/
https://difu.de/

Exploring the relationship between trees and human stress in the urban environment(2016). J Townsend et al . 42. 146-159.

Nature-Based Solutions to Climate Change Adaptation in Urban Areas. Linkages between Science, Policy and Practice (2017) N Kabisch et al. Springer International Publishing, XI, 342.

Bundesamt für Naturschutz:
Urbanes Grün in der doppelten Innenentwicklung (2016) J Böhm et al. BfN-Skripten 444.
Urbane Grüne Infrastruktur. Grundlage für attraktive und zukunftsfähige Städte. Hinweise für die kommunale Praxis (2017)

	Gründerzeitliche Blockbebauung	Reformorientierte Blockrandbebauung	Zeilensiedlung	Großwohnsiedlungen
Dach-begrünung	Bei Block- und Blockrandbebauung durch die Baustruktur nur selten empfehlenswert. Hier sind private Pflanzungen auf vorhandenen Terrassen vorzuziehen. Sollte die Dachneigung und Statik des Gebäudes eine Dachbegrünung zulassen, beachten Sie auch den Denkmalschutz für das ausgewählte Gebäude		Werden statische Ansprüche erreicht, eignen sich intensive oder extensive Dachbegrünungsmaßnahmen. Sind Flachdächer vorhanden, können Hochbeete ergänzt werden.	Die meisten Gebäude dieser Strukturen eignen sich am besten für Dachbegrünungsmaßnahmen. Durch Hochbeete ergänzt, werden sie zum neuen ökologischen Mittelpunkt der Wohneinheit für Anwohner
Fassaden-begrünung	Durch einen hohen Versiegelungsgrad und den geringen Anteil bodennaher Freiflächen, sind Fassadenbegrünungen hier sehr empfehlenswert. Bitte beachten Sie auch hier den Denkmalschutz des Gebäudes und bevorzugen Sie bei bodengebundenen Systemen Rankhilfen. Bei poröser Baustruktur ist es wichtig, das Wurzel- und Spitzenwachstum der Ranke im Auge zu behalten und diese regelmäßig zurückzuschneiden.		Auch hier gilt: Fassadenbegrünung ist eine einfache Option, Grün im Wohnumfeld zu integrieren und sollte in jedem Fall in Betracht gezogen werden. Selbstklimmer und Gewächse mit Rankhilfen lassen Gebäudezeilen ergrünen. Erlaubt es die Bausubstanz, können wandgebundene Systeme integriert werden.	Modernere Gebäude eignen sich aufgrund der besseren Bausubstanz gut für wandgebundene Systeme. Sollte dies aus finanziellen oder planungstechnischen Gründen nicht umzusetzen sein, sind bodengebundene Systeme äußerst empfehlenswert.
Wiesen und Wildstauden	Gepflasterte oder betonierte Innenhöfe lassen häufig keine Wiesenfläche zu. Des weiteren bestimmen Gebäudehöhe und Hofgröße den Lichteinfall. Wildstauden lassen sich gut in Kübeln oder Hochbeeten ziehen und sollten in jeden Hof integriert werden.	Je nach Hofgröße und Versiegelungsgrad sind Wiesenabschnitte realisierbar. Beachten Sie den Lichteinfall und pflanzen sie zusätzlich Wildstauden. Bei mangelnder Freifläche platzieren Sie Hochbeete oder Kübel in sonnenexponierte Stellen	Grünflächen zwischen den Gebäudenzeilen sorgen für guten Lichteinfall und eignen sich für Wildblumen und Stauden. Erstellen sie große oder kleine Wildblumenbeete und ergänzen Sie diese durch Hochbeete	Durch größere Freiflächen wird die Anlage einer diversen Wildblumenwiese ermöglicht. Stellen Sie sicher, dass diese u.U. trittgeschützt ist, indem Sie andere Teilflächen freigeben.
Freiflächen-gestaltung	Die Gestaltung hängt maßgeblich von der Hofgröße und den Ansprüchen der Bewohner ab. Sind Kinder vorhanden, können Sandkästen und Schaukeln im Hof angebracht werden, was unabhängig vom Versiegelungsgrad geschehen kann. Hochbeete laden zum gemeinschaftlichen Gärtnern ein, während Bänke und Tische die Anwohner nach draußen locken. Schaffen Sie auch auf kleinem Raum eine Möglichkeit zum Aufenthalt und zur Freizeitgestaltung der Anwohner.		Da Zeilenbebauungen oft größere Einheiten umfassen, ist die Bewohnerdiversität besonders hoch. Mehrgenerationenparks werden hier besonders empfohlen	Die Bewohnerdichte und damit verbundene Diversität kann durch ein vielschichtiges Angebot an Gestaltungselementen bedient werden. Schaffen Sie soziale Räume durch Sitzgelegenheiten, Mehrgenerationenparks, und die gemeinsame Bewirtschaftung von z.B. Gemüsebeeten
Gewässer	Gewässer sind – in Abhängigkeit von Größe und Versiegelung des Hofes- schwer zu integrieren und nehmen viel Fläche in Anspruch. Nichtsdestotrotz sind Wasserquellen für Stadttiere unverzichtbar- Stellen Sie Vogeltränken zu Verfügung	Bei größeren Innenhöfen lassen sich kleine Gewässer gut integrieren. Ist dies nicht möglich, schaffen sie andere Wasserquellen für Stadttiere in Form von Vogeltränken.	Ist der Platz zwischen den Gebäudezeilen ausreichend, lassen sich mehrere kleinere Gewässer in Form von Brunnen oder Teichen integrieren.	Gewässer schaffen auf größeren Freiflächen einen ökologischen und sozialen Hotspot und sollten aus diesem Grund bevorzugt werden.
Gehölze	Gehölze benötigen eine versiegelungsfreie Fläche von mind. 5m², besser sind jedoch 6m² nach DIN 18916. Beachten Sie den Lichteinfall und mögliche Verschattung von niedrig gelegenen Wohnungen in der Vegetationszeit. Die Bodenqualität sollte vor einer Pflanzung bekannt sein.	Durch eine größere Freifläche lassen sich Gehölze gut in Blockrandbebauung integrieren. Achten Sie auf mögliche Verschattung der unteren Wohnungen und einen Mindestabstand zum Gebäude	Niedrige Gehölze lassen sich mehrfach zwischen den Häuserzeilen pflanzen und können durch große Gehölze ergänzt werden. Obstbäume sind bestäuberfreundlich und bieten den Bewohnern auf lange Sicht ein vielseitiges Angebot	Sowohl große als auch kleine Gehölze finden auf größeren Anlagenplatz. Pflanzen Sie verschiedene Obstbäume an denen sich Bewohner jeden Alters erfreuen und integrieren Sie die Pflege wahlweise auch in ein Urban Gardening Projekt.
Sträucher und Hecken	Bei teilversiegelten Böden sind Sträucher eine Alternative zu großen Gehölzen. Strukturieren Sie den Hof, indem Ränder und Stellplätze gesäumt werden. Bei Platzmangel pflanzen Sie Sträucher in größeren Kübeln.	Säumen Sie Stellplätze, Beete und Randstreifen mit Sträuchern, um den Grünanteil zu erhöhen.	Sträucher und Hecken schaffen Struktur zwischen den Gebäudezeilen und können Wege und Stellplätze umrahmen. Die Kombination verschiedener Arten ist hier von ökologischem Vorteil	Integrieren Sie Sträucher und naturnahe Hecken als strukturgebende Elemente. Durch die Pflanzung verschiedener Arten schaffen Sie Diversität.
